# A Model of the Cosmos in the ancient Greek Antikythera Mechanism

**DOI:** 10.1038/s41598-021-84310-w

**Published:** 2021-03-12

**Authors:** Tony Freeth, David Higgon, Aris Dacanalis, Lindsay MacDonald, Myrto Georgakopoulou, Adam Wojcik

**Affiliations:** 1 Department of Mechanical Engineering, University College London (UCL), London, UK; 2 Department of Civil, Environmental and Geomatic Engineering, University College London (UCL), London, UK; 3 UCL Qatar, University College London (UCL), Doha, Qatar; 4 Science and Technology in Archaeology and Culture Research Center (STARC), The Cyprus Institute (CyI), Nicosia, Cyprus

**Keywords:** Environmental impact, Environmental sciences, Astronomy and planetary science, Planetary science, Engineering, Mechanical engineering, Computational science, Pure mathematics

## Abstract

The *Antikythera Mechanism*, an ancient Greek astronomical calculator, has challenged researchers since its discovery in 1901. Now split into 82 fragments, only a third of the original survives, including 30 corroded bronze gearwheels. Microfocus X-ray Computed Tomography (X-ray CT) in 2005 decoded the structure of the rear of the machine but the front remained largely unresolved. X-ray CT also revealed inscriptions describing the motions of the Sun, Moon and all five planets known in antiquity and how they were displayed at the front as an ancient Greek Cosmos. Inscriptions specifying complex planetary periods forced new thinking on the mechanization of this Cosmos, but no previous reconstruction has come close to matching the data. Our discoveries lead to a new model, satisfying and explaining the evidence. Solving this complex 3D puzzle reveals a creation of genius—combining cycles from Babylonian astronomy, mathematics from Plato’s Academy and ancient Greek astronomical theories.

## Introduction

The Antikythera Mechanism is a cultural treasure that has engrossed scholars across many disciplines. It was a mechanical computer of bronze gears that used ground-breaking technology to make astronomical predictions, by mechanizing astronomical cycles and theories^1–9^. The major surviving fragments of the Antikythera Mechanism are labelled A–G and the minor fragments 1–75^7^. They are partial, damaged, corroded and covered in accretions (Supplementary Fig. S1). Nevertheless, they are rich in evidence at the millimetre level—with fine details of mechanical components and thousands of tiny text characters, buried inside the fragments and unread for more than 2,000 years^7^. Fragment A contains 27 of the surviving 30 gears, with a single gear in each of Fragments B, C and D^2,5,7,10^. The fragments are a 3D puzzle of great complexity.

In 2005 *Microfocus X-ray Computed Tomography (X-ray CT)* and *Polynomial Texture Mapping (PTM)* of the Mechanism’s 82 fragments^7^ added substantial data. This led to a solution to the back of the machine^4,7–9^, with the discovery of eclipse prediction and the mechanization of the lunar anomaly^7^ (Supplementary Fig. S20). The front remained deeply controversial due to loss of physical evidence.

Many unsuccessful attempts have been made to reconcile the evidence with a display of the ancient Greek Cosmos of Sun, Moon and all five planets known in antiquity. In 1905–06, remarkable research notes by Rehm^1^ described *Mein Planetarium*, with a ring display for the planets that anticipates the model we present here—but mechanically completely wrong due to his lack of data (Supplementary Fig. S17). In the classic, *Gears from the Greeks*^2^, Price suggested lost gearing that calculated planetary motions, but made no attempt at a reconstruction. Then Wright built the first workable system at the front that calculated planetary motions and periods, with a coaxial pointer display of the Cosmos, proving its mechanical feasibility^3^ (Supplementary Fig. S18). Later attempts by Freeth and Jones^9^ (Supplementary Fig. S19), and independently by Carman, Thorndike, and Evans^11^, simplified the gearing but were limited to basic periods for the planets. Most previous reconstructions used pointers for the planetary displays, giving serious parallax problems^3,9^ and poorly reflecting the description in the inscriptions—see section on *Inscriptional Evidence*. None of these models (Supplementary Discussion S6) are at all compatible with all the currently known data.

Our challenge was to create a new model to match all the surviving evidence. Features on the Main Drive Wheel indicate that it calculated planetary motions with a complex epicyclic system (gears mounted on other gears), but its design remained a mystery. The tomography revealed a wealth of unexpected clues in the inscriptions, describing an ancient Greek *Cosmos*^9^ at the front, but attempts to solve the gearing system failed to match all the data^1–3,6,9^. The evidence defines a framework for an epicyclic system at the front^9^, but the spaces available for the gears are extremely limited. There were also unexplained components in Fragment D, revealed by the X-ray CT, and technical difficulties calculating the phase of the Moon^9^. Then came the discovery in the tomography of surprisingly complex periods for the planets Venus and Saturn, making the task very much harder^12^.

## Discussion and results

We wanted to determine the cycles for *all* the planets in this Cosmos (not just the cycles discovered for Venus and Saturn); to incorporate these cycles into highly compact mechanisms, conforming to the physical evidence; and to interleave them so their outputs correspond to the *customary cosmological order* (CCO), described below. Here we show how we have created gearing and a display that respects the inscriptional evidence: a ring system with nine outputs—*Moon*, *Nodes*, *Mercury*, *Venus*, *Sun*, *Mars*, *Jupiter*, *Saturn* and *Date*—carried by nested tubes with arms supporting the rings. The result is a radical new model that matches all the data and culminates in an elegant display of the ancient Greek Cosmos. With so much missing, we ensure the integrity of our model with a strict set of *Reconstruction Principles* (Supplementary Discussion S1) and we assess the strength of data that validates each element—discussed in Supplementary Discussion S1. The loss of evidence might suggest many options for a model. What has struck us forcefully in making the present model is just how few these options are: the constraints created by the surviving evidence are stringent and very difficult to meet.

## Inscriptional evidence

**Figure 1:**
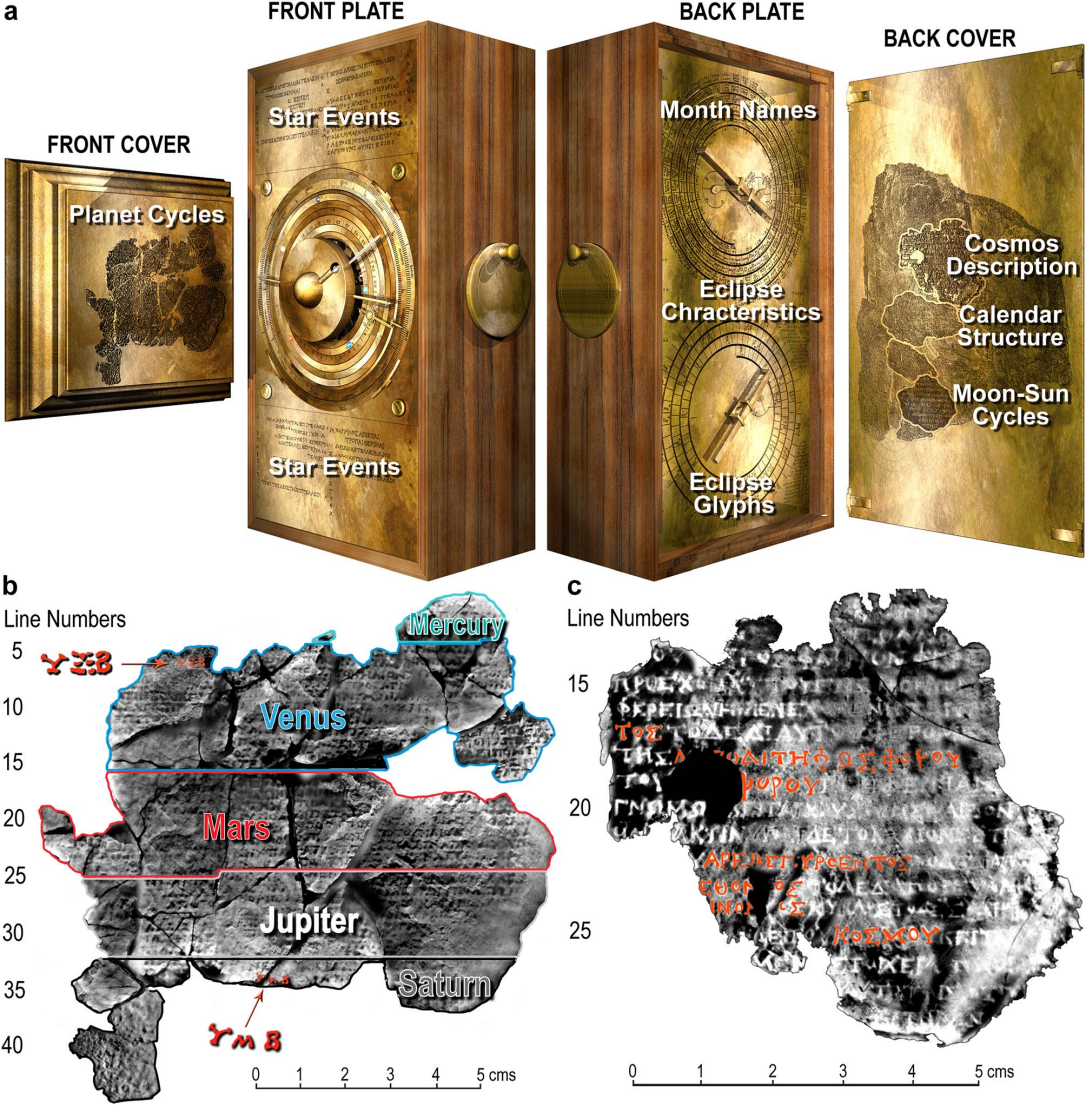
Inscriptions on the Antikythera mechanism. (**a**) FRONT COVER: Planet cycles^9,12^, framed by moulding from Fragment 3 (Supplementary Fig. S5). FRONT PLATE: *Parapegma*^1,2,25^, above and below the Cosmos Display, indexed to the Zodiac Dial. BACK PLATE: Month names on the Metonic Calendar^4,8^. Eclipse characteristics, round Metonic Calendar and Saros Eclipse Prediction Dials^7,8^—indexed to the latter. Eclipse *glyphs* indexed to the Saros Dial^8^. BACK COVER: *User Manual*, including Cosmos description^9,13^ (Supplementary Discussion S2), Calendar Structure^8^ and Moon-Sun Cycles^1,2^. (**b**) Front Cover Inscription (FCI): composite X-ray CT from Fragments G, 26 and 29 and other small fragments^9,12^. The FCI describes synodic cycles of the planets and is divided into regions for each planet in the CCO (Supplementary Discussion S2). The numbers ϒΞΒ (462) in the Venus section and ϒMΒ (442) in the Saturn section are highlighted^12^ (Supplementary Fig. S4). (**c**) Back Cover Inscription (BCI)^13^ (Supplementary Discussion S2): composite X-ray CT from Fragments A and B. A *User Manual*: the upper part is a description of the front Cosmos Display^9^ with planets in the CCO; in red are the planet names as well as the word KOΣMOY—“*of the Cosmos*”.

Reconstructing the Cosmos at the front of the Antikythera Mechanism begins with analysing some remarkable inscriptions. Figure 1 shows the Front and Back Cover Inscriptions (FCI & BCI)^9,12,13^, which are critical for understanding this Cosmos. For previous analysis^13^ and our own line-by-line exploration of the BCI, see Supplementary Discussion S2. The BCI describes the front display as a *Planetarium*^9,13^: a Cosmos arranged in rings, with planets marked by *“little spheres”* and the Sun as a *“little golden sphere”* with *“ray”* and *“pointer”* (Fig. 1c, Supplementary Table S1, Supplementary Figs. S2, S3). The FCI lists the *synodic cycles* of the planets (cycles *relative* to the Sun)^12^. This is a systematic list, itemizing the synodic events and the intervals in days between them. The planets are written in the same geocentric order as the BCI. Adding Moon and Sun gives the *customary cosmological order (CCO): Moon, Mercury*, *Venus, Sun, Mars*, *Jupiter*, *Saturn* (Supplementary Fig. S4), whose origins are discussed in Supplementary Discussion S2.

## Period relations and ancient Greek theories

Ancient astronomers were fascinated by the motions of the planets. As seen from Earth, they exhibit periodic reversals of motion against the stars^14^. In Babylonian astronomy these *synodic cycles* were the basis of planetary prediction^15^, utilizing *period relations*, such as ***5 synodic cycles in 8 years*** for Venus, which we denote by (**5**, **8**). The FCI describes *synodic events*, such as *stationary points*, and intervals between these events (Fig. 1b, Supplementary Fig. S4, Supplementary Discussion S2).

Apollonios of Perga (third-second century BC) created elegant (albeit inaccurate) epicyclic theories to explain these anomalous movements as the sum of two uniform circular motions, their periods defined by period relations—the *deferent and epicycle* models^15^ (Supplementary Discussion S3, Supplementary Figs. S6, S7, S8). Such theories were certainly employed in the Antikythera Mechanism, given that the Moon was mechanized using a similar epicyclic theory^7^. The *true Sun*—the Sun with its variable motion—was also explained in ancient Greece by eccentric and equivalent epicyclic models^14^ (Supplementary Discussion S3).

Babylonian texts list planetary periods and their errors: shorter, less accurate periods in *Goal-Year Texts* (GYT) and longer, more accurate periods in later *Astronomical Cuneiform Texts* (ACT)^15^ (Supplementary Tables S3, S4). The *GYT* periods could have been derived from observations, but not the longer *ACT* periods, such as (**720**, **1151**) for Venus (Supplementary Discussion S3). To understand what period relations were built into the Antikythera Mechanism, the tough problem was to discover their derivation. For Venus the original designer faced a dilemma: the known period relation (**5**, **8**) was very inaccurate, whereas the accurate (**720**, **1151**) was not mechanizable because 1151 is a prime number, requiring a gear with 1151 teeth. Then came a notable discovery in 2016 in the FCI^12^: unexpected numbers ϒΞΒ (**462**) in the Venus section of the FCI and ϒMΒ (**442**) in the Saturn section, translating into highly accurate period relations: for Venus (**289**, **462**) and Saturn (**427**, **442**) (Fig. 1b, Supplementary Fig. S4). Crucially, these are *factorizable*, meaning they can be mechanized with moderate-sized gears, with tooth counts incorporating the prime factors of the period relations. To fit the geometry of the epicyclic system, mechanisms must have gears with < 100 teeth: period relations must have *prime factors* < 100 (Supplementary Discussion S3. There are few such accurate period relations for the planets (Supplementary Tables S5, S6).

The fact that the new period relations for Venus and Saturn from the FCI are factorizable strongly reinforces the idea that they were incorporated into planetary mechanisms in the Antikythera Mechanism^16^. The periods for the other planets are unreadable (in missing or damaged areas of the FCI). To build our model, it was essential to discover the period relations embodied in all the planetary mechanisms. Previous publications^12,16^ derived the Venus period relation (**289**, **462**) as an iterative approximation to the known Babylonian (**720**, **1151**) period relation, using a number of equivalent processes: *continued fractions*, *anthyphairesis* or the *Euclidean algorithm*^17,18^. No similar method for deriving the (**427**, **442**) period relation for Saturn could be found, so this type of iterative approximation was almost certainly not the route to the original discoveries of these periods by the ancient Greeks.

## Discovering cycles in the Antikythera Mechanism

**Figure 2:**
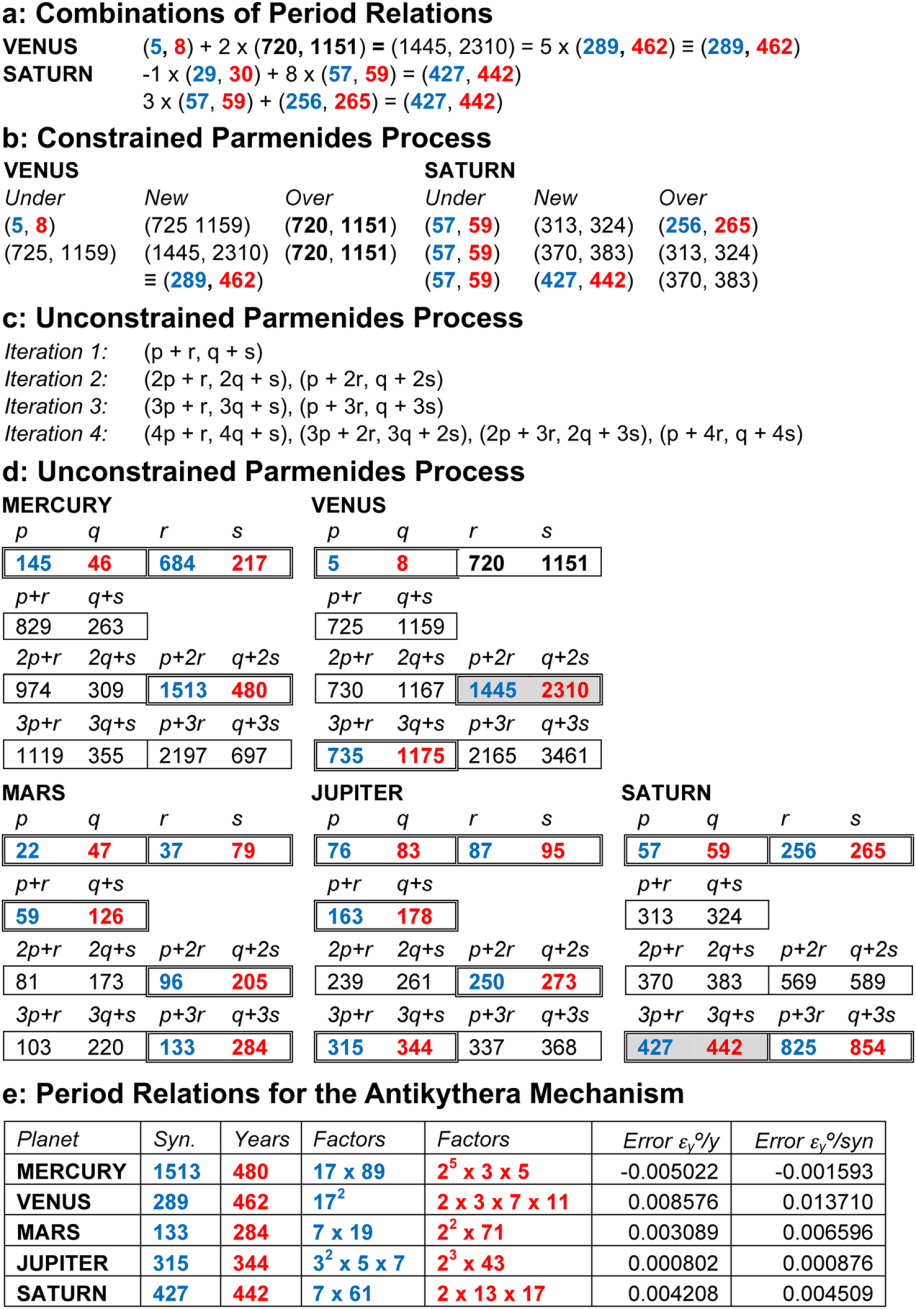
Finding period relations. Blue numbers refer to synodic cycles; red numbers refer to years. All the seed periods for these processes are known from Babylonian astronomy (Supplementary Tables S5, S6). (**a**) Linear combinations of Babylonian period relations, which give those for Venus and Saturn from the FCI. (**b**) Period relations generated by a conventional *Parmenides Process*, which also give those for Venus and Saturn from the FCI. (**c**) Iterations of an *Unconstrained Parmenides Process*. (2p + 2r, 2q + 2 s) is omitted from Iteration 3 because it is the same as 2 x (p + r, q + s). (**d**) Three iterations of the *Unconstrained Parmenides Process*. The pairs in colour are those that are factorizable with prime factors < 100. The grey-shaded periods are those that are known from the FCI. Note that for Venus: (**1445**, **2310**) ≡ (**289**, **462**) and (**735**, **1175**) ≡ (**147**, **235**). The same table with errors is shown in Supplementary Table S5. (**e**) Periods derived from the Unconstrained Parmenides Process for our model of the Antikythera Mechanism and their errors, using our three criteria of *accuracy*, *factorizability* and *economy*. Except for the periods for Venus and Saturn, all the final periods were already known in Babylonian astronomy. The error parameters are defined in Supplementary Discussion S3.

The newly-discovered periods for Venus and Saturn are unknown from studies of Babylonian astronomy. Figure 2 explores how these periods might have been derived. Clues came from the Babylonian use of linear combinations of periods designed to cancel out observed errors^14^. Figure 2a shows how this might generate the periods for Venus and Saturn, but choosing the correct linear combinations *essentially* uses knowledge about errors in known period relations *relative to the true value*. The lack of fine error-estimates from antiquity excludes these methods for our model: errors like < *1° in 100 years* for (**720**, **1151**) were beyond the naked-eye astronomy of the Hellenistic age.

We have developed a new theory about how the Venus and Saturn periods were discovered and apply this to restore the missing planetary periods. A dialogue of Plato^19^ (fifth-fourth century BC) was named after the philosopher Parmenides of Elea (sixth-fifth century BC). This describes *Parmenides Proposition*^17,18^:

- In approximating *θ*, suppose rationals, *p/q* and *r/s*, satisfy *p/q* < *θ* < *r/s*.
- Then (*p* + *r*)/(*q* + *s*) is a new estimate between *p/q* and *r/s*:
  - If it is an underestimate, it is a better underestimate than *p/q*.
  - If it is an overestimate, it is a better overestimate than *r/s*.

Assuming it is a better underestimate, the next stage combines this with the original overestimate to create (p + 2r)/(q + 2s). This would be tested against *q* and the process repeated. Thus, from two *seed ratios* we can generate increasingly accurate linear combinations that converge to *θ*. The Parmenides process is facilitated and constrained by knowledge of *θ* to determine whether each new estimate is an under- or over-estimate. Figure 2b shows how a conventional *Parmenides Process* can generate our target periods, but again this relies on unavailable knowledge about errors. The key step for discovering the missing cycles is to modify the *Parmenides Process*, so it is *not constrained* by knowledge of errors—an *Unconstrained Parmenides Process (UPP)*. Figure 2c, d show the exhaustive linear combinations that are systematically generated by this process. How should we choose which period relations are suitable for our model? Two criteria were surely used for choosing period relations: *accuracy* and *factorizability*. The necessity of fitting the gearing systems into very tight spaces and the ingenious sharing of gears in the surviving gear trains (Supplementary Fig. S20) inspires a third criterion: *economy*—period relations that generate *economical* gear trains, using *shared gears*, calculating synodic cycles with *shared prime factors*^7^ (Supplementary Discussions S3, S6).

Here we clarify how we believe the process was used. The designer would have generated linear combinations using the UPP. At each stage, these possible period relations would have been examined to see if they met the designer’s criteria of *accuracy*, *factorizability* and *economy*. *Factorizability* would have been an easy criterion to assess. *Accuracy* is more problematic, since we do not believe that ancient astronomers had the ability to make very accurate astronomical observations, as is witnessed by the Babylonian records (Supplementary Tables S3, S4). *Economy* must be examined in relationship with the period relations generated for the other inferior or superior planets to identify shared prime factors.

Venus is a good example. The ancient Babylonians knew that the (**5**, **8**) period for Venus was very inaccurate and they had derived the unfactorizable (**720**, **1151**) from observation of an error in the 8-year cycle (Supplementary Discussion S3). Such periods were often described in the ancient world as “exact periods”, though of course in modern terms this is not the case. When the factorizable period (**289**, **462**) was discovered from the UPP, it would have been easy to calculate that it is in fact very close to the “exact period” (**720**, **1151**). Thus, the designer would have been confident that it was an accurate period. (**289**, **462**) would then have been compared with (1513, 480) for Mercury to discover that they shared the common factor 17 in the number of synodic cycles—meaning that they were suitable for use in a shared-gear design to satisfy the criterion of *economy*. When the designer had discovered period relations that matched all the criteria, the process would have been stopped, since further iterations would likely have lead to solutions of greater complexity.

**Figure 3:**
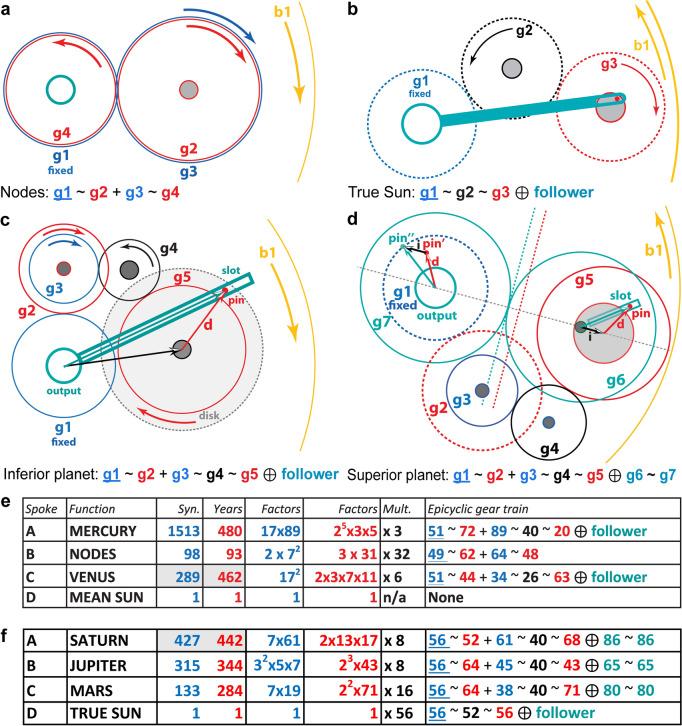
Epicyclic Mechanisms for the Cosmos. Fixed gears are underlined; blue gears calculate synodic cycles; red gears calculate years; black gears are idler gears: all designated by their tooth counts. “ ~ ” means “*meshes with*”; “ + ” means “*fixed to the same arbor*”; “⊕” means “*with a pin-and-follower, turning on the central axis”* or *“with a pin-and-slot on eccentric axes*”—creating variable motion (turquoise). *Followers* are slotted rods that follow a pin on the epicyclic gear and turn on the central axis. For each mechanism, there is a fixed gear at the centre, meshing with the first epicyclic gear, which is forced to rotate by the rotation of **b1** or the **CP**. (**a**) 4-gear epicyclic system for the *Line of Nodes*. (**b**) 3-gear *direct model* for the true Sun. (**c**) 5-gear *direct model* for an inferior planet for complex period relations, with variable motion calculated by a pin and slotted follower. (**d**) 7-gear *indirect model* for a superior planet for complex period relations, with variable motion calculated by a pin-and-slot on eccentric axes. (**e**) Period relations and gear trains on the *Main Drive Wheel*, **b1**; Mercury & Venus share fixed **51**. (**f**) Period relations and gear trains on the *Circular Plate*, **CP**, sharing fixed **56**; gears also shared between Saturn/true Sun and Mars/Jupiter (Supplementary Discussion S4).

The UPP, combined with our three criteria, leads to remarkably simple derivations of the Venus and Saturn period relations. For Venus, Fig. 2d shows that the first factorizable period relation is (**1445**, **2310**) = 5 × (**289**, **462**) ≡ (**289**, **462**) = (**17**^**2**^, **2** × **3** × **7** × **11**), as found in the FCI. For Saturn, it is (**427**, **442**) = (**7** × **61**, **2** × **13** × **17**), again from the FCI. This discovery enables derivations of the missing planetary periods. To ensure our third criterion of *economy*, some of the prime factors of the synodic cycles must be incorporated into the first fixed gear of a planetary train (Supplementary Discussion S4). For Mercury, we are looking for a factor of **17** in the number of synodic cycles to share with Venus. The first factorizable iteration is (**1513**, **480**) = (**17 × 89**, **2**^**5**^** × 3 × 5**)—sharing the prime factor **17** with (**289**, **462**) for Venus—so, a very good choice. Multiplying by integers to obtain viable gears leads to economical designs with a single fixed **51**-tooth gear shared between Mercury and Venus (Fig. 3c, e)^16^. For the superior planets, Mars and Jupiter, we are looking for synodic periods that share the factor **7** with Saturn (Fig. 3d, f). Just a few iterations yield suitable synodic periods—leading to very economical designs with a single **56**-tooth fixed gear for all three superior planets and the *true Sun*.

From Supplementary Table S5,S6, in Supplementary Discussion S3 we establish that the missing periods for Mercury and Mars are uniquely determined by our process. There are two additional options for Jupiter that share the prime **7** in the number of synodic cycles (Supplementary Table S6). In Supplementary Discussion S3 we show how one of these is not possible and the other is very unlikely. The UPP, combined with criteria of *accuracy*, *factorizability* and *economy*, explains the Venus and Saturn periods and (almost) uniquely generates the missing period relations.

## Theoretical mechanisms for our model

The calculation of the Moon’s position in the Zodiac and its phase are defined by surviving physical evidence^7,10^. Since the evidence is missing for the Sun and planets, we need to develop theoretical mechanisms, based on our identified period relations. Figure 3 shows theoretical gear trains for the mean Sun, Nodes and the Planets.

Geometrical parameters for the planetary mechanisms in Fig. 3c, d are shown in Supplementary Table S9.

The way that the Saros Dial on the Back Plate predicts eclipses essentially involves the *lunar nodes*, but they are not described in the extant inscriptions. With their integral role in eclipses, a display of the nodes is a logical inclusion, unifying Front and Back Dials. To maximise the displayed information, we created a mechanism for a hypothetical *Dragon Hand* to indicate the *Line of Nodes* of the Moon, as included in many later astronomical clocks^20^ (Supplementary Fig. S2). We should emphasize that there is no direct physical evidence for an indication of the Line of Nodes of the Moon. We have added this feature as a hypothetical element for the thematic reasons already explained and because it is easily mechanized to good accuracy with a simple 4-gear epicyclic system on Spoke B of **b1**. It is an interesting option for the reader to consider and it coincides with the designer’s apparent ambition to create an astronomical compendium, displaying most of the astronomical parameters that preoccupied Hellenistic astronomy.

All the Cosmos mechanisms must output in the CCO, so that they are consistent with the description in the BCI. At the centre of this Cosmos is the Earth, then the Moon’s position in the Zodiac and lunar phase. The Moon’s position is carried by the central arbor linked to the (mostly) surviving epicyclic system that calculates the Moon’s variable motion at the back of the Mechanism (Supplementary Fig. S1)^7^. We follow the original proposal^10^ for the Moon phase device as a simple differential, which subtracts the motion of the Sun from that of the Moon to calculate the phase, displayed on a small black and white sphere.

A rotation of $^{-{\mathbf{\frac{12}{223}}}}$ for the Line of Nodes, derived from the Metonic and Saros cycles^9^, could not be mechanized because of the large prime **223**. We show that a simpler ratio $^{-{\mathbf{\frac{5}{93}}}}$, with a more accurate period of 18.6 years^14^, can be calculated by a 4-gear epicyclic train (Fig. 3a, Supplementary Figs. S21, S22). This turns a hypothetical double-ended *Dragon Hand*^20^, whose Head shows the *ascending node* of the Moon and Tail the *descending node*.

Using our identified period relations for all the planets, we have devised new theoretical planetary mechanisms expressing the epicyclic theories, which fit the physical evidence. For the inferior planets, previous 2-gear mechanisms^3,9,21^ are inadequate for more complex period relations because the gears would be too large. Two-stage compound trains with idler gears are necessary, leading to new 5-gear mechanisms with *pin-and-slotted followers* for the variable motions^7,9,21^ (Fig. 3c). For the superior planets, earlier models^3,16^ used *direct mechanisms*, directly reflecting epicyclic theories with *pin-and-slotted followers*. Here we propose novel 7-gear *indirect mechanisms* with *pin-and-slot devices*^7,9^ for variable motions (Fig. 3d), analogous to the subtle mechanism that drives the lunar anomaly^7^. Compared to *direct mechanisms*, these are more economical; a better match for the evidence; and incorporate period relations exactly for higher accuracy. The crucial advantages of *indirect mechanisms* are expanded in Supplementary Discussion S4. Without these compact systems that can all be mounted on the same plate, it would have been impossible to fit the gearing into the available spaces. Proofs that the mechanisms in Fig. 3 correctly calculate the ancient Greek epicyclic theories are included in Supplementary Discussion S4.

**Figure 4:**
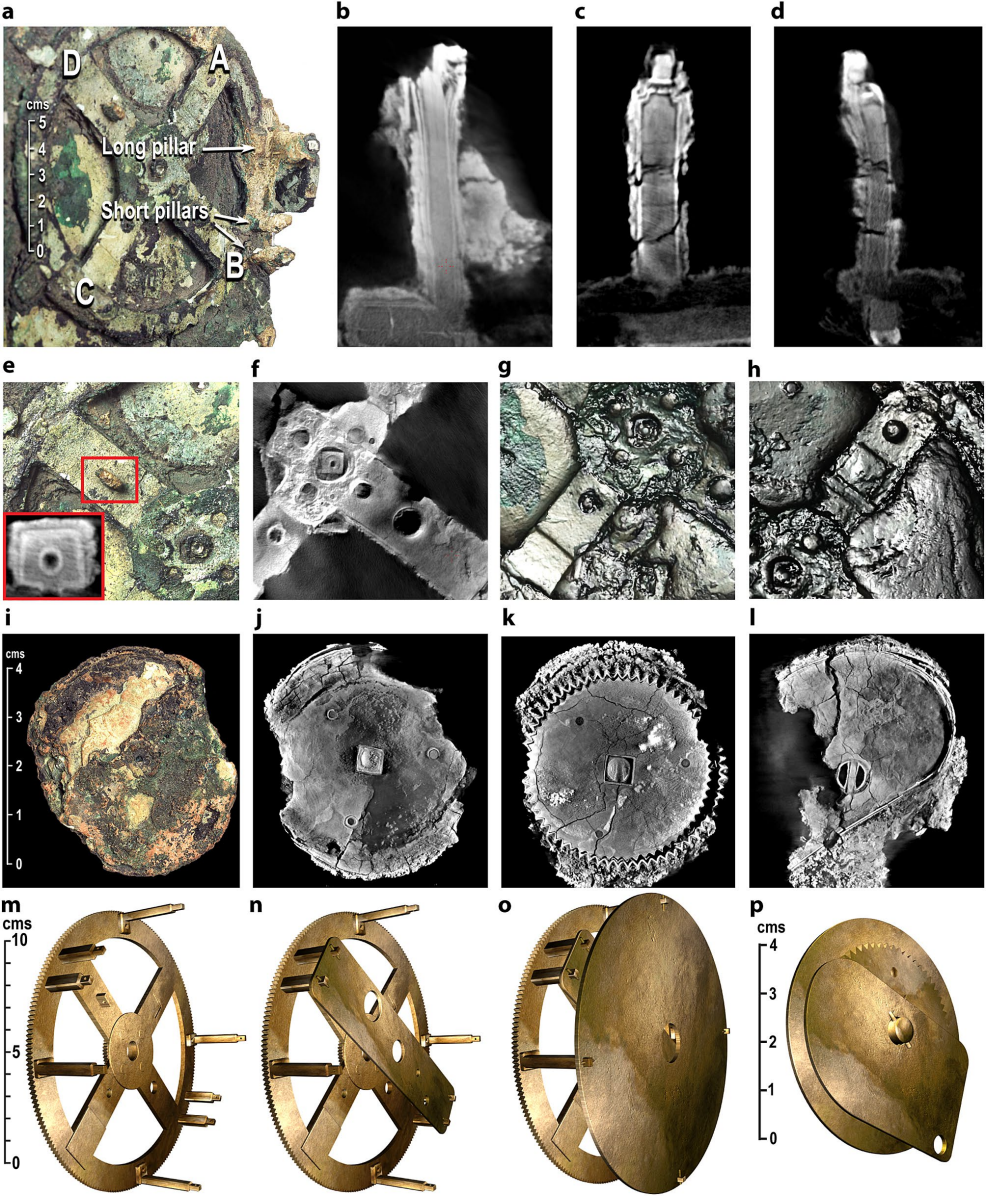
Evidence from Fragments A & D. Reconstructed plates and gear. (**a**) Photograph of Fragment A, showing pillars on the periphery of **b1** and features on Spokes A, B, C, D. (**b**) X-ray CT of long pillar. (**c**–**d**) X-ray CT of short pillars. (**b**–**d**) are from an improved X-ray volume^22^. All pillars have shoulders and pierced ends. (**e–h**) Photographs, PTMs, X-ray CT: features on Spokes D, B, C, A, including holes, circular depressions and flattened areas. In (**E**), the pierced block on Spoke D is highlighted in red, with inset showing X-ray CT slice through the block. (**i**-**l**) Photograph & X-ray CT of Fragment D, showing a *disk*, *gear* and *plate*. (**m**–**o**), Computer reconstruction, showing **b1**, **Strap** on the short pillars; **Circular Plate** (**CP**) on the long pillars. (**p**) Computer reconstruction of the features in Fragment D, which we reconstruct as the epicyclic components of a Venus mechanism.

The key question: could we match our theoretical mechanisms to the physical data? Fig. 4 shows some of the challenging evidence from Fragment A^7,22^ (Supplementary Figs. S9, S10, S11, S12) and Fragment D^1–3,7,9^ (Supplementary Figs. S13, S14). Any model must be consistent with these data (Supplementary Discussion S5) as well as conform to horological/engineering principles from the rest of the Mechanism (Supplementary Figs. S15, S16).

## Fragment A: essential framework for reconstruction

The **Main Drive Wheel**, **b1**, has four spokes with prominent holes, flattened areas and damaged pillars on its periphery (Fig. 4a-h, Supplementary Figs. S11, S12)—definitive evidence of a complex epicyclic system^1–3,9^. In the original Mechanism, there were four short and four long pillars with shoulders and holes for retaining pins, as shown in Fig. 4a-d by the X-ray CT evidence. These imply that the pillars carried plates: a rectangular plate on the short pillars, the ***Strap***, and a circular plate on the long pillars, the ***Circular Plate (CP)*** (Fig. 4m-o)^9^. This is the *essential framework* for any faithful reconstruction, with the four spokes advocating four different functions (Fig. 4e-h). First, we reconstruct the mechanisms between **b1** and the **Strap**.

## Fragment D: epicyclic components for venus

Figure 4i-l, Supplementary Fig. S13 show evidence of the crucial components in Fragment D. Earlier studies^2,4,5^ suggested that there are two gears in Fragment D, but this is an illusion created because the arbor has split^7,9^, as established in Supplementary Discussion S5 and Supplementary Fig. S13. The original tooth count can be reliably determined as **63** teeth, given all but three of the teeth survive^5,7,9^. The basic components of Fragment D are a *disk*, *gear* and *plate*, referred to here as the *D-plate*, and an *arbor* linking all three elements. The disk and gear are riveted together and have square holes at their centre matching squared sections on one end of the arbor. Inside the thickness of the gear, the arbor changes from square to round, where it emerges into the plate. With no space for any other bearing on this arbor, it must have pivoted in the D-plate, which also serves as a spacer to bring the epicyclic components to the correct level in the output hierarchy and as a bearing for an idler gear **26** in the Venus train.

No other surviving gear in the Mechanism has a disk attached. In an inferior planet mechanism, the pin-and-slotted follower requires a pin attached to the epicyclic gear but beyond its edge^3,21^: the attached disk is the right size to carry the pin at the correct distance from the centre to model the maximum elongation of Venus. It is surely the epicycle for Venus, as previously suggested^9^ and strongly reinforced here. The width of the *D-Plate* is commensurate with the width of the **Strap**, based on the separation of the short pillars.

## Mechanisms between b1 and the strap

**Figure 5:**
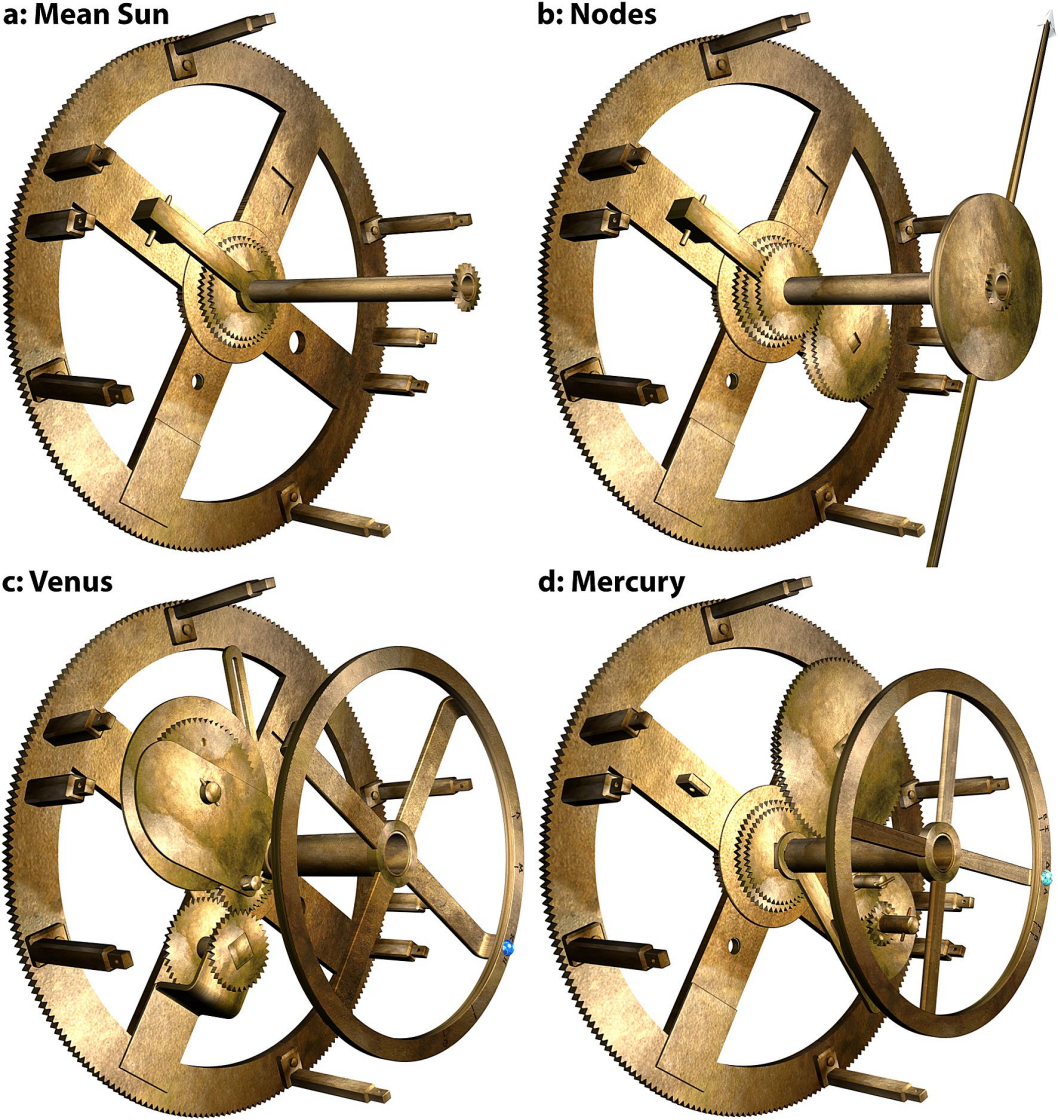
Mechanisms between b1 and the Strap. (**a**) **Mean sun**: Mean Sun bar attached to pierced block shown in Fig. 4e; tube and gear for input into Moon phase mechanism. (**b**) **Nodes**: Gears of Nodes mechanism, matching bearing in Fig. 4f—gear train **49** ~ **62** + **64** ~ **48**—with output tube and double-ended *Dragon Hand*. (**c**) **Venus**: Base gears of Venus mechanism match features in Fig. 4g. Gear train **51** ~ **44** + **34** ~ **26** ~ **63**—with components from Fragment D as reconstructed in Fig. 4p, plus output tube and Venus ring with lapis lazuli marker. The epicyclic gears **26** ~ **63** for Venus turn in the D-plate that is attached to the Strap (not shown). The end of the follower can be seen behind the disk. (**d**) **Mercury**: Base gears of Mercury mechanism match features in Fig. 4h. Gear train **51** ~ **72** + **89** ~ **40** ~ **20**—plus output tube and Mercury ring with turquoise marker. The epicyclic gears **40** ~ **20** for Mercury turn directly in the Strap (not shown). The follower can be seen behind the left-hand side of the Mercury ring.

Figure 5, Supplementary Fig. S22 show how the *mean Sun*, *nodes* and *inferior planets* are intricately constructed within the 15.0 mm space^9^ between **b1** and the **Strap** in nine closely-packed layers—matching the evidence and the layer density of the surviving gears (Supplementary Figs. S16, S20, S21, Supplementary Video S1). The mechanisms are interleaved so that their output tubes are nested in the CCO, with the lunar output on the central arbor.

The Moon phase device^10^ needs access to adjacent lunar and solar rotations, since the phase is the difference between these rotations: a ring output system appears to require calculating the *true Sun* twice^9^—once for input to the *Moon phase* and once for the *true Sun* ring, which is the third output in the ring system—so mechanically separated from the central lunar arbor. Here we solve this key problem with a *mean Sun* output, feeding into the Moon phase device as the first output tube adjacent to the central lunar arbor. **b1** carries the mean Sun rotation, but it is not possible to attach a mean Sun output at its centre because the central fixed gears prevent this: an attachment half-way along Spoke D is necessary to bridge the fixed gears. This is why the mean Sun output is attached via a bar to the previously-mysterious pierced block on Spoke D (Fig. 4e). This important idea enables a ring display for the Cosmos, with a single *true Sun* output for the solar ring. The small approximation inherent in using a *mean Sun* rather than a *true Sun* input to the Moon phase is negligible at the scale of the 6 mm diameter Moon phase sphere.

There are great advantages in a ring system of outputs as opposed to a pointer system. It coincides far better with the description of the output display in the BCI. It eliminates the severe parallax inherent in a pointer system with nine outputs. It greatly enhances the astronomical outputs, by enabling the synodic phases of the planets to be described by index-linked inscriptions, as we discuss later (Fig. 8). It leads to a robust and elegant display.

The close match between our proposed mechanisms and the data is shown in Fig. 4. The four spokes of **b1** suggest four different functions (Supplementary Fig. S12). The mean Sun and inferior planets take up three of these. What is the function of the prominent bearing on Spoke B (Fig. 4f)? Fig. 5b shows a solution: the bearing enables a four-gear epicyclic system that calculates the lunar nodes. Our proposed tooth counts for the gears and their modules (Supplementary Discussion S4) mean that the bearing is in exactly the right place on Spoke B. No other use has previously been found for this bearing.

The complex deductions that lead to unique reconstructions of the Venus & Mercury gear trains are described in Supplementary Discussion S5. We argue that Fragment D includes epicyclic components for Venus (Fig. 4, Supplementary Figs. S13, S14), that the gear trains follow our 5-gear design (Fig. 3) and that all must fit into the framework created by the pillars (Fig. 4). The prime factors in the period relations combined with the physical evidence then determine the gear trains (Fig. 3c, e, Fig. 4g, h, Fig. 5c, d). In particular, we show that the astronomical meaning of **63** is that it shares the prime numbers **3** × **7** with the period relation for Venus, (**289**, **462**) = (**17**^**2**^, **2** × **3** × **7** × **11**). The **Strap** is inclined to the spokes at just the correct angle of 11° to accommodate the epicyclic gears for Mercury and Venus—explaining the angle of the short pillars relative to **b1**. For the first time, the features on **b1** and the components of Fragment D are fully explained (Figs. 4, 5, Supplementary Fig. S21, Supplementary Discussion S5, Supplementary Video S1). We conclude that our Venus and Mercury gear trains are *strongly* indicated by the evidence.

## Mechanisms between the strap and the CP

**Figure 6:**
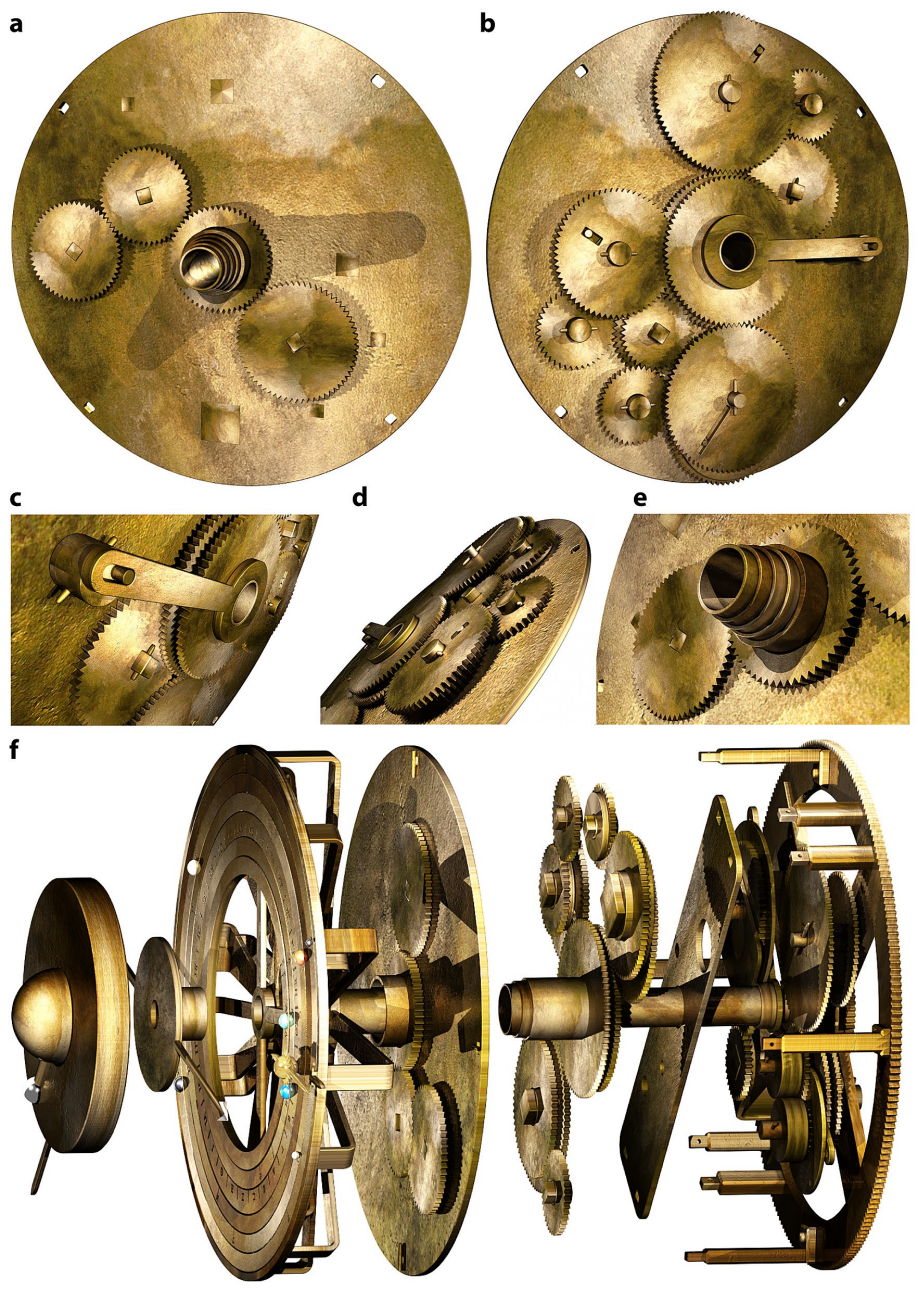
True Sun, Superior Planets and exploded Cosmos gearing. (**a**) The gears at the front of the **CP**. *Centre in (a):* fixed gear **56**, rivetted to a subsidiary plate (not seen). *Bottom right in (a):* **64**, shared between Mars and Jupiter; *Top left in (a):* **52**, shared between the *true Sun* and *Saturn*. *Left in (a):* **56** is the epicyclic gear for the true Sun gearing. (**b**) The mechanisms seen from the back of the **CP**. Clockwise from the top: *Saturn*, *true Sun*, *Mars*, *Jupiter*. (**c**) Close-up of true Sun mechanism. (**d**) Close-up of gears showing interleaved layers. (**e**) Close-up of output tubes. (**f**) Exploded model of Cosmos gearing. *From right to left*: **b1**, *mean Sun*, *Nodes*, *Mercury*, *Venus*; *true Sun* and *superior planets* gearing; **CP** and *shared gears*; *Ring Display*; *Dragon Hand*; *Moon position and phase mechanism*.

There is no surviving direct evidence for the gearing systems that calculated the true Sun and the superior planets. Inevitably this means choices, though the space available strongly limits these choices, since very compact systems are necessary to calculate the advanced period relations. Figure 6a-e show how most of the gears for the true Sun and superior planets are reconstructed within the 9.7 mm space between the **Strap** and the **CP**.

The initial gears for these systems are in front of the **CP** (Fig. 6a)—alleviating the space problem and creating a robust mechanical design with no need for brackets to support the mechanisms as in a previous model^9^. A fixed gear **56** at the centre engages with a compound epicyclic train on the **CP**, calculating the synodic rotation of the Sun/planet relative to the **CP**. The arbors of the three gears **52**, **56** and **64** go through the **CP** to drive the mechanisms at the back.

The mechanisms are arranged with their outputs in the CCO and are aligned on cardinal axes to facilitate calibration. The planetary periods and gear trains are listed in Fig. 3f and a schematic diagram is shown in Supplementary Fig. S23. Since the tooth counts must include the prime factors of the period relations, there are few viable options. The *true Sun* mechanism is a simple 3-gear system, previously proposed^3^ (Fig. 3b), calculating the ancient Greek epicyclic theory of the *true Sun*. It shares the fixed gear **56** with all the superior planets and it shares **52** with the Saturn mechanism. Hence it only needs one additional gear **56**. The superior planets, Mars, Jupiter and Saturn, are arranged clockwise from the top in Fig. 6b. All their mechanisms share a fixed gear **56** and follow the same economical 7-gear design shown in Fig. 3d.

The exploded diagram in Fig. 6f illustrates how all the Cosmos gearing fits together. We reconstruct 34 gears in front of **b1** for the Cosmos system. Extant systems account for 35 gears behind **b1** (Supplementary Table S8, Supplementary Fig. S20)^7^—making a total of 69 gears (Supplementary Videos S2, S3). The model follows all our *Reconstruction Principles* and matches *all* the evidence (Supplementary Discussion S1).

## Cosmos display

**Figure 7:**
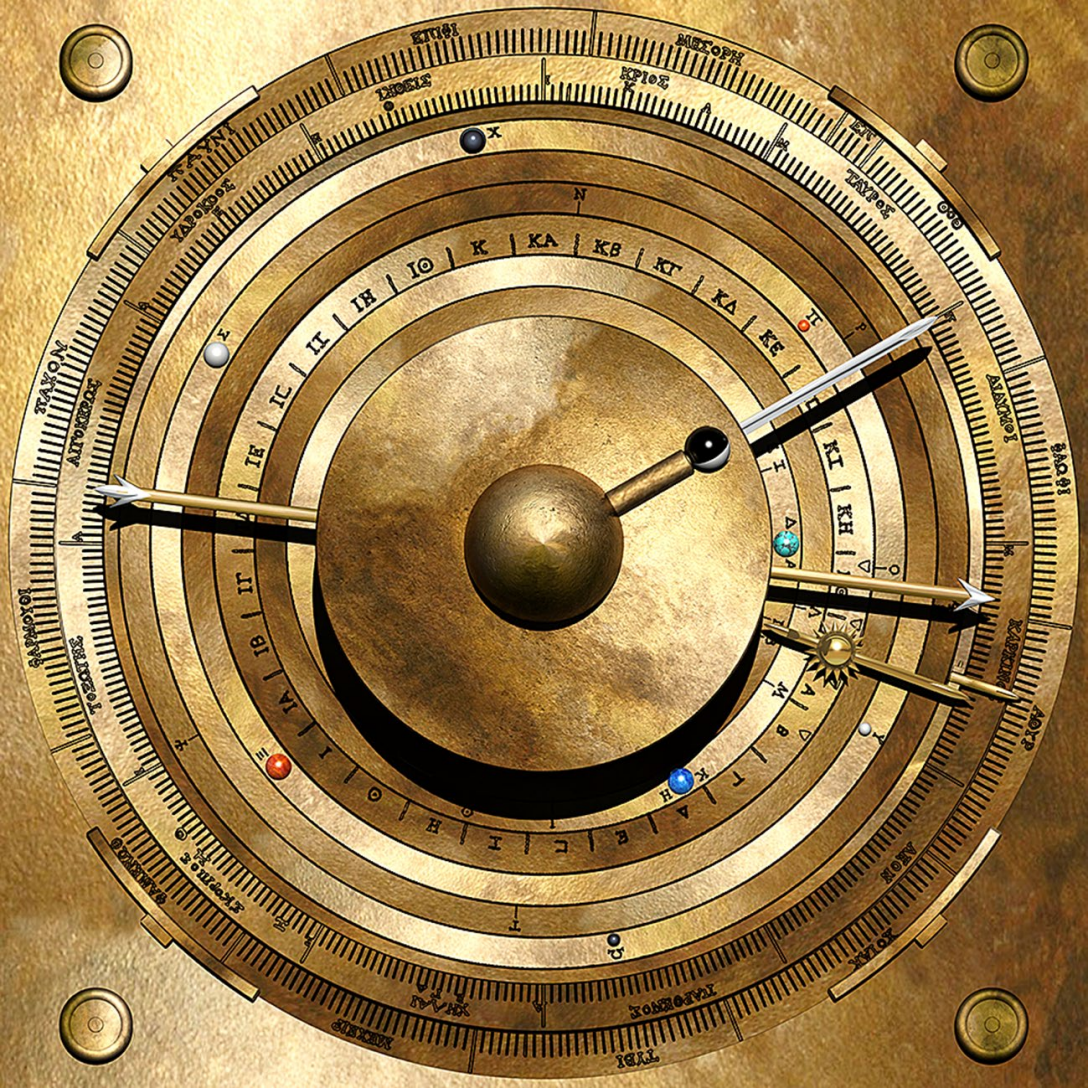
Computer model of the cosmos display. In the centre, the dome of the Earth, the phase of the Moon and its position in the Zodiac—then rings for *Mercury*, *Venus*, *true Sun*, *Mars*, *Jupiter*, *Saturn* and *Date*, with *“little sphere”* markers and smaller markers for oppositions. *Scale marks* and *index letters* for the synodic cycles of the planets are inscribed on the planet rings. Surrounding these, the *Zodiac* and the *Egyptian Calendar*^2^. The *true Sun* ring has a *“little golden sphere”* with *“pointer”*, as described in the BCI^9^. When the Moon and Sun pointers coincide, the Moon sphere shows black for New Moon; when the pointers are on opposite sides, the Moon sphere shows white for Full Moon^10^. The *Head* of the *Dragon Hand* shows the *ascending lunar node*; the *Tail* the *descending node*. Small triangles on the *true Sun* ring, near the pointer, show wider and narrower eclipse limits. Eclipses are possible if the Dragon Hand is within these limits. When the Moon pointer is before the *Head of the Dragon*, the Moon is *South* of the node; after, it is *North* of the node—conversely for the descending node. A *Date* pointer is attached to a narrow date ring, showing the date in the Egyptian calendar^2^.

Figure 7 combines our present discoveries into an elegant ancient Greek mechanical Cosmos at the front of the Antikythera Mechanism.

The planets are identified by semi-precious stones on planetary rings (Supplementary Figs. S3, S24, Supplementary Discussion S6, Supplementary Videos S1, S3). An *Age of the Moon* scale in days^3^ on the true Sun ring is read by the Moon pointer, echoing Cicero’s description of the Archimedes device (Supplementary Discussion S2), *“…it was actually true that the moon was always as many revolutions behind the sun on the bronze contrivance as would agree with the number of days it was behind it in the sky…”*.

The Dragon Hand indicates eclipses by its closeness to the true Sun pointer at New or Full Moon. *Closeness-to-node* defines the sophisticated eclipse prediction scheme on the Antikythera Mechanism^8,23^, with *symmetrical limits* for lunar eclipses; and *asymmetrical limits* for solar eclipses, according to whether the Moon is *North* or *South* of the node^8,23^. These wider and narrower limits are indicated by triangles on the true Sun ring. When the Dragon Hand is within the relevant limits, an eclipse prediction *glyph* can be found on the Saros Dial, with eclipse characteristics listed in the eclipse inscriptions^8,23,24^. If the Dragon Hand is within the wider limits, an *eclipse season*^23^ is in progress—occurring twice each *eclipse year*, shown by a full rotation of the Sun relative to the Dragon Hand. As a *User Manual*, the BCI (Fig. 1c) may have described these functions in the missing area above the planets (Fig. 1a).

## Indexing of synodic events to planetary rings

**Figure 8:**
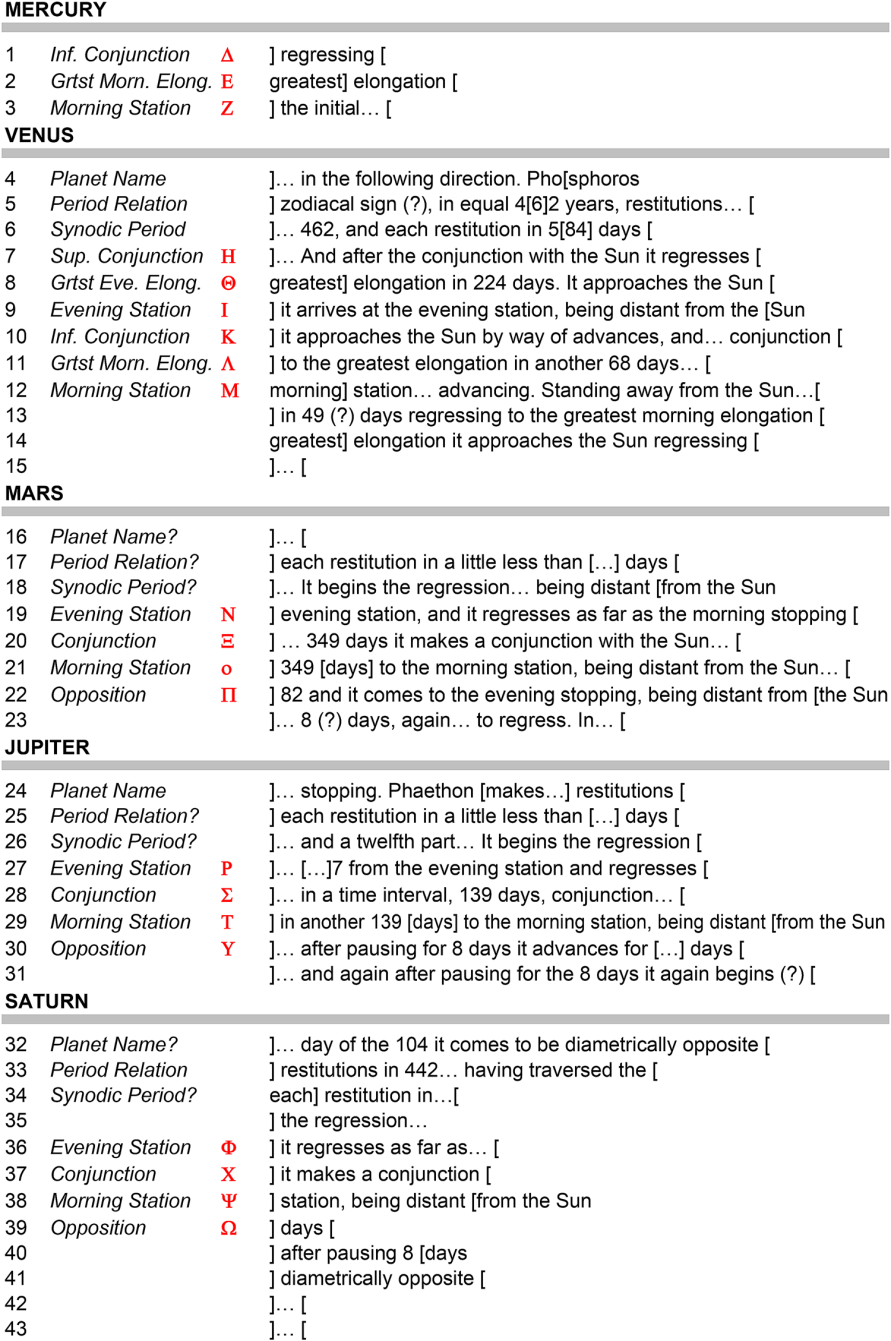
Hypothetical Index Letter Scheme for the **FCI.** The translation is from a previous publication^12^, where a transcription of the original Greek text can also be seen. The Index Letter scheme is in red. The whole scheme uses a single Greek alphabet from **A** to **Ω**, but the first few lines of Mercury are missing. The fragmentary data means that there are still many uncertainties in the lines of text.

As a rule, formulaic and repetitive inscriptions in the Antikythera Mechanism are indexed to their dials: for example, *Parapegma inscriptions* to the Zodiac Dial^1,2,7,25^ and *eclipse inscriptions* to the Saros Dial^8,12,23^. For each planet, its synodic events—*maximum elongations*, *stationary points*, *conjunctions* and *oppositions*—occur when the planet is at a characteristic angle from the Sun. By turning the Mechanism, we can note the Sun’s position on the planet’s ring for each synodic event (Fig. 7). We propose that the planetary rings were engraved with *scale marks* for these events read by the Sun pointer, with associated *index letters* beside the scale marks. Figure 8 shows how the index letters would have referenced the formulaic and repetitive events in the FCI.

Though this indexing scheme is not provable, as the beginning of the lines are lost (Fig. 1b, Supplementary Fig. S4), it makes such good sense in enhancing the astronomy on the Cosmos Display and it fits exactly with the line-by-line structure of the FCI. It is striking that the synodic events in the FCI are only those observable on the planetary rings: the customary appearances and disappearances of the planets are omitted, strengthening the indexing hypothesis. It is difficult to understand how the information in the FCI could have been easily accessed by the user without such an indexing system, which in turn justifies our ring system of outputs.

The FCI^9,12^ enumerates intervals in days between synodic events—probably calculated from epicyclic models, not observations, since the actual intervals are so variable (Fig. 1b). The embryonic trigonometry of the Hellenistic age^26^ would have made calculating these difficult. Here we propose that the Antikythera Mechanism itself calculated these synodic intervals by counting days on the *Calendar Dial* between synodic events indicated by the synodic scale marks on the planetary rings—entirely without trigonometry.

## Conclusions

Figure 7, Supplementary Figs. S24, S25, Supplementary Videos S1–S3 visualize our new model: the culmination of a substantial cross-disciplinary effort to elucidate the front of the Antikythera Mechanism. Previous research unlocked the ingenuity of the Back Dials, here we show the richness of the Cosmos at the front. The main structural features of our model are prescribed by the physical evidence, the prime factors of the restored planetary period relations and the ring description in the BCI. Hypothetical features greatly enhance and justify the Cosmos display: a *Dragon Hand* thematically linking the Front and Back Dials; and an *Index Letter Scheme* for the synodic events of the planets.

Because of the loss of evidence, we cannot claim that our model is a replica of the original, but our solution to this convoluted 3D puzzle draws powerful support from the logic of our model and its exact match to the surviving evidence. The Antikythera Mechanism was a computational instrument for mathematical astronomy, incorporating cycles from Babylonian astronomy and the Greek flair for geometry. It calculated the *ecliptic longitudes* of the Moon^7^, Sun^3^ and planets^1–3,9,11^; the *phase* of the Moon^10^; the *Age of the Moon*^10^; the *synodic phases* of the planets; the *excluded days* of the Metonic Calendar^8^; *eclipses*^7,8,23^*—possibilities*, *times*, *characteristics*, *years* and *seasons*; the *heliacal risings and settings* of prominent stars and constellations^1,2,7,25^; and the *Olympiad cycle*^8^—an ancient Greek astronomical compendium of staggering ambition. It is the first known device that mechanized the predictions of scientific theories and it could have automated many of the calculations needed for its own design (Supplementary Discussion S6)—the first steps to the mechanization of mathematics and science. Our work reveals the Antikythera Mechanism as a beautiful conception, translated by superb engineering into a device of genius. It challenges all our preconceptions about the technological capabilities of the ancient Greeks.

## Methods

Methods are incorporated into Supplementary Information.

## Supplementary Information

Supplementary Information 1.

Supplementary Information 2.

Supplementary Information 3.

Supplementary Information 4.

## Data Availability

The data that support the findings of this study are available from the corresponding authors upon reasonable request.
