# Author Correction: A Model of the Cosmos in the ancient Greek Antikythera Mechanism

**DOI:** 10.1038/s41598-021-96382-9

**Published:** 2021-08-24

**Authors:** Tony Freeth, David Higgon, Aris Dacanalis, Lindsay MacDonald, Myrto Georgakopoulou, Adam Wojcik

**Affiliations:** 1 Department of Mechanical Engineering, University College London (UCL), London, UK; 2 Department of Civil, Environmental and Geomatic Engineering, University College London (UCL), London, UK; 3 UCL Qatar, University College London (UCL), Doha, Qatar; 4 Science and Technology in Archaeology and Culture Research Center (STARC), The Cyprus Institute (CyI), Nicosia, Cyprus

Correction to: *Scientific Reports* https://doi.org/10.1038/s41598-021-84310-w, published online 12 March 2021

This original version of this Article contained repeated errors, where the Greek character ϒ (Upsilon) was incorrectly given as Ψ (Psi) and fractions were incorrectly shown as superscripts.

As a result, in the “Period relations and ancient Greek theories” section,

“Then came a notable discovery in 2016 in the FCI^12^: unexpected numbers ΨΞΒ (**462**) in the Venus section of the FCI and ΨMΒ (**442**) in the Saturn section, translating into highly accurate period relations: for Venus (**289**, **462**) and Saturn (**427**, **442**) (Fig. 1b, Supplementary Fig. S4)”

now reads:

“Then came a notable discovery in 2016 in the FCI^12^: unexpected numbers ϒΞΒ (**462**) in the Venus section of the FCI and ϒΜΒ (**442**) in the Saturn section, translating into highly accurate period relations: for Venus (**289**, **462**) and Saturn (**427**, **442**) (Fig. 1b, Supplementary Fig. S4)”

In the legend of Fig. 1,

“The numbers ΨΞΒ (462) in the Venus section and ΨMΒ (442) in the Saturn section are highlighted^12^ (Supplementary Fig. S4).”

now reads:

“The numbers ϒΞΒ (462) in the Venus section and ϒΜΒ (442) in the Saturn section are highlighted^12^ (Supplementary Fig. S4).”

In the “Theoretical mechanisms for our model” section,

“A rotation of **-**^**12/223**^ for the Line of Nodes, derived from the Metonic and Saros cycles^9^, could not be mechanized because of the large prime **223**. We show that a simpler ratio **-**^**5/93**^, with a more accurate period of 18.6 years^14^, can be calculated by a 4-gear epicyclic train (Fig. 3a, Supplementary Figs. S21, S22).”

now reads:

“A rotation of  $- \frac{12}{223}$ for the Line of Nodes, derived from the Metonic and Saros cycles^9^, could not be mechanized because of the large prime **223**. We show that a simpler ratio $- \frac{5}{93}$ , with a more accurate period of 18.6 years^14^, can be calculated by a 4-gear epicyclic train (Fig. 3a, Supplementary Figs. S21, S22).”

Lastly, in the Supplementary Information 4 file, the legend for Supplementary Fig. S4,

“The numbers ΨΞΒ (462) in Line 6 and ΨMΒ (442) in Line 33 are highlighted in red.”

now reads:

“The numbers ϒΞΒ (462) in Line 6 and ϒΜΒ (442) in Line 33 are highlighted in red.”

The original Supplementary Information 4 file is provided below.

The original Article and accompanying Supplementary Information 4 file have been corrected.

## Supplementary Information

Supplementary Information 4.

