# Supplementary material for: A Model of the Cosmos in the ancient Greek Antikythera Mechanism
Source: Sci Rep. 2021 Mar 12;11:5821. doi: 10.1038/s41598-021-84310-w (PMC7955085; doi:10.1038/s41598-021-84310-w)
Supplement: Supplementary file 4 — Supplementary Information 4. [file 41598_2021_84310_MOESM4_ESM.pdf]

Supplementary Information

A Model of the Cosmos in the ancient Greek Antikythera Mechanism

Tony Freeth*, David Higgon, Aris Dacanalis, Lindsay MacDonald,
Myrto Georgakopoulou, Adam Wojcik*

* Corresponding authors:
Tony Freeth: tony@images-first.com
Adam Wojcik: a.wojcik@ucl.ac.uk

Contents

Supplementary Information

Supplementary Discussion S1: The Physical Evidence
Supplementary Discussion S2: Texts & Inscriptions
Supplementary Discussion S3: Planetary Periods
Supplementary Discussion S4: Theoretical Mechanisms
Supplementary Discussion S5: Matching the Evidence
Supplementary Discussion S6: Reconstructing the Cosmos
Supplementary Figs. S1–S25.
Supplementary Tables S1–S9.
Image Credits & Software
References 1–26 & Supplementary References 27–61

Supplementary Video S1
Gear-by-gear computer reconstruction of the Cosmos of the Antikythera Mechanism.
Supplementary Video S2
A track round all the surviving and reconstructed gears of the Antikythera Mechanism.
Supplementary Video S3
An exploded model of the Antikythera Mechanism assembles itself into the complete device.

1. Supplementary Discussion S1: The Physical Evidence

1.1 Review of the Data

After its discovery in 1901 by Greek sponge divers, the recovered part of the Antikythera Mechanism was taken to the National Archaeological Museum in Athens, where it has remained ever since[2]. It is now split into 82 fragments. The major fragments are labelled A – G and the minor fragments 1 – 75[7]. These fragments are the primary evidence for the Mechanism and how it worked. Our project is to reconstruct the front gearing system and dials for the Cosmos. The main fragments that are relevant for this study are shown in Supplementary Fig. S1. The data come from two sources: direct evidence from Fragments A, C, D[9] (Supplementary Discussion S5) and indirect evidence from the fragments that reveal the inscriptions: *Front Cover*—Fragments G, 3, small fragments[7,12]; *Back Cover*—Fragments A, B, E,19, small fragments[9,13].

The evidence consists of high-resolution *still photographs*, surface imaging with *Polynomial Texture Mapping (PTM)* and high-resolution 3D X-rays with *Microfocus X-ray Computed Tomography (X-ray CT)* from new data gathered in 2005[7,8,22]. The X-ray CT showed the surviving internal structure of the device in three dimensions and produced a remarkable revelation: several thousand Greek text characters, which had not been seen since the machine was lost more than 2,000 years ago[7,8]. These cannot be read in 2D X-rays.

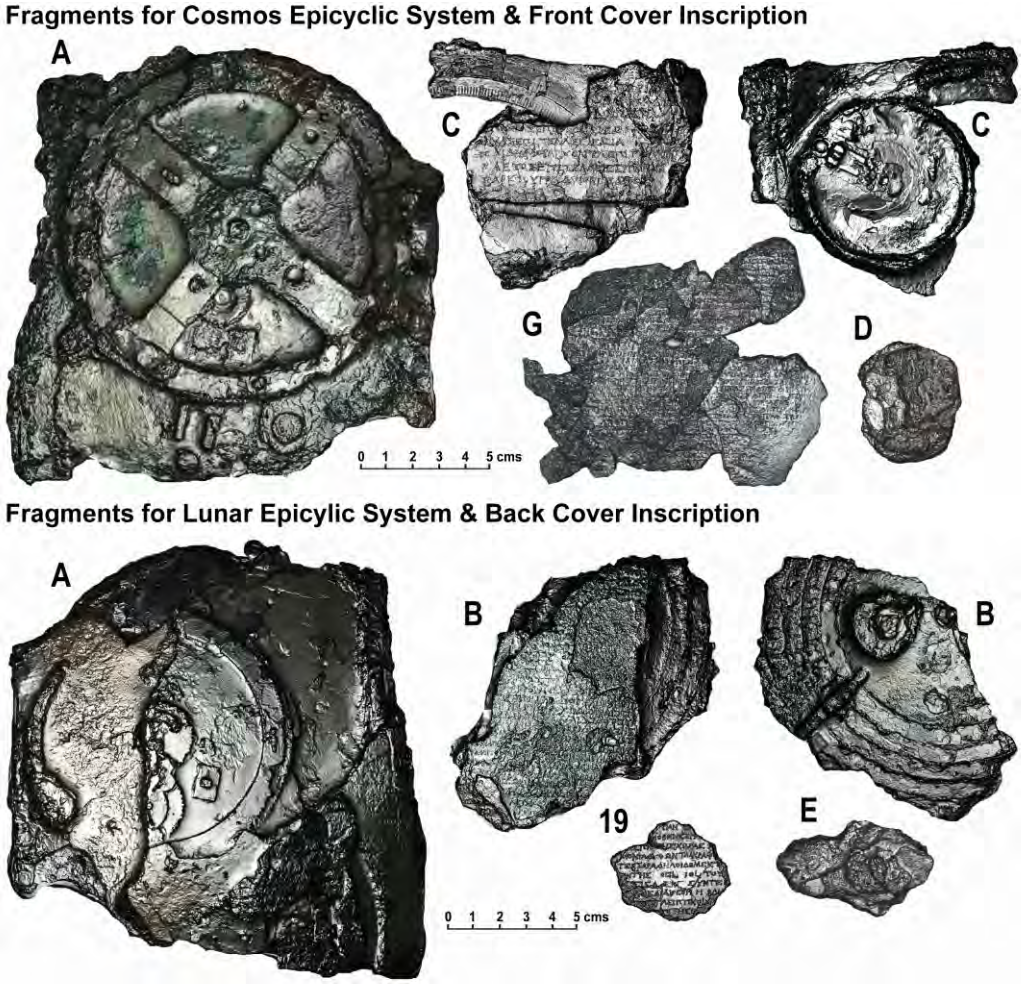

Supplementary Fig. S1. Fragments of the Antikythera Mechanism with evidence for this study. The data is PTM, using *specular enhancement*, from the 2005 investigations[7]. Fragments A and D are the physical evidence for the Cosmos epicyclic system[1,2,3,9]. Fragment G is the primary evidence of the Front Cover Inscription but numerous other small fragments contribute scraps of text to the interpretation[9,12]. The front of Fragment C shows the Zodiac and Calendar scales and has text for the Parapegma; the back includes the lunar phase mechanism[10]. The back of Fragment A shows the lunar epicyclic system. The Back Cover Inscription is evidenced by an amalgam of Fragments A, B, E and 19[13]. Fragment B is covered in mirror text with an impression of the original text, which is now lost.

These new data have led to a revolution in our understanding of the Antikythera Mechanism[7,8,9,23,27]. Amongst many discoveries, was an understanding of a remarkable previous observation[6] of a pin-and-slot device, which had not been understood. This astonishing discovery revealed a mechanical arrangement for tracking the variable motion of the Moon—the so-called *lunar anomaly*—using an ancient Greek epicyclic theory of lunar motion. It also explained the surprising identification of epicyclic gearing in the Antikythera Mechanism[1,2,6,7], which had previously been misinterpreted. Subsequent work has established beyond doubt that the planets were included in the Antikythera Mechanism[1,3,21,9] and very likely used similar mechanical devices to calculate their variable motions. The discovery of epicyclic gearing and the pin-and-slot device in the Antikythera Mechanism has triggered a fundamental reassessment of technology in the ancient world.

1.2 Reconstruction Principles

The Antikythera Mechanism's 82 surviving fragments constitute only about a third of the original device. What principles should we use in reconstructing the original machine? Some parts of the Antikythera Mechanism have now been reconstructed to general agreement, with evidence so persuasive that these aspects are unlikely ever to change: the gears that calculate the sidereal Metonic Cycle[2]; the synodic Metonic Cycle and its display on the five-turn spiral Metonic calendar[4]; the Saros Cycle that predicts eclipses on the four-turn spiral of the Lower Back Dial[7]; and the gear system that calculates the lunar anomaly and indicates this on the Zodiac Dial[7]. These involve hypothetical reconstructions of missing gear teeth and missing gears, but the contingent evidence is so strong that these restorations are *forced* by the data. Other parts of the reconstruction are more conjectural—for example, the *Kallippic Dial*[4,7], a subsidiary dial inside the Metonic calendar, where no physical evidence survives. It is justified because the 76-year Kallippic Cycle is explicitly described in the BCI[1,2] and it is the underlying cycle for the calendrical functions on the back of the Mechanism. It is also very easy to mechanize from existing gearing[4,7]. It is a highly plausible conjecture: not *forced* by the data but with *strong* evidence.

Our model is rooted in direct evidence of mechanical features in the fragments and indirect evidence from the numerous inscriptions, but we cannot rule out that future discoveries may lead to revisions. A key aspect of our reconstruction has been a very close examination of all the data: all previous models have largely chosen to ignore inconvenient data. With only a third of the original Mechanism surviving, we have followed four *Reconstruction Principles* in constructing our current model, with a fifth as work-in-progress for our ongoing and future research.

1. *Utilise and conform to all the surviving evidence.*
2. *Reflect the content of the inscriptions in the gears and dials.*
3. *Match to contemporary astronomy and technology.*
4. *Prefer simple and economical solutions.*
5. *Prove the feasibility of the model by making it with ancient techniques.*

This paper is essentially about our theoretical model of the Antikythera Mechanism, which conforms to *Reconstruction Principles 1-4*. Experimental archaeology, fulfilling *Reconstruction Principle 5*, will be a key focus for future research and publications by our team at UCL. We anticipate that this process will tell us much about ancient Greek technology, not just about the Antikythera Mechanism.

Some elements of our model are *forced* by the evidence; some are *strongly indicated*; and some are *hypothetical* additions. For each element, we describe the strength of data that validates it. The main structural features of our model are determined by the data: most of the choices are of less consequential aspects, such as the number of teeth of the idler gears. Other choices involve additional *hypothetical* elements, which fit the evidence and harmonize with its known functions, but which are not prescribed by it. These features respect all the evidence and often explain it. We have created the first model of the Antikythera Mechanism that matches the surviving evidence.

2. Supplementary Discussion S2: Texts & Inscriptions

2.1 Historical Texts

Historical texts give strong support for the idea that devices such as the Antikythera Mechanism existed in Hellenistic Greece and that they calculated planetary positions. In *Gears from the Greeks*[2], Derek de Solla Price cites two remarkable quotes from the Roman politician and lawyer, Marcus Tullius Cicero. These appear to describe similar instruments. In *De re publica I*, xiv (21–22) (54–51 BC), there is the following description of a machine made by Archimedes (c. 287–212 BC):

> *. . .on which were delineated the motions of the sun and moon and of those five stars which are called wanderers. . . (the five planets). . . Archimedes. . . had thought out a way to represent accurately by a single device for turning the globe those various and divergent movements with their different rates of speed. . . And when Gallus moved the globe, it was actually true that the moon was always as many revolutions behind the sun on the bronze contrivance as would agree with the number of days it was behind it in the sky. Thus, the same eclipse of the sun happened on the globe as it would actually happen.*

In *De natura deorum II*, xxxiv-xxxv (87-88) (45 BC), Cicero writes of a device made by Posidonios of Apamea (c. 135–51 BC), which is almost certainly a first-hand report, since Cicero knew Posidonios and attended his lectures in his Stoic school of philosophy in Rhodes. It describes:

> *...the orrery recently constructed by our friend Posidonios, which at each revolution reproduces the same motions of the sun, the moon and the five planets that take place in the heavens every day and night...*

Both descriptions sound very much like the Antikythera Mechanism and offer convincing support to the idea that such machines existed in Hellenistic times. It seems clear from the descriptions that the bronze devices tracked the positions of the planets in the Zodiac, but the descriptions are not specific enough to define their structures. Pappus of Alexandria (c. 290–350 AD) reports that Archimedes wrote a treatise *On Sphere Making*[28], which apparently described astronomical devices, perhaps like the Antikythera Mechanism. This treatise is now lost and there are no known copies.

2.2 Cosmological Order & Textual Analysis

Both the Back and Front Cover Inscriptions describe the planets in a particular *cosmological order*. Expanding this with Moon and Sun, this order is: *Moon*, *Mercury*, *Venus*, *Sun*, *Mars*, *Jupiter*, *Saturn*. This is directly relevant to the inscriptions and the structure of our model. In terms of a modern heliocentric view of the Solar System, the cosmological order of the visible planets is clear: *Mercury*, *Venus*, *Earth*, *Mars*, *Jupiter*, *Saturn* are ordered by their increasing distances from the Sun and their increasing orbital periods. For each planet, this distance varies because of their elliptical orbits, but these variations are small. The situation in a geocentric frame-of-reference is entirely different, since the distances from the Earth to the planets are very variable: for example, Mars is sometimes closer to Earth than Mercury. So, there is no natural order of the planets in terms of their distances from the Earth. In terms of orbital periods in a geocentric frame-of-reference, the mean motions of the superior planets—Mars, Jupiter, Saturn—are progressively slower against the stars, but the inferior planets—Mercury, Venus—have the same mean period of rotation as the Sun. It is not surprising therefore that there was much confusion in antiquity about the cosmological order. Some orders were mentioned in Babylonian and Egyptian astronomy, but they were primarily divinatory/astrological in nature and have little relevance for our study[14]. The oldest reference to a celestial planetary order in Greek astronomy comes down to us from Theon of Smyrna (1st century AD to 2nd century AD)[19], who suggested that the Pythagoreans (6th - 4th century BC) held as true the order: *Mercury*, *Venus*, *Sun*, *Mars*, *Jupiter*, *Saturn*. This is the order in the inscriptions on the Antikythera Mechanism. Plato (c. 426–348 BC) made two references to an order of the planets in his dialogues *Timaeus* and in *The Republic* (Book X)[29]: *Sun*, *Venus*, *Mercury*, *Mars*, *Jupiter*, *Saturn*. A scheme was developed by Aristotle (384 – 322 BC) and his work *On the Heavens* (350 BC)[30], but his ordering of the planets is not the

order on the Antikythera Mechanism. Influenced by Aristotle, the order of the superior planets as *Mars*, *Jupiter*, *Saturn* in terms of their progressively longer periods became increasingly universal, but there was still confusion about the inferior planets and the Sun. Geminos (1st century BC), whose astronomical exposition in his *Introduction to the Phenomena*[31] reflects much of the Antikythera Mechanism, asserted the same order as on the Antikythera Mechanism. We shall refer to this order as the *"customary cosmological order (CCO)"* in ancient Greece. It became the standard order in the medieval and renaissance periods (Supplementary Fig. S2a-c).

There is now a very large body of literature related to epigraphic analysis of the inscriptions on the Antikythera Mechanism, which is based on broad scholarship over more than a century of research. Whilst an appreciation of such linguistic analysis has been important to our understanding of the Antikythera Mechanism, detailed investigation extends beyond the scientific and engineering scope of this paper. For the interested reader, the most accessible overview of epigraphic research is given in a series of papers, published in the journal *Almagest*. The relevant papers for this study are included in the References[12,13,24,25]. These papers include comprehensive references to the wider body of literature.

2.3 The Back Cover Inscription

The Back Cover Inscription (BCI)[9,13] (Fig. 1c), shown in Supplementary Table S1, is part of the key evidence that the planets were included in the Antikythera Mechanism. Detailed analyses were published previously[9,13]. The exact interpretation informs us about the layout of the Cosmos Display. In the light of its crucial importance and the previous decipherment of the text and its translation, we perform our own analysis to confirm the form of the display.

Greek Text		English Translation
1	..[	*…*
2	ταύτην δ[	*this …*
3	δεῖ δ´ ὑπολαβ̣[εῖν	*One should understand…*
4	ὑπὸ δὲ τὸν τω.[	*Below the…*
5	δ.[-6-]οικα[	*…*
6	ε[-9-]ηι σπ̣[	*…*
7	[-10-] π̣ροσ̣[	*…*
8	ο̣[-10-]μθε.[	*…*
9	[-10-]ν ἥρμοσ[	*… fitted(?)…*
10	[-11-] ἐ̣π´ ἄκρου δ[	*… at the tip…*
11	[-11-].ωσμένων.[	*…*
12	[-12-]ε̣ μέλαν οτ.[	*… black…*
13	[-11-]...... λ̣ω̣ν γεγ[	*…*
14	[-10-].ε. δ´ ὑπολαβεῖ[ν	*… one should understand…*
15	[..]οθ̣ε.. τὸ σφαιρίον φερε.[	*… the little sphere travels…*
16	προέχον αὐτοῦ γνωμόνιον σ[	*… little pointer projecting from it…*
17	φερειῶν ἡ μὲν ἐχομένη τῶι τῆς [	*arcs, the one next to the… of the…*
18	τος, τὸ δὲ δι´ αὐτοῦ φερόμεν[ον	*Stilbon(?), and the… travelling through it*
19	τῆς Ἀφροδίτη<ς> Φωσφόρου...[	*of Aphrodite Phosphoros…*
20	τοῦ [Φω]σφόρου περιφέρειαν.[	*the periphery of Phosphoros…*
21	γνώμω[.] κεῖται χρυσοῦν σφαιρίον..[	*on the pointer lies a golden little sphere…*
22	Ἡλί̣[ου] ἀκτίν´, ὑπὲρ δὲ τὸν Ἥλιόν ἐστιν κυ[	*ray of the Sun, above the Sun is the circle(?)…*
23	[-3- το]ῦ Ἄρεως Πυρόεντος, τὸ δὲ διαπορε[υόμενον	*of Ares Pyroeis, and the… making its way through…*
24	[Διὸς Φα]έθοντος, τὸ δὲ διαπορευόμενο̣ν [	*of [Zeus] Phaethon, and the… making its way through…*
25	[νου Φα]ίνοντ̣ος κύκλος, τὸ δὲ σφαιρίον φλ̣[	*circle of [Kronos] Phainon, and the little sphere…*
26	[-7-]ε̣ρα δ̣ὲ τοῦ κόσμου κεῖται...[	*… of the cosmos lies…*
27	[-10-]μεν[.] στοιχεῖα παρακείμ[ενα	*… letters situated beside…*
28	[-12-] α̣ υτα ταῖς ἀσπιδ̣[ίσκαις	*… the little disks…*
29	[-12-] π̣ροειρημένα̣[	*… aforesaid…*
30	[-16-]α̣σ̣π̣[..].[	… disk(s)(?) …

Supplementary Table S1. Greek Text and English Translation of the Back Cover Inscription (BCI). Text and translation reproduced from a previous publication[13].

Our line-by-line analysis corroborates the previous assessment of this text[13], whilst highlighting individual words and phrases. We concentrate on Lines 15 – 30.

15 [..]οθ̣ε.. τὸ σφαιρίον φερε.[
Translation: *the little sphere [is carried]*

- σφαιρίον = "tiny sphere" note ending -ιον following 'sphere', sphere-ion= little sphere.

16 προέχον αὐτοῦ γνωμόνιον σ[

Translation: *Little ruler is protruding from it*

- gnomon = same word for sundial stick as well as being the word for a geometrical rule.
- gnomon-ion = tiny stick / tiny ruler (ending -ion indicating small size).
- It is not therefore necessary that it had a pointed end—a sundial stick and a ruler tend not to. There are other words that could have better described a pointy end; this can also imply a relatively thin (as in not wide) rectangular stick protruding outwards, sometimes interpreted as a stick with a truncated end instead of a symmetrical pointer[3].

17 φερειῶν ἡ μὲν ἐχομένη τῶι τῆς [

- "φερειων": this seems to be the last part of the composite word "περιφερειων" which is the plural of "periphery" i.e. what we interpret as the annuli. i.e. "peripheries".
- " ἡ μὲν" = " and the"; "ἐχομένη" = adjacent to

18 τος, τὸ δὲ δι' αὐτοῦ φερόμεν[ον

Translation: *…and the [little sphere] being borne through it...*

- The participle "being borne through" seems to agree with the word sphairion (the ending is missing); noun + participle are the subject of a verb which is missing in the gap.
- In Lines 18, 23, 24 and 25, "τὸ δὲ" was previously translated[13] simply as "and". The development of language from Ancient to Hellenistic Greek[32,33] suggests that it might be translated as "the other". In this case, these lines would describe a planet by its name but refer to *another* planet ("the other") in the description of its orbit. This would be a surprising structure for these formulaic inscriptions, but we leave the reader to decide this issue in the context of the cited references.
- From the point of view of our reconstruction, these considerations make no difference to our reconstruction: the essential role of the inscriptions for our reconstruction is to describe the planets as orbiting in rings, marked by "little spheres": this is realized in our model in the ring structure of the planetary display.

---

19 τῆς Ἀφροδίτη<ς> Φωσφόρου.. [

Translation: *of the Light-Bearing (in the sense of Light bringer) Aphrodite [Venus]*

- Φως-φόρος = light-bearer (φ=phi; phosphorus) - translated as "Lucifer" in Latin.

---

20 τοῦ [Φω]σφόρου περιφέρειαν [

- The periphery of Phosphorus [Venus].

---

21 γνώμω[ ] κεῖται χρυσοῦν σφαιρίον.. [

Translation: *…stick lies a little golden sphere*

- κείται = it lies from verb κείμαι = to lie, to be found somewhere.
- This passage describes a little golden sphere lying on another little stick—again, probably a pointer. It clearly refers to the Sun.
- σφαιρίον—see note under Line 15.

---

22 Ἡλί[ου] ἀκτίν', ὑπὲρ δὲ τὸν Ἥλιόν ἐστιν κυ[

Translation: *ray of the Sun; beyond the Sun lies a circle*

- Ἡλί[ου] ἀκτίν'. Ἡλιος=Helios= Sun & ἀκτίν' α = ray (the word is the mathematical term for a radius).
- The term «ἀκτίν» is in the accusative singular case. The passage may describe a sunray of some sort or it may refer to a pointer, carrying the Sun.
- 'Υπερ': υπερ = hyper = over, beyond, above. This is indicating that the description of the circle that follows is progressing outwards, beyond the Sun. i.e.

there lies a circle beyond where the Sun can be seen. So, up to now we have a series of circles, then the Sun and then more circles (circular paths). This suggests the cosmological order where the Sun is above Mercury and Venus.
- Κύκλος = cycle = circle. It is now clear that the periphery described above is a circle, strongly suggesting that the description is of concentric circular bands.

---

23 [-3- το]ῦ Ἄρεως Πυρόεντος, τὸ δὲ διαπορε[υόμενον
Translation: *…of Fiery Mars, and travelling through it*
- For interpretation of "τὸ δὲ", please see the note under Line 18.
- Διαπορευόμενον = δια + πορεύομαι. δια = through, πορεύομαι = to walk, to move.
- This word and the pattern before indicates another little sphere "moving through" a path. "It", the object of the verb, probably refers to the 'circle' in the previous sentence; therefore, 22 combined with 23 seems to say that the circle beyond the Sun is that of the Fiery Mars, and that there is a little sphere moving through that circle. It is now becoming apparent that these circles are not literally geometrical circles; the word is used in the sense of a circular path; perhaps more akin to an annulus rather than a circle of no width.

---

24 [Διὸς Φα]έθοντος, τὸ δὲ διαπορευόμενο̣ν [
Translation: *…of Brilliant Zeus, and travelling through it*
- For interpretation of "τὸ δὲ", please see the note under Line 18.

---

25 [νου Φα]ίνοντ̣ος κύκλος, τὸ δὲ σφαιρίον φλ̣[
Translation: *…circle [of Saturn], and the little sphere*
- For interpretation of "τὸ δὲ", please see the note under Line 18.
- The repetitive nature of this passage and its similarity to the previous ones clearly make up a pattern: the description progresses outwards from the centre, identifying the "circles", or pathways, belonging to each planet, followed by a description of the sphere of that planet—along the lines of: "beyond the Sun lies the circle of Mars, with the little sphere being [colour/material], beyond that lies the circle of Jupiter, with the little sphere being [colour/material], beyond that…

---

26 [-7-]α̣ρα δ̣ὲ τοῦ κόσμου κεῖται ̣ ̣ ̣[
Translation: *next to the [world] lies*
Note that there has been a minor revision of the text here: the uncertain ε̣ has been revised to an uncertain α̣ to match the word Παρά = next to.
- Κόσμος = world, universe related to the verb 'κοσμῶ' meaning to adorn. Κόσμημα = jewellery 'item of beauty to be adorned'.
- Παρά = next to.
- This sentence wraps up the description of what appears to have been nothing other than… the world, the Cosmos. This clearly indicates the intention of whatever is being described to be interpreted as a description of the Cosmos, i.e. the depiction of a Cosmological model with the Earth in the centre, with the five planets and the Sun and the Moon moving in concentric circular paths around it.
- Whatever follows this sentence is a description of something which lies beyond the planetarium. It probably refers to the Zodiac annulus or the Egyptian Calendar ring. One would expect the Zodiac to be part of the Cosmos, since the sphere of the fixed stars is the outmost sphere in ancient Greek cosmology[30].

---

27 [-10-]μεν[̣] στοιχεῖα παρακείμ[ενα
Translation: *elements lying next to*
- Στοιχεία = elements; the derivative verb στοιχῶ = to arrange in a straight line, to line up.
- Word στοιχεία used in the sense of "elements", "data", "information" which is presented for the viewer. *Lying next* implies there is information lying next to something else – we interpret this as a reference to text lined up next to the Cosmos display, potentially index letters and their descriptions.

---

28 [-12-] α̣ υτα ταῖς ἀσπιδ̣[ίσκαις
Translation: *…the little shields*

- ʼΑσπιδίσκος = small shield. Not simply a disc; a shield implying a curved surface. Δισκος on its own would imply a flat disc; ἀσπιδίσκος implies a curved surface like a shield; the term is in plural form implying a number of such little shields. From the position of this term in the text, it seems likely that these would be outside the planetarium display.
- We do not understand the "little shields" in Lines 28 and 30. One possibility is that they refer to the thumb catches[34] that held the front dial plate to the Mechanism as clearly seen in the X-ray CT of Fragment C, though it is unclear why these minor mechanical details should be included here. Another possibility is that the phrase "little shields" (ἀσπιδ̣[ίσκαις) refers to a set of stars in the constellation of Argo—the "little shields" protecting the argonauts from arrows on the ship Argo ("little shield" stars are referenced in Ptolemy's description of Argo[35] (*Almagest*, p.389). Again, it is difficult to see why these stars in a southern constellation would be included here in what could be a description of the Zodiac constellations and major stars that are included in the Parapegma.

---

29 [-12-] πρ̣οειρημένα̣[
Translation: *aforementioned*

---

30 [-16-]α̣σ̣π[̣ ̣] ̣[
Translation: *… disk(s)(?) …*

- Ασπ potential element of ἀσπιδίσκος – potential mention of the little shields, possibly the thumb catches, or maybe a removable cover that "shields" the inner workings of the Mechanism.

2.3.1 Structure of the BCI & Implications for the *Cosmos Display*

Supplementary Table S2 shows the structure of the BCI. For the planets, it follows a pattern: *"…beyond the Sun lies the circle of Mars, with the little sphere… beyond that lies the circle of Jupiter, with the little sphere… beyond that…"* —all in the CCO. The Greek text uses a number of similar words—arcs, periphery, circle. We do not distinguish between them, since they all appear to refer to the ring structure of the Cosmos display. We use the term "indicator" to describe the *"little Spheres"* that mark the astronomical bodies and the term *"pointer"* to refer to pointers that indicate ecliptic longitudes on the Zodiac Dial.

Body	Secular Name	Divine Ruler	Circle or Ring	Indicator	Pointer	Evidence or BCI Lines
Moon	*Selēnē*	*Selēnē*		*black-white sphere*	*Definitely*	*Fragment C*
Nodes	*Syndesmoi*				*Double*	*Hypothetical*
Mercury	Stilbōn	Hermēs	belt	little sphere	No mention	18
Venus	Phosphōrus	Aphroditē	periphery		No mention	19, 20
Sun	Hēlios	Hēlios		little golden sphere, ray	Pointer	21, 22
Mars	Puroeis	Arēs	circle	sphere	No mention	23
Jupiter	Phaethōn	Zeus		little sphere	No mention	24
Saturn	Phainōn	Kronos	circle	little sphere	No mention	25

Supplementary Table S2. Structure of Planetary Inscriptions. Features are highlighted in red where there is evidence—though there are many uncertainties. Two conjectured lines of inscription (in italics) about the Moon and Nodes are included. Evidence in Fragment C shows that the Moon was represented at the front, with its phases shown on a small black-white or semi-silvered sphere[10], as in many later astronomical devices[20]. The other lines show indications of the planets from the BCI.

As shown in Supplementary Fig. S2, the same structure is found in geocentric visions of the Cosmos throughout the Medieval and Renaissance periods, which derived so much from ancient Greece. In Supplementary Fig. S2A, note the use of *Faeton* for Jupiter—the ancient Greek secular name. In Supplementary Fig. S2a-c planets are nested in rings in the CCO.

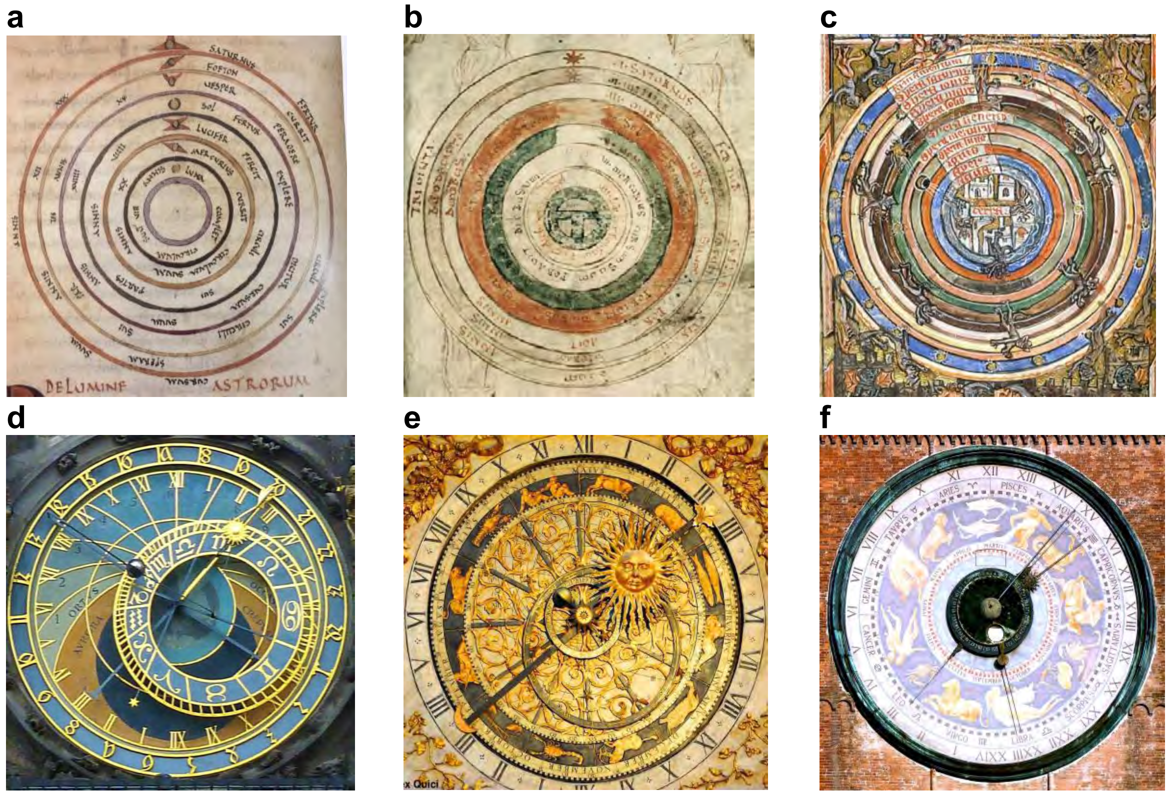

Supplementary Fig. S2. Customary Ancient Greek Cosmos from Mediaeval & Renaissance sources. (**a**) 10th century copy of a 7th century book *On the Nature of Things* by Isidore of Seville. (**b**) Bede, *De natura rerum*, 11th century AD. (**c**) *The Spheres Between Heaven and Hell, with Fallen Angels Becoming Devils*, Neville of Hornby Book of Hours, 14th century AD. (**d**) Prague, Czech Republic, 1410. (**e**) Clock by Guillaume Nourrisson, St Jean, Lyon, France, 1661. (**f**) Clock by Francesco and Giovan Battista Divizioli, 1583-88, in the Torrazzo of Cremona, Italy. *For image credits, see information at the end of this document.*

It is this image that we propose was reproduced in the Cosmos display on the Antikythera Mechanism[9]. In our model, we have not included "*Fallen Angels Becoming Devils*" (Supplementary Fig. S2f), because the Antikythera Mechanism is a secular device, which is entirely devoted to scientific astronomy!

In Supplementary Fig. S2d-f there are images of astronomical clocks from the 15th-17th centuries[20]. The dates of the clocks are given but all have been extensively restored and changed over the centuries. All these clocks show the Sun with rays and the phase and position of the Moon. The clock in Supplementary Fig. S2f has a Dragon Hand. The clockfaces are surrounded by a zodiac and a calendar—just as in the Antikythera Mechanism. None of them includes the planets—maybe because the simple ancient Greek epicyclic theories of the planets were known by this era to be inaccurate. A noteworthy clock was the 14th century AD *Planetarium of Giovanni de' Dondi*[36], which incorporated Ptolemy's advanced theories of the planets, but this had separate faces for each planet: it would have been very difficult to incorporate Ptolemy's theories in a coaxial output system.

In the period 1905-06, a pioneering researcher, Rehm[1], proposed a ring system for the planets (Supplementary Fig. S17), wrongly suggesting that the five rings in Fragment B were for the five planets. We now know that these rings were a fixed part of the Metonic Calendar on the Upper Back Dial[4,7]. A model of the Antikythera Mechanism (Price & Deroski) in the National Archaeological Museum in Athens has concentric rings for the Sun and Moon. In another publication[9], the authors (Freeth & Jones) wanted to create a ring display for the planets because of the description in the BCI but were unable to find a satisfactory mechanical solution for the Moon phase device (Supplementary Discussion S6).

It is possible that the Moon and the Nodes were described in the missing lines of text above the surviving inscriptions in the BCI. From the inscriptions we can infer the type of display indicator for each of the astronomical bodies—Supplementary Table S2. The Earth is at the centre. The Moon display is witnessed by Fragment C, which shows that the Moon's phases were represented by a little black-and-white (or semi-silvered) sphere of about 6 mm diameter[10]. The Moon's position in the Zodiac was surely indicated by a pointer attached to

the Moon phase cylinder, though this part of the cylinder is missing—possibly torn off by the pointer during the shipwreck. We have no evidence for the *Line of Nodes*, so this option is included in Supplementary Table S2 in italics and represented by a hypothetical double-ended *Dragon Hand*, indicating the *ascending* and *descending* nodes—often found in mediaeval astronomical clocks[20] (Supplementary Fig. S2f). The term *Dragon Hand* possibly originated in ancient myths that solar eclipses occur when a dragon devours the Sun, such as the Tinnīn of the Arabs, the Djawzahr of the Persians and the demon Rãhu of Hindu mythology[37]. We interpret the inscriptional data as follows: the *Moon* and *Sun* are indicated by *"little spheres"* with *"pointers"* attached—the Sun being portrayed with a *"ray"*, as in many mediaeval astronomical clocks (Supplementary Fig. S2d-f); the Moon's phase is indicated and the true Sun pointer is attached to a ring; the planets are shown in rings; the *Line of Nodes of the Moon* by a hypothetical *Dragon Hand*[20]; the *planets* are indicated by "*little spheres"* attached to concentric rings in the CCO.

2.3.2 Markers for the astronomical bodies

An astrological tradition used *"magic stones"* as markers for the planets, as in a 2nd/3rd century AD papyrus[38,9] about an "Astrologer's Board", where the astrologer lays out stones:

> *...a voice comes to you speaking. Let the stars be set upon the board in accordance with [their] nature except for the Sun and Moon. And let the Sun be golden, the Moon silver, Kronos of obsidian, Ares of reddish onyx, Aphrodite lapis lazuli veined with gold, Hermes turquoise; let Zeus be of (whitish?) stone, crystalline (?)...*

a **b** **c**

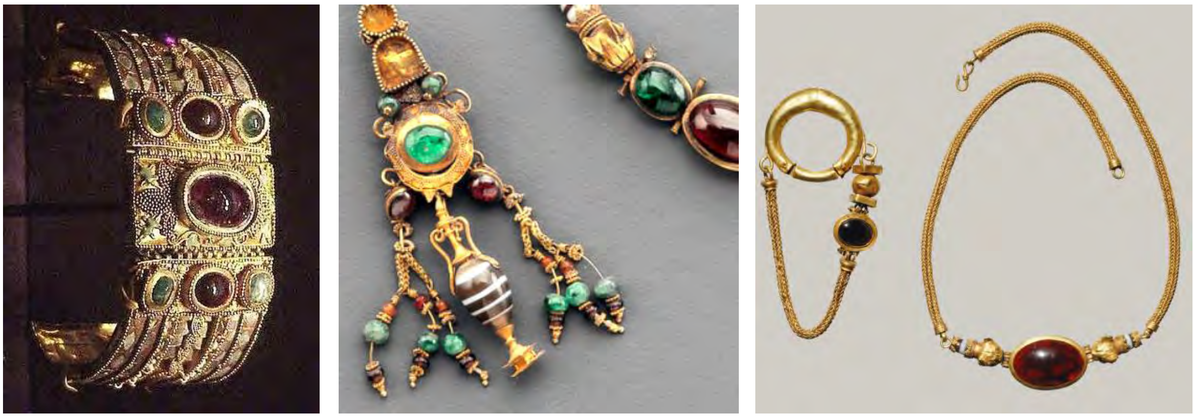

Supplementary Fig. S3. Examples of Hellenistic jewellery, inlaid with semi-precious stones. (**a**) Bracelets from the Olbia Treasure, 2nd/1st century BC. (**b**) Jewelled Gold Necklace and Earrings, 1st Century BC. (**c**) Necklace and Earrings, Late Hellenistic, 1st century BC. *For image credits, see information at the end of this document.*

In Supplementary Fig. S3 there are illustrations of a well-established practice in Hellenistic jewellery of using semi-precious stones with a smooth polished *cabochon* cut and an oval or circular shape—ideal as markers for the planets. The small-scale workmanship inspires confidence that the skills existed in ancient Greece to make the Antikythera Mechanism. Our model of the Cosmos display, using semi-precious stones as markers, can be seen in Fig. 7, Supplementary Figs. S24, 25 and Supplementary Videos S1, S3.

2.4 The Front Cover Inscription (FCI)

2.4.1 Planetary Motions

The inscriptions leave no doubt that the planets were included in the Antikythera Mechanism. The modern term *planet* derives from the ancient Greek term '*πλανήτης ἀστέρας*' (planḗtēs astéras) or *wandering star*. The movements of the planets fascinated the ancient astronomers because of their irregularity. We think of the Solar System as being Sun-centred, with planets rotating in nearly circular orbits. For the ancient astronomers the motions of the planets were Earth-centred, and they were a deep problem because they displayed retrograde motion, whereby they sometimes reverse direction relative to the stars—referred to in astronomy as *anomaly*. Mostly the planets move along the ecliptic in *prograde* motion but sometimes they come to a halt, a *stationary point*, and then reverse direction in *retrograde* motion before resuming their prograde motion. We think of prograde

motion as being represented by anticlockwise rotation: this is how it appears when viewed from north of the ecliptic. However, ancient astronomers tended to view the system from the south celestial pole, as in stereographic projection, which underlies ancient treatises and astrolabes[20,42]. This is the clockwise convention found on the Antikythera Mechanism.

a

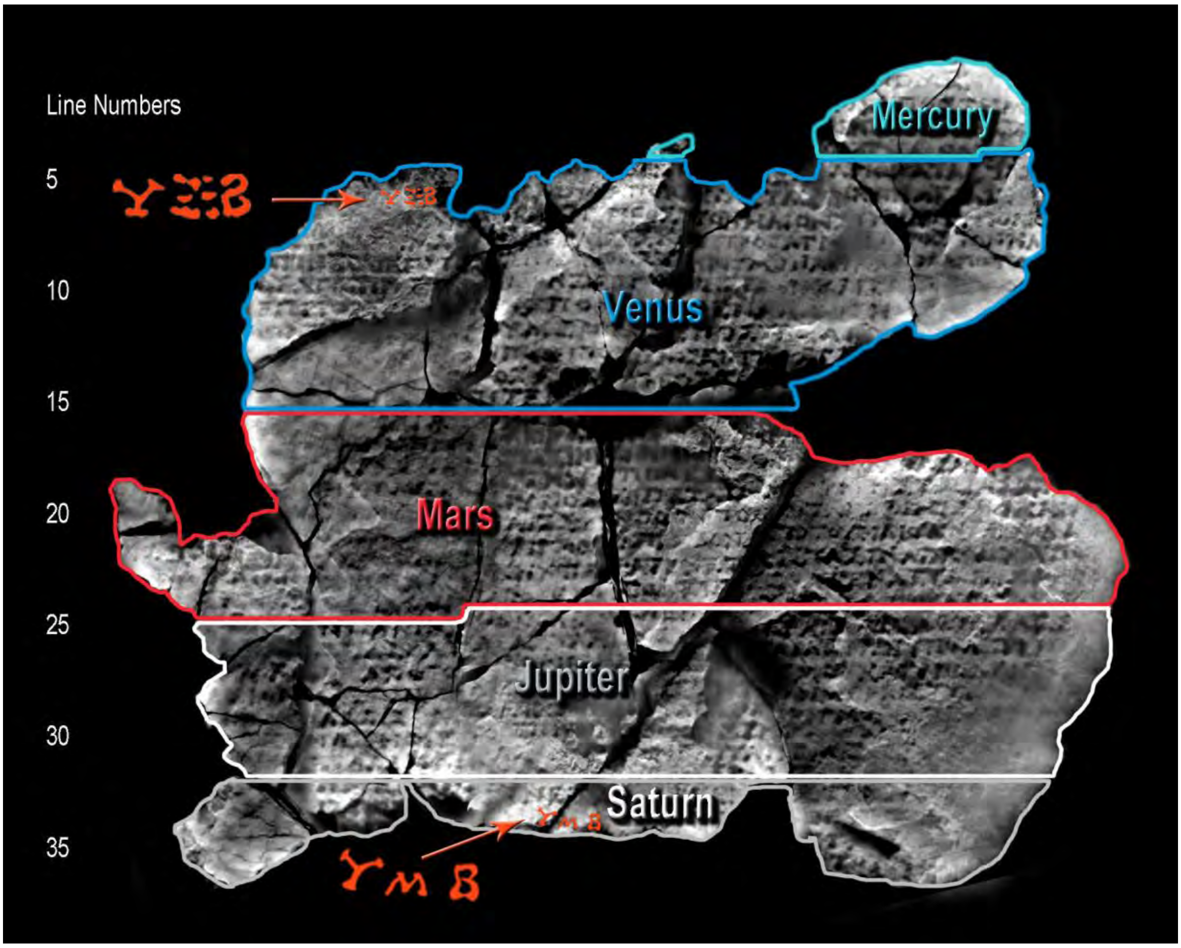

b

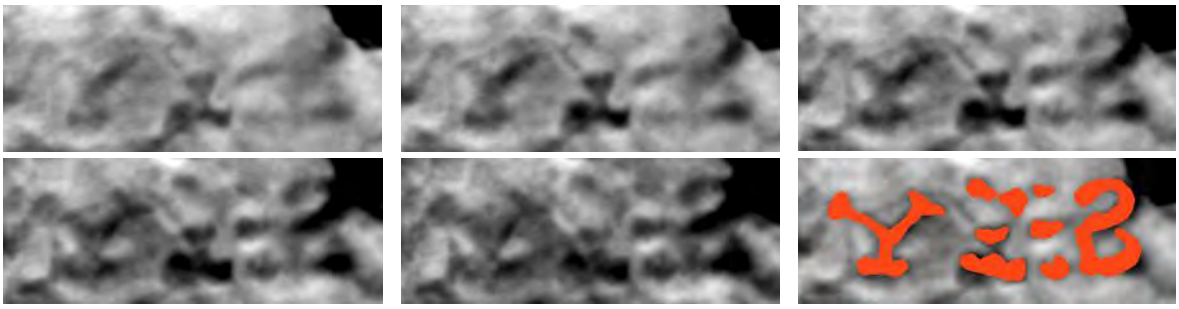

c

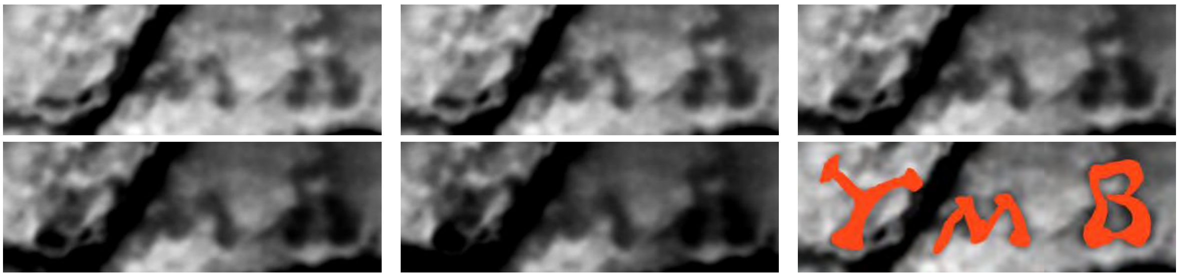

Supplementary Fig. S4. X-ray CT slices showing text in Fragment G. The numbers Υ Ξ Β (462) in Line 6 and Υ Μ Β (442) in Line 33 are highlighted in red.

(**a**) Composite of X-ray CT slices of Fragment G, divided into different regions for each planet, though the exact divisions are uncertain.

(**b**) Close-up X-ray CT slices, spaced at 7 microns, of Line 6 of the FCI**.**

(**c**) Close-up X-ray CT slices, spaced at 3 microns, of Line 33 of the FCI.

In Supplementary Fig. S4 there is X-ray CT data of the *Front Cover Inscription* (FCI), which survives in Fragment G and other small fragments[12]: it describes parameters of the synodic cycles of the planets. This crucial evidence lists both the main events of the synodic cycles and the time intervals in days between them in the CCO—the same order as the planets on the Back Cover Inscription.

Throughout these discussions, **blue numbers** and **gears** will incorporate the factors of the number of **synodic cycles** and **red numbers** and **gears** the factors of the number of **years**. A recent publication[12] has identified the surprising numbers **462** in the Venus section and **442** in the Saturn section. The publication that revealed these numbers did not show the evidence for their existence nor did it draw meaningful conclusions about mechanical structure from the newly-discovered period relations: the authors wrote that this was outside the scope of their study. In our view, these numbers are so important that we must show the evidence, so that the numbers cannot be in doubt. In Supplementary Fig. S4b, the characters ΥΞΒ can be clearly seen in the X-ray CT slices. In Supplementary Fig. S4c, the characters ΥΜΒ can also be seen: the Υ is harder to decipher than the other characters but it is definite when examined carefully. ΥΞΒ does not occur in ancient Greek as part of a longer word[39]. There are Greek words that include ΥΜΒ, but the adjacent areas of the plate show that ΥΜΒ is not part of a longer word in this context. So, these characters must be numerals: ΥΞΒ means **462**; ΥΜΒ means **442**. ΥΞΒ is in the section of the Front Cover Inscription that describes Venus and ΥΜΒ in the section for Saturn. The newly-discovered periods translate into *period relations* as follows[12]: Venus: (**289**, **462**), Saturn: (**427**, **442**). These periods are very surprising in their exquisite accuracy. They clearly have implications for the design of the planetary mechanisms. The authors of the paper that revealed these new periods[12] write about Venus:

> *The ratio 462 : 289 factorizes as (2 x 3 x 7 x 11) : (17 x 17), so it can be represented by a gear train with reasonable tooth counts, e.g. (66 : 51) x (63 : 51).*

They do not describe how this might have worked for Venus in the Antikythera Mechanism. If only four gears are used, then the epicyclic gear would turn in the wrong direction, so an idler gear is necessary. We therefore reject this inappropriate gear train: it does not turn the epicycle in the right direction, and it does not fit the evidence from Fragment A (Supplementary Fig. S9). Gear trains that actually work in the context of the Antikythera Mechanism are discussed in Supplementary Discussion S4**.** Despite the tooth count **63** being identified for a possible gear, the authors surprisingly make no link with the **63**-tooth gear in Fragment D (Supplementary Fig. S13, Supplementary Discussion S5).

Accuracy of planetary mechanisms

In a pioneering paper[3], Michael Wright describes how the gear ratios for the planetary mechanisms were chosen to be accurate to 1° in 500 years. The author used compound gearing to model these highly accurate periods and subsequently[40] reinforced his arguments for the inclusion of compound gear trains, incorporating the newly-discovered period relation for Venus into his model[16]. A previous publication[21] showed that simple epicyclic theories of the planets are inherently very inaccurate. For example, Mars will be wrong by as much as 38° within two years. The reason is that the retrogrades of the epicyclic theories are mechanically equal, whereas the actual retrogrades of the planets are of unequal size and are unevenly distributed through the Zodiac, because the planetary orbits are elliptical not circular and are not in the plane of the ecliptic[41,42]. It seemed unnecessary to pursue the long-term accuracy of compound mechanisms: simple Babylonian *Goal Year* period relations and the consequent simple gearing should surely be accurate enough. The new periods for Venus and Saturn show that the ancient Greeks did not take this view and used highly accurate period relations. A consequence is that the ancient Greeks were almost certainly unaware of the highly *inaccurate* nature of their epicyclic theories when the Antikythera Mechanism was made. It has been written[14,42] that Hipparchos (c.190 –120 BC) did not propose a theory of planetary motion, maybe because he was aware of the inaccuracies of epicyclic theories but was unable to discover a better theory. Perhaps Hipparchos was influenced by the evident inaccuracies of mechanisms such as the Antikythera Mechanism.

The new period relations must surely mean that they were intended to be used in the gearing of the Mechanism, rather than just being astronomical information in the inscriptions[16]. Since compound gear trains have already been discovered in the Antikythera Mechanism—for example, for calculating the sidereal Metonic Cycle[2]—their use for planetary mechanisms is completely justified by our *Reconstruction Principles*.

2.4.2 Front Cover

It has been suggested that the Front and Back Covers might have been hinged to the Mechanism[2]. The inscriptions on the Front Cover are on the outside, so they could not be viewed at the same time as the Front Dials, if the Front Cover were hinged. Since we propose that the inscriptions in the FCI were indexed to the planetary rings (Fig. 8), the user would need to be able to see both Front Cover and Cosmos Display at the same time. We therefore suggest that the Front Cover was on a separate plate that could be detached from the Mechanism. The Front Cover both protected the front display and gave additional information about the synodic cycles of the planets. There has been a debate about whether the FCI covered the whole front face of the Mechanism or only covered the Cosmos Display[12]. The *Parapegma* on the Front Plate above and below the dials[8,12] is a detailed description of stellar events, such as the heliacal risings and settings of prominent stars. Together, the BCI, the FCI and the Parapegma comprise a full description of the ancient Greek geocentric Cosmos. It is difficult to see what else might have been included that needed the extra space of a full-length cover: our Front Cover only covers the Front Dials.

a b c

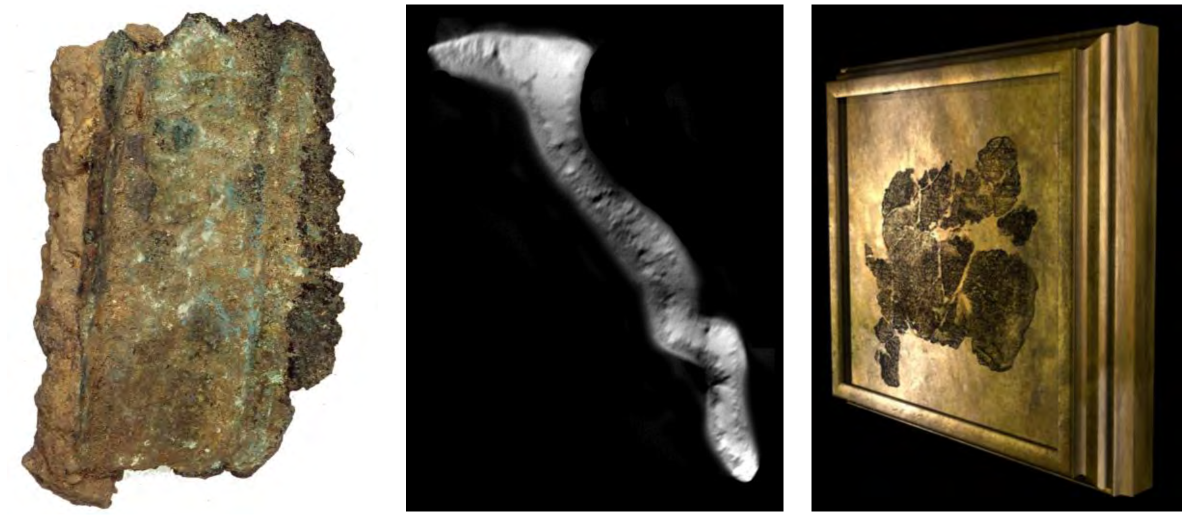

Supplementary Fig. S5. Fragment 3. (**a**) Photograph. (**b**) X-ray CT cross-section. (**c**) Reconstruction as Front Cover moulding.

In Fragment 3, there is a small section of decorative moulding 38.3 mm high, whose function has always been unclear. It is difficult to understand its role as part of the case. We suggest that it might have framed the Front Cover. If the Front Cover only covered the display and was framed by this moulding, then the size of the Front Cover plate would be 128.8 mm square. Fragment G + Fragment 29 (Fig 1b) are together 93.3 mm high for 36 lines of text. This leaves enough space for an additional 12 lines of text and a small margin. To complete the planetary inscriptions, Mercury needs 6 more lines at the beginning and Saturn runs to Line 43. A total of 49 lines would therefore be needed for the synodic cycles of the planets, taking up around 127 mm and fitting neatly on the Front Cover plate. If this is correct, then it does not appear that there was an introductory section in the FCI about the Moon and Sun as previously suggested[12]: this information was probably given in the BCI. We therefore propose that the FCI was entirely formulaic information about the synodic cycles of the planets and we adopt this hypothesis for our model.

3. Supplementary Discussion S3: Planetary Periods

3.1 Babylonian Astronomy

Mesopotamia (Gr; μεσο+ποταμοί = *'land between rivers'*) is the region between the rivers Tigris and Euphrates, mostly situated in modern Iraq. One of the earliest forms of writing, *cuneiform*, originated in this region. Cuneiform means 'wedge-shaped' and was written on soft clay tablets by the indentations of a wedge-shaped reed stylus. Tablets have been recovered in their hundreds of thousands, now scattered in museums around the world. A large number concern astronomy: many decades of study have revealed an extraordinary mathematical astronomy, predicting astronomical events—largely in the service of omen telling[15,43,44,45,46,47,48,49,50,51,52]. A key aspect was the prediction of planetary positions by developing *period relations*. The earliest known is the description of the synodic cycles of Venus in the 2nd millennium BC *Venus tablet of Ammisaduqa* (British Museum)[42]—with the following "ideal" periods: East, 8 months 5 days visibility, 3 months invisibility; West, 8 months 5 days visibility, 7 days invisibility; for a total of 19 months and 17 days. Using 29.53 days for a lunar month, this totals 578 days—quite accurate compared to the modern value of 584 days.

Supplementary Tables S3, S4 show period relations and errors from Babylonian astronomy.

**MERCURY**	*Attested Error*	GYT/ACT	*Sources*	*Date Range*	*References*
**(19,6)**	+10 days		BM45728	6th/5th Century BC	Kugler, pp. 45-48 | Hunger, p. 203 | Rochberg p.271
**(22,7)**					Swerdlow p. 191
**(41,13)**	-3 days	GYT	BM41004	End 4th century BC	Neugebauer-Sachs, p. 206 | Hunger, p.205
**(104 ,33)**					Brown, p.178
**(145, 46)**	-1 day	GYT	BM41004	End 4th century BC	Neugebauer-Sachs, p. 206 | Hunger, p.205
		ACT	ACT 800	Undated	ACT Vol. II p.287, pp.362-4
**(394, 125)**	Same day		BM41004	End 4th century BC	Neugebauer-Sachs, p. 206 | Hunger, p.205
**(684, 217)**		ACT	ACT 300b	301/300 to -293/292	ACT Vol. II p.288, 316-7
**(1223, 388)**		ACT	ACT 300a	-307/306 to -289/288	ACT Vol. II p.288, 316-7
**(1513, 480)**		ACT	ACT 300	-193/192 to -168/167	ACT Vol. II pp.288, 317-8
**(2673, 848)**		ACT	ACT 301	-178/177 to -158/157	ACT Vol. II p.288, pp.318-21
		ACT	ACT 302	-145/144 to -122/121	ACT Vol. II p.288, pp.321-4
		ACT	ACT 303	-128/27 to -125/124	ACT Vol. II p.288, p.324
		ACT	ACT303a	-97/96 to -91/90	ACT Vol. II p.288, p.324
		ACT	ACT303b	-95/94 to -82/81	ACT Vol. II p.288, pp.324-5
		ACT	ACT 304	-87/86 to -85/84	ACT Vol. II p.288, p.325
**VENUS**	*Attested Error*	GYT/ACT	*Sources*	*Date Range*	*References*
**(5, 8)**	- 4 days	GYT	BM45728	6th/5th Century BC	Kugler, pp. 45-8 | Rochberg p.271
	- 2;30 days	GYT	BM41004	End 4th century BC	Brown p. 175
	- 4°	GYT	BM41004	End 4th century BC	Neugebauer-Sachs, p. 206 | Hunger, p.204
	- 4 days	GYT	BM41004	End 4th century BC	Neugebauer-Sachs, p. 206 | Hunger, p.204
	- 4;10 tithis	ACT	ACT 400	-200/199 to -176/175	ACT Vol II, p.301, 329
	- 2;30° or -2;40°	ACT	ACT 410	-75/74 to -52/51	ACT Vol II, p.301, pp.330-1
**(720, 1151)**		ACT	ACT 400	-200/199 to -176/175	ACT Vol II, p.300, 329 | Van der Waerden, p.111
**MARS**	*Attested Error*	GYT/ACT	*Sources*	*Date Range*	*References*
**(15, 32)**	- 5 days		BM41004	End 4th century BC	Neugebauer-Sachs, p. 206 | Hunger, p.204
**(22, 47)**	+ 12 days	GYT	BM45728	6th/5th Century BC	Kugler, p. 45-8 | Rochberg p.271 | Brown pp.176-7
	+ 4 days	GYT	BM41004	End 4th century BC	Neugebauer-Sachs, p. 206 | Hunger, p.204
		ACT	ACT 811	Undated	ACT Vol II, p.302, pp.380-1, p.390
**(30,64)**	+4 days	GYT	BM41004	End 4th century BC	Neugebauer-Sachs, p. 206 | Hunger, p.204
**(37,79)**		GYT	ACT811	Undated	Louvre AO 6455 (TU 11)
		ACT	ACT501	-188/187 to -109/108	ACT Vol II, p.302, p.335
		ACT	ACT811	Undated	ACT Vol II, p.302, pp.380-1
		ACT	ACT811a	Undated	ACT Vol II, p.302, p.390
**(59, 126)**	Same day		BM41004	End 4th century BC	Neugebauer-Sachs, p. 206 | Hunger, p.204
**(133, 284)**		ACT	ACT500	-222/221 to -180/179	ACT Vol II, p.302, p.335 | Brown p.178
		ACT	ACT504	Undated	ACT Vol II, p.302, pp.337-8 | Brown p.178

Table S3. Babylonian records of planetary periods for Mercury, Venus & Mars.

**JUPITER**	*Attested Error*	GYT/ACT	*Sources*	*Date Range*	*References*
**(11, 12)**	- 7 days		BM41004	End 4th century BC	Neugebauer-Sachs, p. 206 | Hunger, p.204
	+ 5°		BM41004	End 4th century BC	Neugebauer-Sachs, p. 206 | Hunger, p.204
	+ 6°		BM34560	Undated	Neugebauer-Sachs, p. 207 | Hunger, p.204
		ACT	ACT 813	Undated	ACT VII, pp.403-422
	- 4;10 degrees	ACT	ACT 814	Undated	ACT VII, p404, 422
**(65, 71)**	Same day		BM41004	End 4th century BC	Neugebauer-Sachs, p. 206 | Hunger, p.204
	- 5°		BM34560	Undated	Neugebauer-Sachs, p. 207 | Hunger, p.205
		ACT	ACT 813	Undated	ACT VII, p.307, pp.403-420
		ACT	ACT 814	Undated	ACT VII, p.307, pp.422-4
**(76,83)**	-7 days	GYT	BM41004	End 4th century BC	Neugebauer-Sachs, p. 206 | Hunger, p.204
		ACT	ACT 813	Undated	ACT VII, p.307, pp.403-420
		ACT	ACT 814	Undated	ACT VII, p.307, pp.422-4
**(87,95)**		ACT	ACT 813	Undated	ACT VII, p.307, pp.403-420
		ACT	ACT 814	Undated	ACT VII, p.307, pp.422-4
**(239,261)**		ACT	ACT 813	Undated	ACT VII, p.307, pp.403-420
		ACT	ACT 814	Undated	ACT VII, p.307, pp.422-4
**(293,320)**		ACT	ACT 813	Undated	ACT VII, p.307, pp.403-420
		ACT	ACT 814	Undated	ACT VII, p.307, pp.422-4
**(315, 344)**	- 3 days, - 4;33°		SP II 985	Undated	Kugler pp. 48-50
**(391,427)**		ACT	ACT 600	-198/197 to -138/137	ACT VII, p.307, p.339
		ACT	ACT 610	-169/168 to -116/115	ACT VII, p.309, p.343
		ACT	ACT 622	-121/120 to -80/79	ACT VII, p. 310, p.349
		ACT	ACT 640	-180/179 to -150/149	ACT VII, p.311, p. 352
**SATURN**	*Attested Error*	GYT/ACT	*Sources*	*Date Range*	*References*
**(29, 30)**	+ 9 days	GYT	BM41004	End 4th century B.C.	Neugebauer-Sachs, p. 206 | Hunger, p.204
	+ 7;20°	GYT	BM41004	End 4th century B.C.	Neugebauer-Sachs, p. 206 | Hunger, p.204
**(57, 59)**	Same day		BM45728	6th/5th Century BC	Kugler, pp. 45-8| Rochberg p.271
	- 6 days	GYT	BM41004	End 4th century BC	Neugebauer-Sachs, p. 206 | Hunger, p.204
	- 5 days		BM34560	Undated	Neugebauer-Sachs, p. 207 | Hunger, p.205
**(142, 147)**	Same day		BM41004	End 4th century BC	Neugebauer-Sachs, p. 206 | Hunger, p.204
**(256, 265)**		ACT	ACT 801	Undated	ACT Vol II, pp.313-4, pp.366-371
		ACT	ACT 802	Undated	ACT Vol II, pp.313-4, pp.371-3
			BM36753	Undated	ACT Vol II, p.315
			BM37336	Undated	ACT Vol II, p.315
		ACT	Ephemerides	-225/224 to -177/76	ACT Vol II, 313-4, pp. 357-61
				-203/202 to -193/2 & others down to - 59/58 BC	

Table S4. Babylonian records of planetary periods for Jupiter & Saturn.

For Supplementary Tables S3, S4, errors are expressed in days, degrees or tithis (1/30th of a mean synodic month). Numbers separated by a semi-colon are in sexagesimal notation[14]. A negative error means that the number of synodic cycles is achieved in less than the indicated number of years. *GYT* stands for *Goal-Year Texts*; *ACT* stands for *Astronomical Cuneiform Texts; BM* stands for *British Museum; SP II* stands for the second collection of cuneiform tablets acquired by the *British Museum* from *Spartali & Co.* in the period 1879-80. The references used are: Neugebauer[15], Hunger[44], Kugler[45], Brown[46], Neugebauer-Sachs[47], Rochberg[49], Swerdlow[50].

In Babylonian astronomy, there were a number of different texts related to observational astronomy and to mathematical procedures. The *Goal-Year Texts* (GYT) and the *Astronomical Cuneiform Texts* (ACT) are particularly relevant to the planets[14,15]: they include information about planetary periods and their errors, as shown in Supplementary Tables S3, S4. The Babylonian schemes for predicting the positions of the planets were tied to the period relations and these ensured the long-term accuracy of the predictions, provided the errors were small. We will be particularly concerned with errors in planetary periods.

3.2 Ancient Greek Epicyclic Theories of the Planets

The ancient Babylonian astronomical theories were primarily based on arithmetic procedures for making predictions. The ancient Greeks developed a strong tradition of geometrical approaches to the Cosmos[14]. However, this simple distinction has been called into question by more recent research, particularly on the Oxyrhynchus Papyri[53], which reveals Greek astronomers using arithmetical methods borrowed from the Babylonians and by research on Babylonian astronomy that shows the use of sophisticated geometry to track the motion of Jupiter[54].

Because the anomalies meant that the planets could not be explained by simple uniform circular motion round the Earth, Apollonios of Perga (late 3rd to 2nd century BC) and contemporaries developed a beautiful theory to explain the motions of the planets as the sum of two uniform circular motions—the so-called *deferent and epicycle* models[14], as shown in Supplementary Fig. S6.

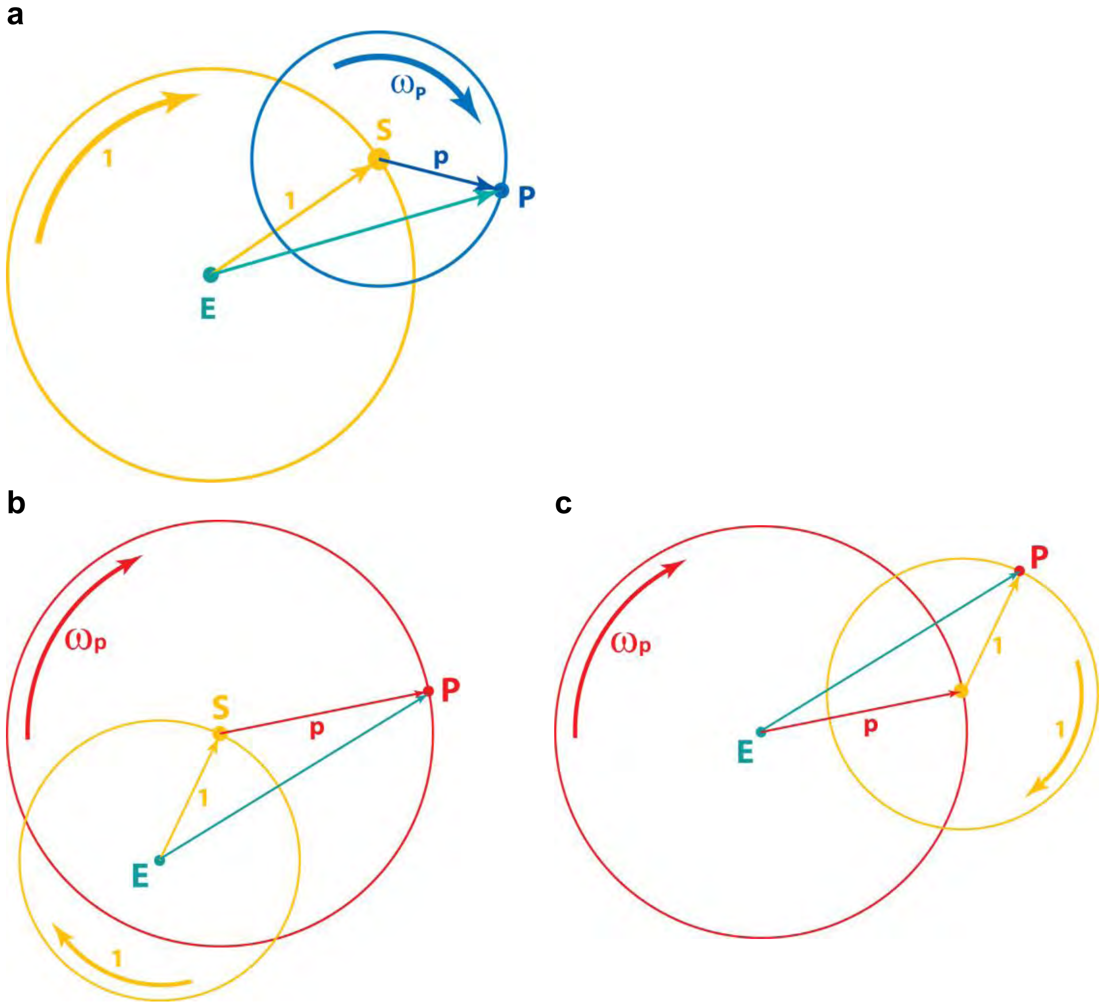

d

Planet *P*	Distance *p, AU*	Rot. Period *pP, years*	Rot. Period *days*	Rot. Rate *ωP rots per year*	Syn. Period *years*	Syn. Period *days*
Mercury	0.39	0.241	88	4.149	0.317	116
Venus	0.72	0.615	225	1.626	1.599	584
Earth	1.00	1.000	365			
Mars	1.52	1.881	687	0.532	2.135	780
Jupiter	5.20	11.863	4,333	0.084	1.092	399
Saturn	9.58	29.447	10,756	0.034	1.035	378

Supplementary Fig. S6. Epicyclic Theories of the planets & Planet Parameters.

(**a**) Inferior planet: Sun rotation as deferent, planet rotation as epicycle. ω_P is the rotation of the epicycle *in the real world* (as opposed to *relative to the deferent*).

(**b**) Superior planet: Sun rotation as deferent, planet rotation as epicycle. ω_P is the rotation of the epicycle in the real world.

(**c**) Superior planet: Planet rotation as deferent, Sun rotation as epicycle. ω_P is the rotation of the deferent in the real world.1 is the rotation of the epicycle in the real world. In modern terms, this model simply reverses the order of addition of the vectors.

(**d**) Planet parameters from modern theory.

To aid in the visualization of the epicyclic models, previous research[21] considered a *Simplified Solar System* where all the planets move around the Sun uniformly in circles. From a geocentric viewpoint, the ancient Greek epicyclic theories exactly model the motions of the planets in this *Simplified Solar System*.

3.2.1 Inferior Planet parameters

For each planet, three parameters determine the simple epicyclic theories: the rate of rotation of the deferent, the rate of rotation of the epicycle relative to the deferent and the radius of the epicycle (r), relative to the radius of the deferent (R). The rates of rotation can be calculated from the period relation. We shall assume that R = 1.

Suppose (Π, Y) is a period relation for an inferior planet P. The rate of rotation of the deferent is 1 rotation per year. The rate of rotation of the epicycle in the real world is ω_p. By previous theory[21], for an inferior planet:

$$\omega_p = (\Pi + Y)/Y$$

We shall refer to the deferent as S, since it rotates at the rate of the mean Sun. By the additive law of relative rotations (Supplementary Discussion S4), the rotation of the epicycle relative to the deferent, $\omega_{p\,|\,S}$ is:

$\omega_p = \omega_{p\,|\,S} + \omega_S = \omega_{p\,|\,S} + 1$. In other words, $\omega_{p\,|\,S} = \omega_p - 1 = \Pi / Y$

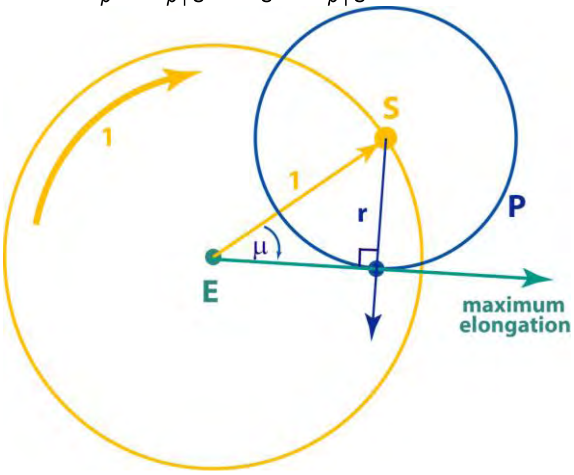

Supplementary Fig. S7. Inferior planet: determination of the size of the epicycle, r, from observations

Determination of the size of the epicycle for an inferior planet is easy. We assume that R = 1. From observations, we can determine the planet's maximum elongation from the Sun, μ: for example, about 46° for Venus. Then r = sin μ = 0.72. If we want to avoid trigonometry, then we can measure this from a simple geometric diagram. Because the simple epicyclic theories of the planets are inherently inaccurate, we will need to average several observations of maximum elongation to derive a good mean figure for the radius of the epicycle.

3.2.2 Superior Planet parameters

Suppose (Π, Y) is a period relation for a superior planet P. We employ the second deferent-and-epicycle model shown in Supplementary Fig. S6c. The rate of rotation of the deferent is ω_p rotations per year. By previous theory[21], for a superior planet:

$$\omega_p = (Y - \Pi)/Y$$

The rate of rotation of the epicycle in the "real world" is 1. Let D be the deferent, By the additive law of relative rotations, the rotation of the epicycle relative to the deferent, $\omega_{p\,|\,D}$, is defined by:

$1 = \omega_{p\,|\,D} + \omega_D$. In other words, $\omega_{p\,|\,D} = 1 - \omega_p = \Pi / Y$

Since a superior planet can be at any angle from the Sun, it has no maximum elongation from the Sun, so we cannot use the same method to determine r as for an inferior planet. We use the method of a previous publication[42], which showed how the parameters for

Ptolemy's more complicated theory[35] can be derived from observations using geometry. Our method uses the same ideas in a simpler context.

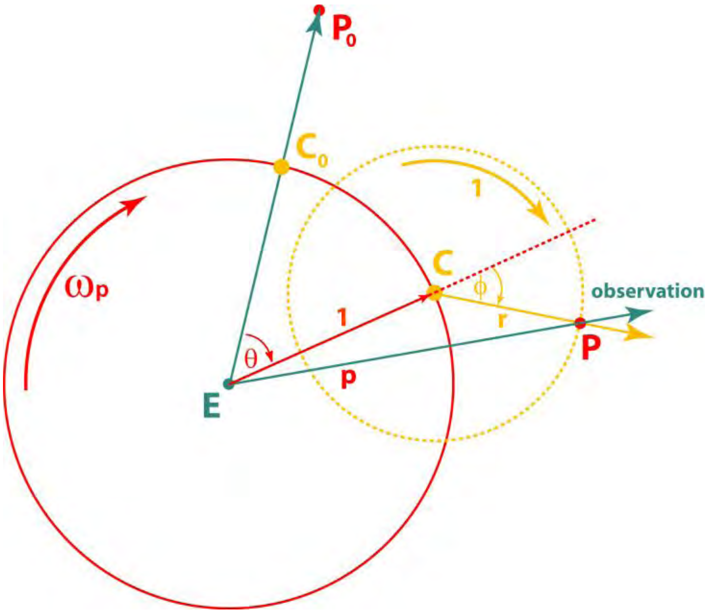

Supplementary Fig. S8. Superior planet: determination of the size of the epicycle, r, from observations

Let us assume that we have an observation of the planet at apogee (opposition), P_0, at time t = 0. Let us also assume that we have another observation of the angle of the planet, P, relative to apogee, at time t, where the observation is taken at a time substantially different from apogee. From our period relation (Π, Y), we know that the rate of rotation of the deferent is ω_p = (Y - Π)/Y, and the rate of rotation of the epicycle relative to the deferent is $\omega_{p\,|\,D}$ = 1 - ω_p = Π / Y, so we can calculate the angles θ, ϕ as:

$$\theta = \omega_p t = (Y - \Pi)\, t / Y$$

$$\phi = \omega_{p\,|\,D}\, t = (\Pi / Y)\, t$$

Now we can make a geometric diagram, where EC is at angle θ to EC_0, CP is at angle ϕ to EC (extended). Our observation of the planet tells us the angle of EP from EP_0. We can now observe where EP intersects CP and measure the length r of CP. This gives an estimate for the size of the epicycle. The reason for an observation *"substantially different from apogee"* is so that the angles of EP and CP are sufficiently different to determine an accurate intersection point. As for an inferior planet, the average of estimates of r, determined by several different observations, would be necessary to obtain a reliable mean value because of the inaccuracies inherent in the simple epicyclic theories (see next discussion).

For both inferior and superior planets, we can determine the sizes of the epicycles using straightforward geometry, without trigonometry.

3.3 Planet Periods

The key question remains as to what periods were used for the other planets, where the Front Cover inscriptional evidence is missing? How were the new periods (**289**, **462**) for Venus and (**427**, **442**) for Saturn discovered by the ancient Greeks? If we can understand this, then we might be able to infer the most likely periods that were used for the other planets, whose periods are missing.

3.3.1 New period relations from observations

In Babylonian astronomy, planetary period relations were derived either from observations, from combining previously known periods to get better ones, or a combination of both. It might be possible to derive the shorter *Goal-Year Periods* from observation, but the lengths of the *ACT Periods* (Supplementary Table S3, S4) mean that they could not have come directly from this source. A good example is the 1151-year period for Venus. A previous publication gives a persuasive explanation of this period[43]:

> *"It is not to be supposed that the large periods result directly from observation... A better explanation of the 1151-year Venus period can be found if we start with the 8-year period… …according to the Babylonian Venus tables... …in 5 synodic periods of Venus the Sun completes exactly 8 revolutions less 2½ degrees. Multiplying by 144, we find that the Sun completes 1151 revolutions in 720 synodic periods…*
>
> *This relation was continually used in the tables of the Seleucid Period."*

This shows how a more accurate period for a planet could be derived from an observation of the error of a less accurate period.

3.3.2 Errors in Period Relations

Since the synodic cycles involve both prograde and retrograde motions, in the past there has been some confusion about how errors should be measured. The following discussion assumes a modern heliocentric theory of the solar system, though it could be re-cast in a geocentric frame. We assume mean motions of the planets around the Sun.

Let P be an inferior planet with orbital period p_P years. Let the mean orbital rate of rotation of the planet be ω_P rotations per year. Then $\omega_P = 1/p_P$. From established theory[14], the synodic period, p_{syn}, of the planet is given by:

For an inferior planet: $p_{syn} = 1/(\omega_P - 1)$ years

For a superior planet: $p_{syn} = 1/(1 - \omega_P)$ years

We introduce two error parameters for period relations, expressed in degrees: ε_{syn} is the error of a period relation per synodic cycle; and ε_y is the error per year. ε_{syn} is useful to relate the cycle to observations or to combine period relations to get more accurate ones. ε_y measures the long-term accuracy of a period relation, compared to other planets.

For a period relation (Π, Y), we want the error parameter, $\varepsilon_{syn}(\Pi, Y)$, to satisfy;

Π synodic cycles = Y years + Π x $\varepsilon_{syn}(\Pi, Y)$ degrees

In modern astronomical terms:

$\Pi \times p_{syn} = Y + \Pi \times \varepsilon_{syn}(\Pi, Y)/360$

$\varepsilon_{syn}(\Pi, Y) = 360 \times (p_{syn} - Y/\Pi)$ degrees per synodic cycle (1)

Since the synodic periods of the planets are variable, we also define another notion of error, so that we can compare the errors in period relations between planets:

$\varepsilon_y(\Pi, Y) = \varepsilon_{syn}(\Pi, Y) / p_{syn}$ degrees per year (2)

For each planetary period relation, the ratio of the two error parameters $\varepsilon_{syn} / \varepsilon_y$ is independent of the period relation. So, the ordering of period relations by accuracy is the same, whichever error parameter we choose. However, the ordering of accuracy between planets is very different, since the ε_{syn} error is divided by p_{syn} to get the ε_y error.

3.3.3 Linear Combinations of Period Relations

The Babylonian astronomers used linear combinations of period relations to try and cancel out observed errors. The following discussion explores this idea and the possible consequences for the Antikythera Mechanism. A good example refers to Mercury. *Goal-Year Text* (GYT) periods for Mercury are (**41**,**13**), which has an attested error of -3 days, and (**145**,**46**), which has an attested error of -1 day (Supplementary Table S3). We can combine these to make:

(**394**, **125**) = 3 x (**145**, **46**) – 1 x (**41**, **13**), for a (supposedly) zero error.

We cannot use this for the Antikythera Mechanism, since the large prime factor of 394 is 197, which would imply a gear that is too large for the epicyclic system. There are only a handful of such Babylonian periods and errors that have come down to us, meaning that some of the periods could be justified based on errors unknown to us. Previous research[55] used linear combinations of known periods to give an interesting explanation of how Ptolemy (c.100-170 AD) derived the periods in *Planetary Hypotheses*.

Here we explore the principles behind using linear combinations of known period relations to derive better ones. Our aim is to find factorizable planetary periods that are of comparable accuracy as the **462**-year period for Venus and the **442**-year period for Saturn.

Let (Π_1, Y_1) and (Π_2, Y_2) be two period relations for a planet with errors $\varepsilon_{syn}(\Pi_1, Y_1)$ and $\varepsilon_{syn}(\Pi_2, Y_2)$.

Then, by the definition of ε_{syn}:

Π_1 synodic cycles = Y_1 years + Π_1 x $\varepsilon_{syn}(\Pi_1, Y_1)$ degrees

Π_2 synodic cycles = Y_2 years + Π_2 x $\varepsilon_{syn}(\Pi_2, Y_2)$ degrees

We shall use the obvious notation, familiar from vector algebra, for scalar multiplication and addition of period relations. Let m, n be positive or negative integers. Then we define:

$m(\Pi_1, Y_1) + n(\Pi_2, Y_2) = (m\Pi_1 + n\Pi_2, mY_1 + nY_2)$

For this combined period relation:

$(m\Pi_1 + n\Pi_2)$ synodic cycles

= $(mY_1 + nY_2)$ years + $(m\Pi_1 \times \varepsilon_{syn}(\Pi_1, Y_1) + n\Pi_2 \times \varepsilon_{syn}(\Pi_2, Y_2))$ degrees

To optimize the combined period relation, we want to choose m, n so that:

$m\Pi_1 \times \varepsilon_{syn}(\Pi_1, Y_1) + n\Pi_2 \times \varepsilon_{syn}(\Pi_2, Y_2) = 0$

In other words :

$n/m = -(\Pi_1 \times \varepsilon_{syn}(\Pi_1, Y_1))/(\Pi_2 \times \varepsilon_{syn}(\Pi_2, Y_2))$

We can use any integers—either positive or negative—providing the combined period relation has positive numbers. As an example, we explore linear combinations of period relations for Mercury. We make an improved period relation from the *Goal Year* period relations: (**41**, **13**), (**145**, **46**). Using errors from Supplementary Table S5:

n/m = - (0.062244 x 41)/(0.001689 x 145) = - 10.42

A reasonable approximation is m = -1, n = 10. For this we get:

-1 x (**41**, **13**) + 10 x (**145**, **46**) = (**1409**, **447**)

Since 1409 is prime, we try m = -1, n = 11.

-1 x (**41**, **13**) + 11 x (**145**, **46**) = (**1554**, **493**)

This is factorizable and yields excellent accuracy—though it is not the one we use because it does not lead to economical gear trains. We can use the same process for the other planets, but the problem is that the process *essentially* uses knowledge about errors in planetary period relations. Babylonian observational data is relatively crude, with errors summarized in Supplementary Tables S3, S4. We are sceptical that data existed in Babylonian astronomy to justify the Venus and Saturn periods. For our proposed planetary periods for the Antikythera Mechanism, we use a different process for generating linear combinations of planetary periods, described here as an *Unconstrained Parmenides Process* (Fig. 2c, d).

3.4 Possible Planet Periods

The maximum size of the planetary gears in the epicyclic system is determined by the periphery of **b1** (tip radius 65.0 mm) and the output tube system in the centre (radius 8.8 mm), leaving 65.0 – 8.8 = 56.2 mm for the diameter of any epicyclic gear. (For definitions of *pitch radius* and *module*, see Supplementary Discussion S4.) With an allowance for clearance, there is 52.0 mm for the tip diameter of the gear—so, a pitch radius of about 25.0 mm. With a module of 0.5, such a gear has 100 teeth. We therefore need all the epicyclic gears to have less than 100 teeth. We shall call a period relation *factorizable* if it can be expressed as a product of prime numbers less than 100. As a first step towards exploring suitable planetary periods, we used a VBA macro in Excel to generate *all* the possible periods with ε_y-accuracy better than 0.01°/year and using periods of less than 1,600 synodic cycles and 1,600 years—large enough numbers to include all the likely cycles for the Antikythera Mechanism. We are interested in period relations that can be expressed with gears of less than 100 teeth.

**MERCURY**					
**Orbital period**	**0.240840**	**Synodic period**	**0.317246** *years*		
Up to 1,600 cycles		***Error ε_y < 0.01°/year***			
Cycles	*Years*	*Error ε_y °/y*	*Error ε_{syn} °/syn*	*Factors*	*Factors*
1409	447	-0.000231	-0.000073		
**1554**	**493**	**0.000287**	**0.000091**	**2x3x7x37**	**17x29**
1264	401	-0.000868	-0.000275		
**1119**	**355**	**-0.001670**	**-0.000530**		
974	309	-0.002711	-0.000860		
829	263	-0.004117	-0.001306		
**1513**	**480**	**-0.005022**	**-0.001593**	**17x89**	**2^5x3x5**
**145**	**46**	**0.005323**	**0.001689**	**5x29**	**2x23**
**684**	**217**	**-0.006118**	**-0.001941**	**2^2x3^2x19**	**7x31**
1223	388	-0.007475	-0.002371		
**539**	**171**	**-0.009196**	**-0.002917**	**7^2x11**	**3^2x19**
Goal-Year Periods for comparison:					
**41**	**13**	**0.196201**	**0.062244**		

**VENUS**					
**Orbital period**	**0.615185**	**Synodic period**	**1.598654** *years*		
Up to 1,600 cycles		***Error ε_y < 0.01°/year***			
Cycles	*Years*	*Error ε_y °/y*	*Error ε_{syn} °/syn*	*Factors*	*Factors*
446	713	-0.000159	-0.000255		
593	948	0.000692	0.001107		
745	1191	-0.000837	-0.001338		
740	1183	0.001205	0.001927	2^2x5x37	7x13^2
887	1418	0.001548	0.002475		
299	478	-0.001848	-0.002954		
750	1199	-0.002852	-0.004560		
147	235	0.003275	0.005236	3x7^2	5x47
451	721	-0.003518	-0.005624		
603	964	-0.004346	-0.006948		
755	1207	-0.004841	-0.007738		
877	1402	0.005022	0.008029		
907	1450	-0.005169	-0.008264		
730	1167	0.005374	0.008591		
583	932	0.005903	0.009437		
436	697	0.006789	0.010853		
152	243	-0.006803	-0.010875	2^3x19	3^5
725	1159	0.007501	0.011992	5^2x29	19x61
917	1466	-0.008418	-0.013458		
**289**	**462**	**0.008576**	**0.013710**	**17^2**	**2x3x7x11**
765	1223	-0.008739	-0.013971		
613	980	-0.009220	-0.014739		
**720**	**1151**	**0.009658**	**0.015440**		
Goal-Year Periods for comparison:					
**5**	**8**	**-0.303105**	**-0.484560**		

**MARS**					
**Orbital period**	**1.880815**	**Synodic period**	**2.135312** *years*		
Up to 1,600 cycles		***Error ε_y < 0.01°/year***			
Cycles	*Years*	*Error ε_y °/y*	*Error ε_{syn} °/syn*	*Factors*	*Factors*
303	647	-0.000184	-0.000393		
739	1578	-0.000937	-0.002001		
473	1010	0.000992	0.002119		
436	931	-0.001460	-0.003118		
643	1373	0.001547	0.003303		
569	1215	-0.002140	-0.004569		
702	1499	-0.002562	-0.005470		
**170**	**363**	**0.003089**	**0.006596**	**2x5x17**	**3x11^2**
**133**	**284**	**-0.004368**	**-0.009326**	**7x19**	**2^2x71**
717	1531	0.004472	0.009550		
547	1168	0.004902	0.010468		
377	805	0.005720	0.012213	13x29	5x7x23
628	1341	-0.006386	-0.013636		
584	1247	0.006485	0.013848	2^3x73	29x43
495	1057	-0.006928	-0.014794		
362	773	-0.007869	-0.016803		
207	442	0.007880	0.016826	3^2x23	2x13x17
591	1262	-0.008657	-0.018486		
658	1405	0.009118	0.019469		
451	963	0.009686	0.020682		
229	489	-0.009903	-0.021146		
Goal-Year Periods for comparison:					
**15**	**32**	**0.333665**	**0.712478**		
**22**	**47**	**-0.177225**	**-0.378431**		
**37**	**79**	**0.029893**	**0.063830**		
**59**	**126**	**-0.047338**	**-0.101081**		

Supplementary Table S5. Accurate Planet Period Relations for Mercury, Venus & Mars. All planet period relations with ε_y-accuracy < 0.01°/year and involving < 1,600 synodic cycles and < 1,600 years for all planets, ordered by decreasing accuracy. Factorization is shown only if the factors are <100. Periods from the FCI outlined. Periods known from Babylonian astronomy or found on the Antikythera Mechanism are in bold type. For planets with unknown periods, candidate periods for the Antikythera Mechanism are highlighted in colour.

JUPITER

Orbital per. **11.861756** **Synodic per** **1.092066** *years*

Up to 1,600 cycles ***Error ε_y < 0.01°/year***

Cycles	*Years*	*ε_y °/y*	*ε_{syn} °/syn*	*Factors*	*Factors*
1336	1459	0.000085	0.000093		
1021	1115	-0.000137	-0.000149		
706	771	-0.000555	-0.000606		
**315**	**344**	**0.000802**	**0.000876**	**3^2x5x7**	**2^3x43**
1097	1198	-0.000945	-0.001032		
1184	1293	0.001611	0.001760		
**391**	**427**	**-0.001649**	**-0.001801**	**17x23**	**7x61**
**869**	**949**	**0.001905**	**0.002080**	**11x79**	**13x73**
1423	1554	0.002149	0.002347		
1249	1364	-0.002267	-0.002476		
554	605	0.002532	0.002765		
858	937	-0.002549	-0.002783		
1325	1447	-0.002814	-0.003073		
1347	1471	0.002936	0.003207		
793	866	0.003219	0.003515		
467	510	-0.003302	-0.003606		
**1032**	**1127**	**0.003588**	**0.003918**	**2^3x3x43**	**7^2x23**
1271	1388	0.003818	0.004169		
1010	1103	-0.003942	-0.004305		
543	593	-0.004492	-0.004906		
239	261	0.004812	0.005255		
**1162**	**1269**	**-0.004971**	**-0.005428**	**2x7x83**	**3^3x47**
619	676	-0.005390	-0.005887		
1358	1483	0.005742	0.006270		
**1314**	**1435**	**-0.005761**	**-0.006292**	**2x3^2x73**	**5x7x41**
1119	1222	0.005940	0.006487		
**695**	**759**	**-0.006092**	**-0.006653**	**5x139**	**3x11x23**
**880**	**961**	**0.006247**	**0.006822**	**2^4x5x11**	**31^2**
771	842	-0.006655	-0.007268		
641	700	0.006782	0.007406		
**847**	**925**	**-0.007118**	**-0.007773**	**7x11^2**	**5^2x37**
1043	1139	0.007234	0.007900		
1445	1578	0.007434	0.008118		
**923**	**1008**	**-0.007504**	**-0.008194**	**13x71**	**2^4x3^2x7**
999	1091	-0.007831	-0.008552		
402	439	0.007954	0.008686		
1075	1174	-0.008112	-0.008859		
1151	1257	-0.008356	-0.009125		
**1369**	**1495**	**0.008502**	**0.009285**	**37x37**	**5x13x23**
1227	1340	-0.008570	-0.009359		
967	1056	0.008730	0.009534		
1303	1423	-0.008759	-0.009565		
1379	1506	-0.008927	-0.009748		
1455	1589	-0.009077	-0.009913		
565	617	0.009283	0.010137		
1293	1412	0.009696	0.010589		
Goal-Year Periods for comparison:					
**65**	**71**	**-0.072896**	**-0.079607**		
**76**	**83**	**-0.011807**	**-0.012894**		

SATURN

Orbital per. **29.456522** **Synodic per** **1.035141** *years*

Up to 1,600 cycles ***Error ε_y < 0.01°/year***

Cycles	*Years*	*ε_y °/y*	*ε_{syn} °/syn*	*Factors*	*Factors*
1309	1355	0.000000	0.000000		
996	1031	0.000258	0.000267		
683	707	0.000752	0.000778		
313	324	-0.000820	-0.000849		
1053	1090	0.001219	0.001262		
1423	1473	0.001443	0.001494		
1508	1561	-0.001532	-0.001586		
1195	1237	-0.001718	-0.001779		
882	913	-0.002037	-0.002109	2x3^2x7^2	11x83
370	383	0.002081	0.002154		
1451	1502	-0.002300	-0.002380		
1537	1591	0.002672	0.002766	29x53	37x43
569	589	-0.002706	-0.002802		
1167	1208	0.002859	0.002960		
1394	1443	-0.003130	-0.003240	2x17x41	3x13x37
797	825	0.003220	0.003334		
825	854	-0.003422	-0.003542	3x5^2x11	2x7x61
1224	1267	0.003565	0.003690		
1081	1119	-0.003799	-0.003932		
1337	1384	-0.004031	-0.004173		
**427**	**442**	**0.004208**	**0.004356**	**7x61**	**2x13x17**
1338	1385	0.004796	0.004964		
256	265	-0.005013	-0.005189	2^8	5x53
911	943	0.005071	0.005250		
1395	1444	0.005336	0.005523	3^2x5x31	2^2x19^2
484	501	0.005833	0.006038		
1479	1531	-0.005900	-0.006108		
1223	1266	-0.006086	-0.006300		
1509	1562	0.006293	0.006515		
967	1001	-0.006370	-0.006594		
1025	1061	0.006511	0.006739		
711	736	-0.006859	-0.007100	3^2x79	2^5x23
541	560	0.007117	0.007367		
1166	1207	-0.007264	-0.007519	2x11x53	17x71
1139	1179	0.007662	0.007931		
455	471	-0.007897	-0.008175		
598	619	0.008155	0.008442		
1109	1148	-0.008563	-0.008864		
1253	1297	0.008603	0.008906		
655	678	0.009013	0.009330	5x131	2x3x113
654	677	-0.009026	-0.009343		
1507	1560	-0.009367	-0.009696		
1367	1415	0.009388	0.009718		
853	883	-0.009628	-0.009967		
712	737	0.009733	0.010075	2^3x89	11x67
Goal-Year Periods for comparison:					
**29**	**30**	**0.221402**	**0.229183**		
**57**	**59**	**0.018012**	**0.018645**		
**142**	**147**	**-0.023496**	**0.023496**		

Supplementary Table S6. Accurate Planet Period Relations for Jupiter & Saturn. All planet period relations with ε_y-accuracy < 0.01°/year and involving < 1,600 synodic cycles and < 1,600 years for all planets, ordered by decreasing accuracy. Factorization is shown only if the factors are <100. Periods from the FCI outlined. Periods known from Babylonian astronomy or found on the Antikythera Mechanism are in bold type (Supplementary Tables S3, S4). Candidate periods for the Antikythera Mechanism are highlighted in colour.

Despite these tables being exhaustive within the range of cycles under consideration, there are not very many good options for factorizable period relations, which are of comparable accuracy to the **462**-year period for Venus and the **442**-year period for Saturn.

The question arises as to whether the period relations that we have chosen are unique. Could different seed periods or more iterations of the *Unconstrained Parmenides Process* yield equally valid period relations? For the inferior planets, the period relation (**289**, **462**) for Venus means that the shared prime in the number of synodic cycles must be **17**. For Mercury, Supplementary Table S5 shows that (**1513**, **480**) is unique in having the factor **17**. The period relation (**427**, **442**) for Saturn means that the shared prime must have been **7**. For Mars, Supplementary Table S5, shows that (**133**, **284**) is unique in having the factor **7**.

For Jupiter, Supplementary Table S6 shows three options with factors of **7** for the synodic cycles: (**315**, **344**), (**847**, **925**), (**1162**, **1269**). So, the period relation for Jupiter is not a unique logical consequence of our criteria. We used an Excel VBA macro to check if these can be obtained from known Babylonian seed periods in Table S4, using the Unconstrained Parmenides Process. There are a number of ways of doing this, the simplest being:

(**847**, **925**) = **3** x (**87**, **95**) + **2** x (**293**, **320**)

(**1162**, **1269**) = **5** x (**76**, **83**) + **2** x (**391**, **427**)

In each planetary gear train, there are two gears for the synodic cycles and two gears for the years (Figure 3). For a gear train expressing (**847**, **925**) = (**7** x **11** x **11**, **5** x **5** x**37**), the first fixed gear must have **77** teeth. Since this is shared between Mars and Saturn, their mechanisms must also have a fixed gear with **77** teeth. For Saturn, (**427**, **442**) = (**7** x **61**, **2** x **13** x **17**). The gear train must have a multiplier of **11** to incorporate the **77**-tooth gear. Thus, the factors **2** x **13** x **17** x **11** must be shared between the two gears for the years, which is not possible while keeping the tooth counts < 100, as required. So, (**847**, **925**) is not a viable period relation for Jupiter. This leaves (**1162**, **1269**) = (**2** x **7** x **83**, **3** x **3** x **3** x **47**) for Jupiter. Though theoretically possible, (**1162**, **1269**) entails more iterations of the Unconstrained Parmenides Process. For this period relation, we need a factor of **4** to enable the first fixed gear to have **56** teeth. This means that the second gear for the synodic cycles must have **83** teeth. In this context, it is no longer possible for the Jupiter gear train to share its second gear with that of Mars, violating our *Reconstruction Principle: Prefer simple and economical solutions.* The factors for the gears **g2** and **g5** must come from **3** x **3** x **3** x **47** x **4**. The factor **4** must be shared between the gears, since **27** x **4** = **108** and **47** x **4** = **188** are too large. This means that one of the gears **g2**, **g5** must have **94** teeth. It is possible to fit all the resulting gear systems on the **CP**, though such large gears have more mechanical problems with more teeth to cut. Also, the neat idea of aligning the outputs with cardinal axes must be abandoned. By contrast, (**315**, **344**) can be obtained from simple seed periods and was itself known in Babylonian astronomy. It leads to modest-sized gears and is more accurate than the other two. We are confident that (**315**, **344**) was the period relation used for Jupiter.

The following table shows how the errors relative to the true value develop through the *Unconstrained Parmenides Process* (Fig. 2).

MERCURY			
p	q	r	s
145	46	684	217
**0.005323**		**-0.006118**	
$p+r$	$q+s$		
829	263		
-0.004117			
$2p+r$	$2q+s$	$p+2r$	$q+2s$
974	309	1513	480
-0.002711		**-0.005022**	
$3p+r$	$3q+s$	$p+3r$	$q+3s$
1119	355	2197	697
-0.001670		-0.005363	

VENUS			
p	q	r	s
5	8	720	1151
**-0.303105**		0.009658	
$p+r$	$q+s$		
725	1159		
0.007501			
$2p+r$	$2q+s$	$p+2r$	$q+2s$
730	1167	1445	2310
0.005374		**0.008576**	
$3p+r$	$3q+s$	$p+3r$	$q+3s$
735	1175	2165	3461
**0.003275**		0.008936	

MARS			
p	q	r	s
37	79	133	284
**0.029893**		**-0.004368**	
$p+r$	$q+s$		
170	363		
**0.003089**			
$2p+r$	$2q+s$	$p+2r$	$q+2s$
207	442	303	647
**0.007880**		-0.000086	
$3p+r$	$3q+s$	$p+3r$	$q+3s$
244	521	436	931
0.005254		-0.001460	

JUPITER			
p	q	r	s
76	83	315	344
**-0.011807**		**0.000802**	
$p+r$	$q+s$		
391	427		
**-0.001649**			
$2p+r$	$2q+s$	$p+2r$	$q+2s$
467	510	706	771
-0.003302		-0.000555	
$3p+r$	$3q+s$	$p+3r$	$q+3s$
543	593	1021	1115
-0.004492		-0.000137	

SATURN			
p	q	r	s
57	59	256	265
**0.018012**		**-0.005013**	
$p+r$	$q+s$		
313	324		
-0.000820			
$2p+r$	$2q+s$	$p+2r$	$q+2s$
370	383	569	589
0.002081		-0.002706	
$3p+r$	$3q+s$	$p+3r$	$q+3s$
427	442	825	854
**0.004208**		**-0.003422**	

Supplementary Table S7. Table 2D expanded with ε_y errors at each stage. Period relations <100-factorizable are highlighted in colour. For Venus: (**1445**, **2310**) ≡ (**289**, **462**) and (**735**, **1175**) ≡ (**147**, **235**).

4. Supplementary Discussion S4: Theoretical Mechanisms

4.1 Gear Trains

Throughout this discussion, we use the same basic notation for gear trains as described in the figure legend for Fig. 3 in the Main Text. Fixed gears are underlined; **blue** gears calculate **synodic** cycles; **red** gears calculate **years**; **black** gears are **idler gears**: all designated by their tooth counts. "~" means "*meshes with*"; "+" means "*fixed to the same arbor*"; "⊕" means "*with a pin-and-follower, turning on the central axis*" or "*with a pin-and-slot on eccentric axes*"—creating **variable motion**, shown in **turquoise**. *Followers* are slotted rods that follow a pin on the epicyclic gear and pivot on the central axis.

The *pitch circle* of a gear is a theoretical circle, which defines where the gear interacts without slipping with a meshed gear. For gears with triangular teeth, we take the pitch circle as half-way between the inner and outer teeth circles. The *pitch diameter* and *pitch radius* refer to the *pitch circle*. The *module* of a gear is the ratio, *pitch diameter* / *number of teeth*.

4.1.1 Gear modules in the Antikythera Mechanism

	Parameters						*Meshing Parameters*					
Gear	*Count*	*Outer r*	*Inner r*	*Pitch r*	*Thick*	*Mod*	*Mesh*	*Mod*	*Mis-M*	*Mesh*	*Mod*	*Mis-M*
**a1**	48						**b1**					
**b1**	223	65.0	63.8	64.4	2.7	0.578	**a1**					
**b2**	64	15.7	15.0	15.3	2.3	0.479	**c1**	0.517	7.6%	**l1**	0.455	5.3%
**b3**	32	9.2	8.3	8.7	1.3	0.546	**e1**	0.572	4.8%			
**c1**	38	10.3	9.4	9.8	1.5	0.517	**b2**	0.479	7.6%			
**c2**	48	11.0	10.5	10.7	1.3	0.447	**d1**	0.452	1.1%			
**d1**	24	5.8	5.1	5.4	2.4	0.452	**c2**	0.447	1.1%			
**d2**	127	31.7	30.6	31.2	1.3	0.491	**e2**	0.465	5.4%			
**e1**	32	9.7	8.6	9.2	1.3	0.572	**b3**	0.546	4.8%			
**e2**	32	7.8	7.1	7.4	1.0	0.465	**d2**	0.491	5.4%			
**e3**	223	52.4	51.5	51.9	1.4	0.466						
**e4**	188	49.9	49.1	49.5	1.5	0.526	**f1**	0.499	5.4%			
**e5**	50	13.1	12.2	12.6	0.5	0.505	**k1**	0.519	2.8%			
**e6**	50	13.9	12.9	13.4	0.6	0.536	**k2**	0.541	1.0%			
**f1**	53	12.9	13.6	13.2	1.3	0.499	**e4**	0.526	5.4%			
**f2**	30	8.2	7.4	7.8	1.2	0.522	**g1**	0.513	1.6%			
**g1**	54	14.4	13.4	13.9	1.5	0.513	**f2**	0.522	1.6%			
**g2**	20	4.9	4.1	4.5	1.6	0.451	**h1**	0.445	1.2%			
**h1**	60	13.7	13.0	13.4	1.0	0.445	**g2**	0.451	1.2%			
**h2**	15	3.8	3.0	3.4	1.4	0.454	**i1**	0.429	5.6%			
**i1**	60	13.2	12.6	12.9	1.2	0.429	**h2**	0.454	5.6%			
**k1**	50	13.3	12.6	13.0	0.6	0.519	**e5**	0.505	2.8%			
**k2**	50	14.0	13.1	13.5	0.5	0.541	**e6**	0.536	1.0%			
**l1**	38	9.0	8.3	8.6	1.5	0.455	**b2**	0.479	5.3%			
**l2**	53	13.3	12.5	12.9	1.5	0.487	**m1**	0.503	3.2%			
**m1**	96	24.7	23.6	24.1	2.0	0.503	**l2**	0.487	3.2%			
**m2**	15	4.0	3.7	3.9	1.8	0.514						
**o1**	60	12.8	12.2	12.5	1.1	0.417						
**q1**	20											
**r1**	63	16.9	15.9	16.4	1.6	0.521						
					Mean:	0.495		Mean	3.7%			

Supplementary Table S8. Gear parameters of the surviving gears. Measurements are in millimetres. The mean module is 0.495. **a1** and **q1** are contrate gears, so the parameters do not apply. The parameters are derived from measurements made on the X-ray CT data, using *VGStudio Max* (Volume Graphics), which enables highly accurate measurements.

All the gears in the Antikythera Mechanism have a module in a narrow range centred on 0.5, when the pitch diameter is measured in millimetres. The right-hand side of the table shows

which gears mesh with which others and the mis-match between the modules of meshing gears. We introduce a *mis-match parameter*, defined as:

Mis-match(m1, m2) = abs(m1 – m2)/average(m1, m2), expressed as a percentage.

In the Antikythera Mechanism, the modules of meshing gears are similar, as the table shows—with a mean mis-match of only 3.7% and a maximum mis-match of 7.6%. If we have two meshing gears **g1**, **g2**, with modules m1, m2, then we can calculate the approximate distance between their centres as (g1m1 + g2m2)/2, though in practice the loose mesh of gears enforced by triangular tooth profiles, means an additional allowance must be made.

4.1.2 Calculating Gear Rotations

The rotation of a gear **g** in rotations per year will be referred to as: ω(**g**). If a gear is mounted on an *epicyclic table* (a gear or a rotating plate attached to a gear) **S**, then its rotation relative to **S** will be referred to as ω(**g** | **S**). We adopt the convention that rotations, which are clockwise when seen from the front, are positive. Our proofs of the integrity of our proposed mechanisms are given in detail from first principles, since many mistakes have often been made in the past.

4.1.3 Laws of Meshing Gears

Basic Law

If gear **g1** meshes with gear **g2**, then:

ω(**g2**) = - g1/g2 x ω(**g1**)

g1ω(**g1**) + g2ω(**g2**) = 0

Relative Basic Law

If gear **g1** meshes with gear **g2** on an epicyclic Supplementary Table S, then:

ω(**g2** | **S**) = - g1/g2 x ω(**g1** | **S**)

g1ω(**g1** | **S**) + g2ω(**g2** | **S**) = 0

Additive Law of Relative Rotations

If gear **g** is mounted on an epicyclic table **S** (either **b1** or the **Circular Plate** (**CP**), then:

ω(**g**) = ω(**g** | **S**) + ω(**S**)

All the rotations in our gear trains will be calculated using these basic laws. The relative law is simply the basic law relativized to a rotating frame-of-reference. In all our mechanisms, **g1** is a fixed gear. Everything is turned by the epicyclic mounting of **g2**, which is dragged round **g1** by **b1** and the **CP**.

4.2 Nodes & Inferior Planet Mechanisms

This section develops theoretical mechanisms for the *Nodes* and *Inferior Planets*, based on period relations summarized in Fig. 4e. We also prove that these mechanisms model the correct variable motions, as embodied in the ancient Greek epicyclic theories of the planets.

4.2.1 Line of Nodes of the Moon

The *Line of Nodes* rotates backwards at a very slow rate, which we will assume is constant—so we do not need any device to model variability. First, we show how a 4-gear epicyclic system can model such a slow rotation with a period of 18.6 years.

Fig 2A shows the system: **g1** ~ **g2** + **g3** ~ **g4**, mounted on the rotating gear **b1**.

By the basic law of meshing gears relativized to **b1**:

ω(**g1** | **b1**) = - g2/g1 ω(**g2** | **b1**)

Since **g1** is fixed:

ω(**g1** | **b1**) = -1

ω(**g2** | **b1**) = g1/g2 = ω(**g3** | **b1**) (since **g3** is rigidly attached to **g2**)

By the basic law of meshing gears relativized to **b1**:

ω(**g4** | **b1**) = - g3/g4 ω(**g3** | **b1**) = - (g3/g4) x (g1/g2) = (g1 x g3)/(g2 x g4)

By the basic law of relative rotations:

$\omega(\mathbf{g4}) = \omega(\mathbf{g4} \mid \mathbf{S}) + \omega(\mathbf{b1}) = 1 - (g1 \times g3)/(g2 \times g4)$ (1)

In order that a small adjustment of the modules of **g3** and **g4** will allow their axes to exactly coincide, we also want:

$g1 + g2 \simeq g3 + g4$ (2)

We look at practical choices for the gears to calculate the correct rotation for the *Line of Nodes* and to match the bearing in Spoke B. We do not use a previous suggestion[9] that the *Line of Nodes* rotation was calculated as - $^{12}/_{223}$ (derived from the Metonic and Saros Cycles), because the prime number 223 is too large to mechanize in this epicyclic context. We use a more accurate period, 18.6 years, which was a standard period for the *Line of Nodes* in the ancient world[14].

By equation (1), we need to satisfy:

$\omega(\mathbf{g4}) = 1 - (g1 \times g3)/(g2 \times g4) = -5/93$

$(g1 \times g3)/(g2 \times g4) = 98/93 = (2 \times 7 \times 7)/(3 \times 31)$

In other words, the rate of rotation *relative to* ***b1*** is $^{98}/_{93}$. We need to multiply this by an integer to make reasonable gears. We also want the bearing of **g2** + **g3** to be at 27.0 mm from the central axis so that it matches the bearing in Spoke B (Fig. 4f) and for the gears to satisfy (2), with a small adjustment to their modules. We assume that meshing gears have the same module. Let the modules of **g1**, **g2** be m1 and of **g3**, **g4** be m2. For close-meshing gears, the interaxial distance of two meshing gears, **g1** and **g2**, with the same module, m1, is m1(g1 + g2)/2. So, we want:

$m1(g1 + g2)/2 = m2(g3 + g4)/2$ (3)

With some trial-and-error, we find a multiplier of 32 and the gear train: **<u>49</u>** ~ **62** + **64** ~ **48**. For this train: g1 + g2 = 111 and g3 + g4 = 112. To put the axis of **g2** + **g3** at 27.0 mm from the central axis, we need:

$m1(49 + 62)/2 = 27.0$. In other words: $m1 = 0.486$

To make the axis of **g4** coincide with the central axis, by (3), we need:

$m2 = m1(g1 + g2)/(g3 + g4) = 0.482$

In practice, allowances must be made for the necessary loose-fitting of gears with triangular teeth that are typical in the Antikythera Mechanism (otherwise the gears bind). Trial-and-error suggests that a module of 0.482 for **<u>49</u>** and **62** and a module of 0.478 for **64** and **48** give a close match to the evidence. These modules are well within the range of modules of the surviving gears (Supplementary Table S8). There is precedent for adjusting modules in this way: the four central gears of the lunar anomaly epicyclic system, **e5** ~ **k1** ⊕ **k2** ~ **e6** have a very similar structure[7] and their modules are adjusted so the output gear **e6** is on axis **e**.

4.2.2 Inferior Planet Mechanisms

We will prove that our proposed gear mechanisms (Fig. 4) turn the planetary mechanisms at the correct rate to match the ancient Greek epicyclic theories of the planets. These proofs are written in the mathematical language of vectors. The ancient Greeks did not have access to vectors, but all the ideas are essentially simple geometry, which the ancient Greeks understood very well. For the inferior planets we will use *direct mechanisms* and for the superior planets *indirect mechanisms*[11,9]. In the context of the superior planets, there is a discussion below about the difference between *direct* and *indirect mechanisms*.

It was observed previously[16] that the inferior planets cannot be modelled with indirect epicyclic mechanisms: they could only be modelled with indirect mechanisms if the stepped arbor for the pin-and-slot were fixed, which does not conform to the epicyclic structure of the front of the Antikythera Mechanism. The same applies to the *true Sun*. This is all correct except in the unlikely circumstance where some of the gears are in turn mounted epicyclically on other epicyclic gears. It is possible to devise such a theoretical system, but the complexity of such systems rules them out for the Antikythera Mechanism. In the epicyclic context at the front of the Antikythera Mechanism, it is not possible to devise

reasonable and economical *pin & slot* devices for the inferior planets. We therefore adopt the simpler *pin-and-slotted follower* devices, first proposed for the Antikythera Mechanism in 2002[3] and found in Giovanni de Dondi's 14th century AD astronomical clock[36].

Some previous mechanisms employed just two gears[21,9] in a direct mechanism. These were based on the simple GYT period relations (Supplementary Table S3), with the tooth counts of the gears simply reflecting the ratio of the number of synodic cycles to the number of years in the period relation. For the (**5**, **8**) period relation for Venus, a model can be made using just two gears—for example: **50** ~ **80** ⊕ **follower**. To model the more complex period relations for the Antikythera Mechanism, we need compound gearing.

The system: **g1** ~ **g2** + **g3** ~ **g4 ~ g5** ⊕ **follower**, mounted on the rotating gear **b1** is shown in Fig. 3c. Our calculations are based on a heliocentric model of the Solar System, but the parameters could be derived from a geocentric model. We assume the period relation (Π, **Y**). The diagram refers to Venus, but the proof is given for any inferior planet. It does not matter if the idler gear **g4** is before or after **g3** in the gear train: it is simply there to reverse the direction of rotation. The pin is attached to a disk, which is rigidly attached to **g5**, so that the pin can be placed outside the face of the gear[3]. The centre of **g5** can be placed anywhere, provided the pin can be placed in the correct place on the disk.

Let the pin distance from the centre of **g5** be *d* and the distance of the planet from the Sun in AU be *p*. Let *i* be the distance between the centres of **g1** and **g5**. By previous theory[21]:

$$d = p \times i$$

We want to establish the equivalence of the 5-gear inferior planetary mechanism to ancient Greek epicyclic theory. We assume that a multiple of the chosen period relation (Π, **Y**) of the planet can be factorized as (g1 x g4**,** g3 x g5). The multiple is chosen to get reasonably-sized gears, which fit onto **b1**.

Let r' be the period calculated from (Π, **Y**) of the planet round the Sun in years. Within the accuracy of the period relation, for an inferior planet[21]:

$$1/r' = 1 + \Pi / Y$$

By the basic law of meshing gears in the frame-of-reference of **b1**:

$$\omega(g2 \mid b1) = -\omega(g1 \mid b1) \times g1/g2$$

Because **g1** is fixed, it rotates backwards relative to **b1** at 1 rotation per year. In other words:

$$\omega(g1 \mid b1) = -1$$

$$\omega(g2 \mid b1) = g1/g2$$

Because **g3** is fixed onto the same arbor as **g2**:

$$\omega(g3 \mid b1) = \omega(g2 \mid b1)$$

Because **g4** is an idler gear, by the basic law of meshing gears:

$$\omega(g5 \mid b1) = \omega(g3 \mid b1) \times g3/g5 = g1/g2 \times g3/g5 = (g1 \times g3)/(g2 \times g5) = \Pi / Y$$

Using the basic law of relative rotations:

$$\omega(g5) = \omega(g5 \mid b1) + \omega(b1) = 1 + \Pi / Y = 1/r'$$

So, **g5** turns at the correct rate. Referring to Fig. 3d in the Main Text, the vector from the centre of **g5** to the **pin** will be called **red**. The vector from the central axis (centre of **b1)** to the **pin** will be called **turquoise**. The vector from the central axis to the centre of **g5** will be called **black**. Then:

turquoise = **black** + **red**

ω(**black**) =1

ω(**red**) = ω(**g5**) = 1/r'

So, the rotation of **turquoise**, which is attached to the output follower is the sum of the **black** vector rotating at 1 rotation per year and the **red** vector rotating at the estimated rate 1/r' of the planet round the Sun. This is exactly the ancient Greek epicyclic theory of the planet, within the accuracy of the period relation.

4.3 True Sun & Superior Planet Mechanisms

This section develops theoretical mechanisms for the true Sun and Superior Planets, based on period relations summarized in Fig. 2f in the Main Text. We also prove that these mechanisms model the correct variable motions, as embodied in the ancient Greek epicyclic theories of the planets.

4.3.1 True Sun Mechanism

Since the lunar anomaly is known to have been mechanized in the Mechanism, it seems natural that the solar anomaly would also have been mechanized, as suggested previously[3]. It is much easier to mechanize than either the lunar or the planetary anomalies, since the period of the solar anomaly is the same as the annual cycle. The system: **g1** ~ **g2** ~ **g3** ⊕ **follower**, mounted on the rotating *Circular Plate* (**CP**) is shown in Fig. 3b. The proof that it models the ancient Greek epicyclic theory uses similar principles to our previous proofs.

4.3.2 *Direct* and *Indirect Mechanisms* for the Superior Planets

The paper, *A Planetarium Display for the Antikythera Mechanism*[3], uses mechanisms for the superior planets that directly model the ancient Greek epicyclic theories, according to the second version of the deferent-and-epicycle theory in Supplementary Fig. S6c. A "table" (disk or gear) to carry an epicycle is turned at the mean rate of the planet round the Sun, ω_P. On this is mounted an epicycle that turns at the rate of the mean Sun in the real world. A pin mounted on the epicycle is tracked by a slotted follower that pivots on the central axis, to which is attached an output tube that transmits the variable output to the Front Dials. Let us assume that we would like to use a direct mechanism to model the period relation (Π, Y). From Supplementary Discussion S3, we know that for a superior planet, $\omega_P = 1 - \omega_{syn}$. We therefore need to turn the deferent at the rate $1 - \Pi/Y$. For example, in a subsequent paper[16], the author chose the period relation (**133**, **284**) for Mars—the same period relation that we have chosen for our Mars mechanism, using the criterion of *economy*. The author therefore had to turn the deferent at the rate 1 – 133/284 = 151/284 rotations per year. This ratio gave the author considerable difficulties: the numbers do not favour direct models. 151 is an inconvenient prime number, which is too large for the Antikythera Mechanism's epicyclic system and the author struggles to find a suitable ratio. After several pages of ad hoc approximations and some dexterity with the numbers:

> *…we obtain 1166 : 1027. This is an excellent approximation, with terms that can be factorized thus: 2 × 11 × 53 : 13 × 79 and again, these factors may easily be distributed in a workable three-pair train.*

It does seem very unlikely that the ancient Greeks found this ratio for use in a direct model using a three-pair train. By contrast, in our model, we use *indirect mechanisms* for the superior planets[11,9]. These are analogous to the way that the lunar anomaly was mechanized in the Antikythera Mechanism, as previously established[7]. The mechanisms have a table that turns at the rate of the mean Sun. Mounted on this is an epicyclic pin-and-slot device, where the pin gear turns at the rate Π / Y rotations per year, *relative to the table*, using a two-pair train. The author of the previous publication disparaged such indirect mechanisms[16]:

> *The scheme is neat, compact and economical, but it is limited by the use of simple gear-pairs to ratios with terms of moderate size such as those derived from the Goal-Year periods. The long-term accuracy of indication is correspondingly poor.*

He goes on to entertain the possibility of using compound gear trains for such mechanisms to improve accuracy but concludes that such gear trains would be too complex and would have mechanical problems, concluding that direct mechanisms would be *"…more easily made mechanically sound."* We do not agree with this assessment. Using compound gear trains, mechanically sound *indirect mechanisms* can model planetary period relations, with fewer gears and at least as much accuracy as *direct mechanisms*. In these models, the rotation of the epicycle relative to the deferent is simply Π / Y, which means that gears can be chosen to model the period relation without difficulty or approximation. A good example is Mars, where we use the ratio 133/284 = (7 x 19)/(2^2 x 71) and this is easily and exactly

modelled by a two-pair compound gear train with reasonably-sized gears that can be mounted on the **Circular Plate** (**CP**).

One basic problem with direct mechanisms is that they lead to a very bulky architecture for the Antikythera Mechanism, which directly contradicts the basic structure of pillars, **Strap** and **CP**, as discussed further in Supplementary Discussion S6. If two superior planets were mounted on the same plate, then they would interfere with each other mechanically. One great advantage of using *indirect mechanisms* for the superior planets is that they can be mounted together on the same plate without obstructing each other. The outputs are on gears attached to tubes at the centre. In other words, there are no central followers to generate the variable motion because this is generated by the pin-and-slot devices mounted epicyclically on the face of the **CP**. Our indirect mechanisms are conceptually harder to understand but result in very compact designs that use the factorizable period relations that are generated by the *Unconstrained Parmenides Process* in a straightforward way.

The designer may have first developed indirect mechanisms for the planets because of their multiple advantages and then applied similar ideas to model the lunar anomaly[7]. The epicyclic theories of the planets model the anomalies created by a geocentric frame, whereas the mechanism that models the lunar anomaly compensates for the elliptical orbit of the Moon, where the major axis—the *Line of Apsides* of the Moon—rotates. Remarkably, the same idea of adding two circular motions to model variable motion works for both situations.

4.3.3 Proofs that the Superior Planet Mechanisms match the Epicyclic Theory

Our superior planet mechanisms are mounted on the **CP** (Fig. 6a, b). We use *indirect mechanisms*[11,9] to achieve an economical structure, which exactly matches the surviving evidence from the pillars on **b1** and attached plates. These mechanisms also have a persuasive precedent in the lunar anomaly mechanism[7]. Therefore, they conform well to our *Reconstruction Principles* (Supplementary Discussion S1). Here we prove that they model the ancient Greek epicyclic theories. The proofs are analogous to the proof that the lunar anomaly mechanism models the ancient Greek *Epicyclic Theory of the Moon*[7]. Previous research[9] used 4-gear planetary mechanisms, based on period relations from the GYT (Supplementary Discussion S3), without compound gearing. We demonstrate novel 7-gear mechanisms that model more complex period relations with compound gearing.

The system shown in Fig. 3d is: **g1** ~ **g2** + **g3** ~ **g4** ~ **g5** ⊕ **g6** ~ **g7**. It is mounted on the rotating **CP**, which turns at the rate 1 rotation per year. **g7** has a tube attached, which carries the output to the Cosmos display. Though the diagram is seen from the back, rotations will all be measured as positive if they are clockwise, as seen from the front. These 7-gear mechanisms modify the previously-introduced 4-gear mechanisms[11,9] by employing compound gearing to turn the epicyclic gears more accurately, based on improved period relations. The diagram is suitable for Mars, but the proof applies to all the superior planets. (Note that the pin is not on the edge of **g5** for Mars because this would entail a **g6** gear that is too large to fit on the **CP**.) The essence of these superior planet mechanisms is that a compound gear train turns a gear **g5** (in the *wrong sense*) at the rate of the mean synodic rotation of the planet r*elative to the* ***CP*** and this is then translated into a variable motion (in the *wrong sense*) by a pin mounted on **g5** that engages with a slot on **g6**, where these two gears are mounted on eccentric axes. This variable output is then reversed to the *correct sense* by engagement with an equal output gear **g7**. The symmetry of the output of the pin-and-slot device ensures that when the output sense is reversed, it will model the epicyclic theory. An alternative is to put the idler gear between the two gears **g6**, **g7**. (In this case, the proof is similar, except the *mirrors* described below are exchanged for *magic mirrors*, which reflect objects without mirror reversal—like synchronized swimmers!)

Let (Π, **Y**) be the estimated period relation for the superior planet. Let r' be the calculated period of the planet round the Sun in years. Then by previous theory[21]:

$$1/r' = 1 - \Pi\ /\ \mathbf{Y}$$

We refer to Fig. 3d in the Main Text. Let d be the distance of the pin on **g5** from the centre of **g5** and p be the distance in AU of the planet from the Sun. Then by established theory[21]:

Offset = Offset (**g5**, **g6**) = d/p

The epicyclic gears are attached to the **CP**, which is indicated by the yellow arc to the right and rotates clockwise (when seen from the front) at 1 rotation per year. The pin-gear **g5** turns the slot gear **g6**, mounted on an eccentric axis, at a variable rate—in the same way as the lunar anomaly mechanism in the Antikythera Mechanism[7]. We create two "mirrors" attached to the **CP**. These rotate with the **b1-CP** assembly reflecting the image of an object. The first mirror, the **red mirror** (shown by the dotted red line), is placed halfway between the central axis and the axis of **g5**. This reflects the **pin** to its mirror image, **pin'**. The second mirror, the **turquoise mirror** (shown by the dotted turquoise line), is placed halfway between the central axis and the axis of the slot gear **g6**. This reflects the gear **g6** and the **pin** to their mirror images in the **turquoise mirror**, **g7** and **pin''**. Notice that **pin''** is attached to **g7**.

By the basic law of meshing gears:

$$\omega(\mathbf{g2} \mid \mathbf{CP}) = -\,\omega(\mathbf{g1} \mid \mathbf{CP}) \times g1/\,g2$$

Because **g1** is fixed, it rotates backwards relative to the **CP** at 1 rotation per year. So:

$$\omega(\mathbf{g1} \mid \mathbf{CP}) = -1$$

$$\omega(\mathbf{g2} \mid \mathbf{CP}) = g1/\,g2$$

Because **g3** is fixed on the same arbor as **g2**:

$$\omega(\mathbf{g3} \mid \mathbf{CP}) = \omega(\mathbf{g2} \mid \mathbf{CP}) = g1/\,g2$$

Because **g4** is an idler gear:

$$\omega(\mathbf{g5} \mid \mathbf{CP}) = \omega(\mathbf{g3} \mid \mathbf{CP}) \times g3/\,g5 = g1/\,g2 \times g3/\,g4 = (g1 \times g3)/(g2 \times g4) = \Pi\,/\,\mathbf{Y}$$

The vector from the centre of **g5** to the **pin** will be called **red**. The vector from the centre of **g6** to the **pin** will be called **turquoise**. The vector from the centre of **g6** to the centre of **g5** will be called **black**. Then:

turquoise = **black** + **red**

turquoise' is the mirror reflection of **turquoise** in the **turquoise mirror. red'** is the mirror reflection of **red** in the **red mirror**. **black'** is the vector joining **pin'** to **pin''**. By the definitions of the mirrors, **black'** is parallel to **black** and is of the same magnitude, which is the "offset":

turquoise' = **red'** + **black'**

Because **red'** is the mirror image of **red** in the **red mirror**:

$$\omega(\mathbf{red'} \mid \mathbf{CP}) = -\,\omega(\mathbf{red} \mid \mathbf{CP}) = -\,\omega(\mathbf{g5} \mid \mathbf{CP}) = -\,\Pi\,/\,\mathbf{Y}$$

Using the basic law of relative rotations:

$$\omega(\mathbf{red'}) = \omega(\mathbf{red'} \mid \mathbf{CP}) + \omega(\mathbf{CP}) = 1 - \Pi\,/\,\mathbf{Y} = 1/r'$$

ηSince **black** is fixed to the Circular Plate and **black'** is parallel to **black**:

$$\omega(\mathbf{black'}) = \omega(\mathbf{black}) = 1$$

So, the output vector, **turquoise'**, fixed to **g7** and its tube, is the sum of a vector, **red'**, rotating uniformly at the calculated rate 1/r' of the planet round the Sun and a vector, **black'**, rotating uniformly in the same sense at the annual rate of the Sun. The length of **black'** is the Offset—correct for epicyclic theory. So, the mechanism calculates the ancient Greek epicyclic theory of the superior planet within the accuracy of the period relation.

**Inferior Planets**			
Planet	*Distance from Sun in AU, p*	*Centre to final epicycle in mm, i*	*Pin distance in mm, d*
Mercury	0.39	36.0	14.04
Venus	0.72	27.8	20.01
**Superior Planets**			
Planet	*Distance from Sun, AU*	*Offset: pin gear to slot gear in mm, o*	*Pin distance in mm, d*
Mars	1.52	6.58	10.00
Jupiter	5.20	1.58	8.22
Saturn	9.58	1.50	14.37

Supplementary Table S9. Geometric parameters for planetary gear trains.

By previous theory[21]: for inferior planets, d = p x i; for superior planets, d = p x o. Parameters are calculated using modern theory for the planets, but could equally be calculated from ancient Greek parameters, using the determination of epicycle sizes described earlier.

5. Supplementary Discussion S5: Matching the Evidence

5.1 Fragment A

The primary evidence comes from Fragment A—Supplementary Fig. S9. Previous publications[7,9] have named the spokes as the 1 o'clock, 4 o'clock, 7 o'clock and 10 o'clock spokes. Here for brevity we refer to these as A, B, C and D.

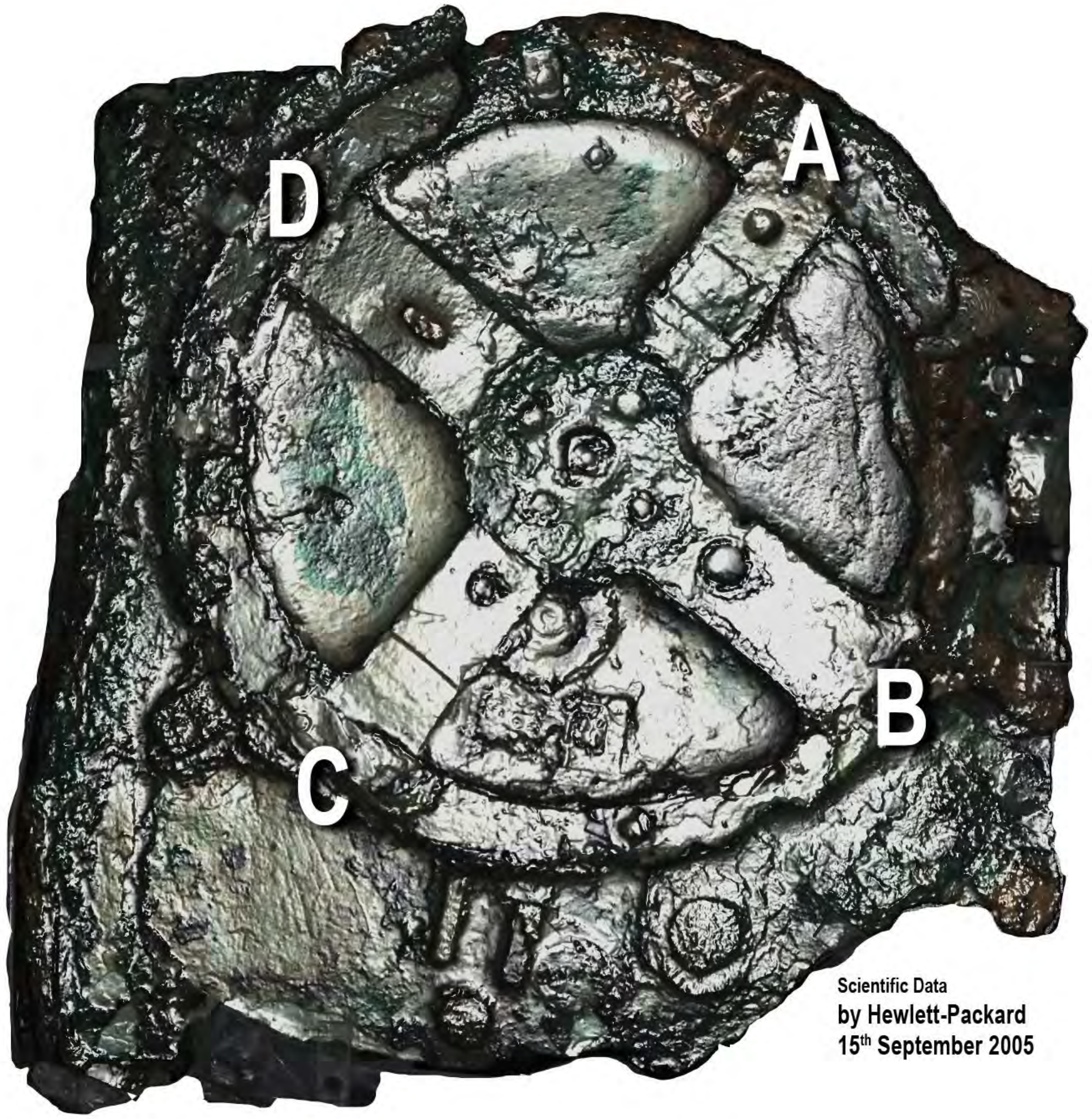

Supplementary Fig. S9. PTM of Fragment A, showing the Main Drive Wheel, **b1.** This PTM with specular enhancement highlights the bearings and other features on the spokes and the pillars on the periphery.

The physical evidence from the Main Drive Wheel **b1** in Fragment A suggests a complex epicyclic system mounted on this wheel, as was noted previously[2]:

> *This main drive wheel preserves clear evidence of some sort of superstructure mounted over it. The spoke in the ten o'clock position has a lug mounted on… The three other spokes contain holes indicating that they may also have had similar lugs on them and in addition there is a square depression on the spoke in the one o'clock position. Furthermore, on the rim, exactly midway between each of the spoke positions, there are traces of former fixtures… The evidence seems to suggest that pillars rising from these four places on the rim and another four on the spokes supported some sort of plate above and parallel with that of the drive wheel, turning with it.*

Following a subsequent publication[3], we believe that the evidence implies that an extensive epicyclic system mounted on **b1** calculated the variable motions of the Sun and the planets and displayed their ecliptic longitudes in the Zodiac. In addition, we propose a *hypothetical* mechanism for the *Line of Nodes* of the lunar orbit.

5.2 The Pillars on the Main Drive Wheel

a **b**

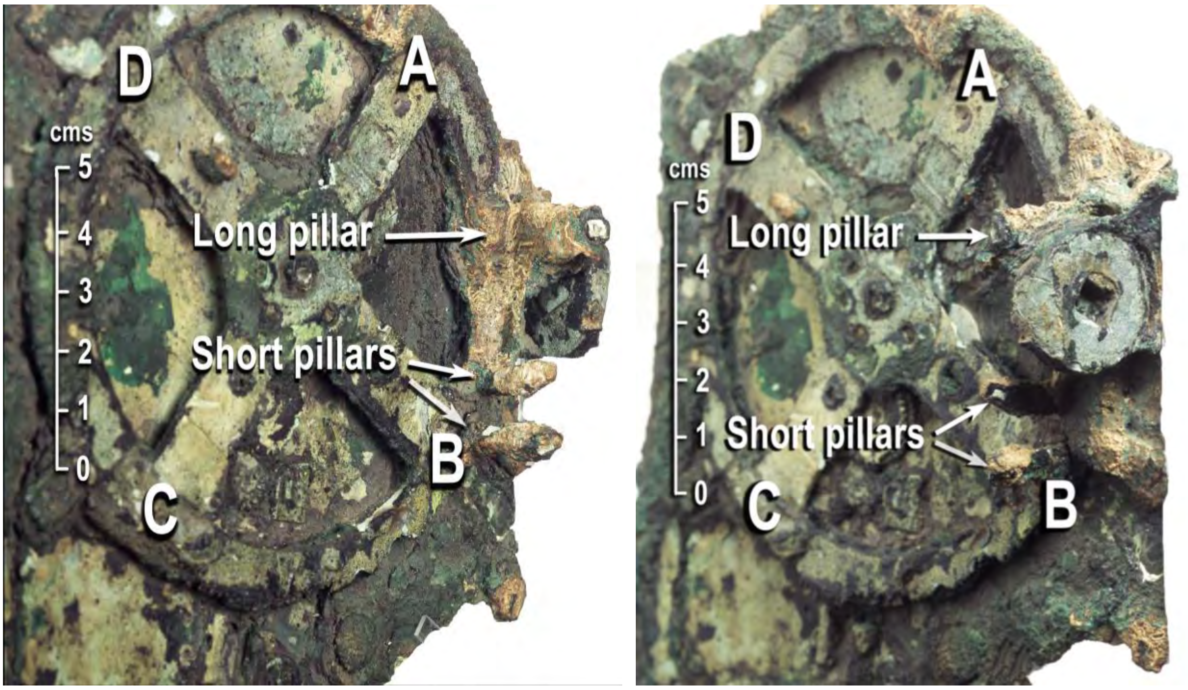

Supplementary Fig. S10. The pillars on the Main Drive Wheel. Photographs, showing one long pillar and two short pillars, which survive on the periphery of **b1**. The circular feature is the input contrate gear **a1**.

First, we examine the physical evidence. The pillars on the Main Drive Wheel **b1** are a striking feature of **b1**. We believe that they are an essential part of the support structure for the epicyclic system mounted on **b1**. All the images in Supplementary Fig. S11 are from an improved X-ray volume of Fragment A[22]. There are four surviving pillars—two long and two short. Though the other pillars have disappeared, there is clear evidence of attachments on the periphery of **b1**, which show that originally there were four short and four long pillars[9]. All the pillars have shoulders and pierced ends for retaining pins—meaning that they were intended to carry plates[9]. The short pillars are arranged near Spokes B and D, though not aligned with the spokes. The four long pillars are evenly disposed at intervals of about 90° around **b1**, mounted on the rim halfway between each pair of crossings. We can therefore infer that there was a rectangular plate carried by the short pillars, the **Strap**, and a circular plate carried by the long pillars, the **Circular Plate** (**CP**) (Fig. 4). The **Strap** and the **CP** must have had holes in the middle to enable the output tubes from the gearing to reach the front dials.

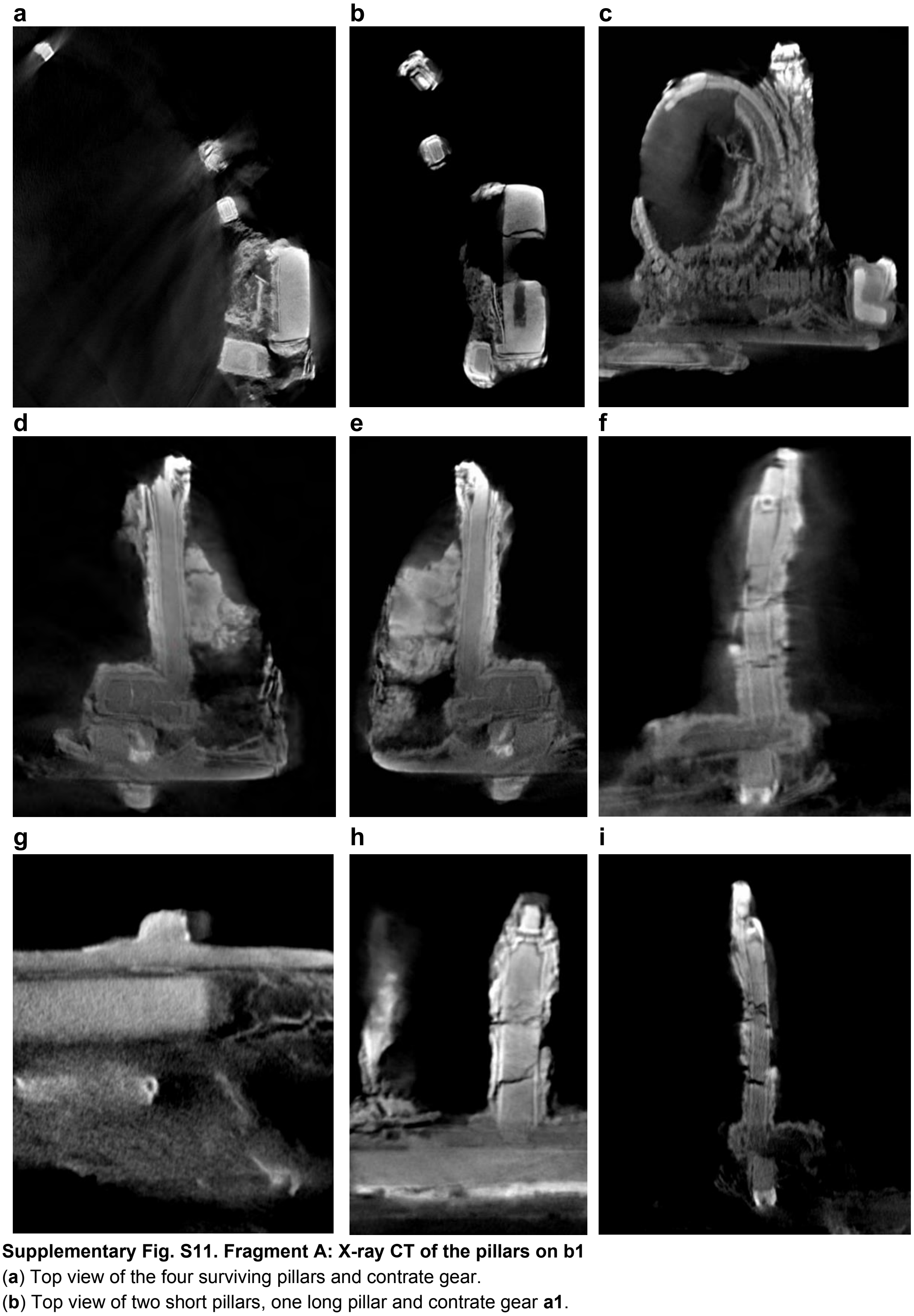

Supplementary Fig. S11. Fragment A: X-ray CT of the pillars on b1
(**a**) Top view of the four surviving pillars and contrate gear.
(**b**) Top view of two short pillars, one long pillar and contrate gear **a1**.
(**c**) Long pillar and contrate gear, **a1**.
(**d**-**f**), Long pillar.
(**g**) Remnant of long pillar, previously overlooked.
(**h**), (**i**) Short pillars.

This evidence defines a framework for our epicyclic system (Fig. 4m-o), which must have been part of the original Antikythera Mechanism. **b1** has four spokes, suggesting four

functions, with each spoke serving a different function. Our model does exactly this, as well as showing in detail how the features match our proposed mechanisms.

5.3 The Spokes on the Main Drive Wheel

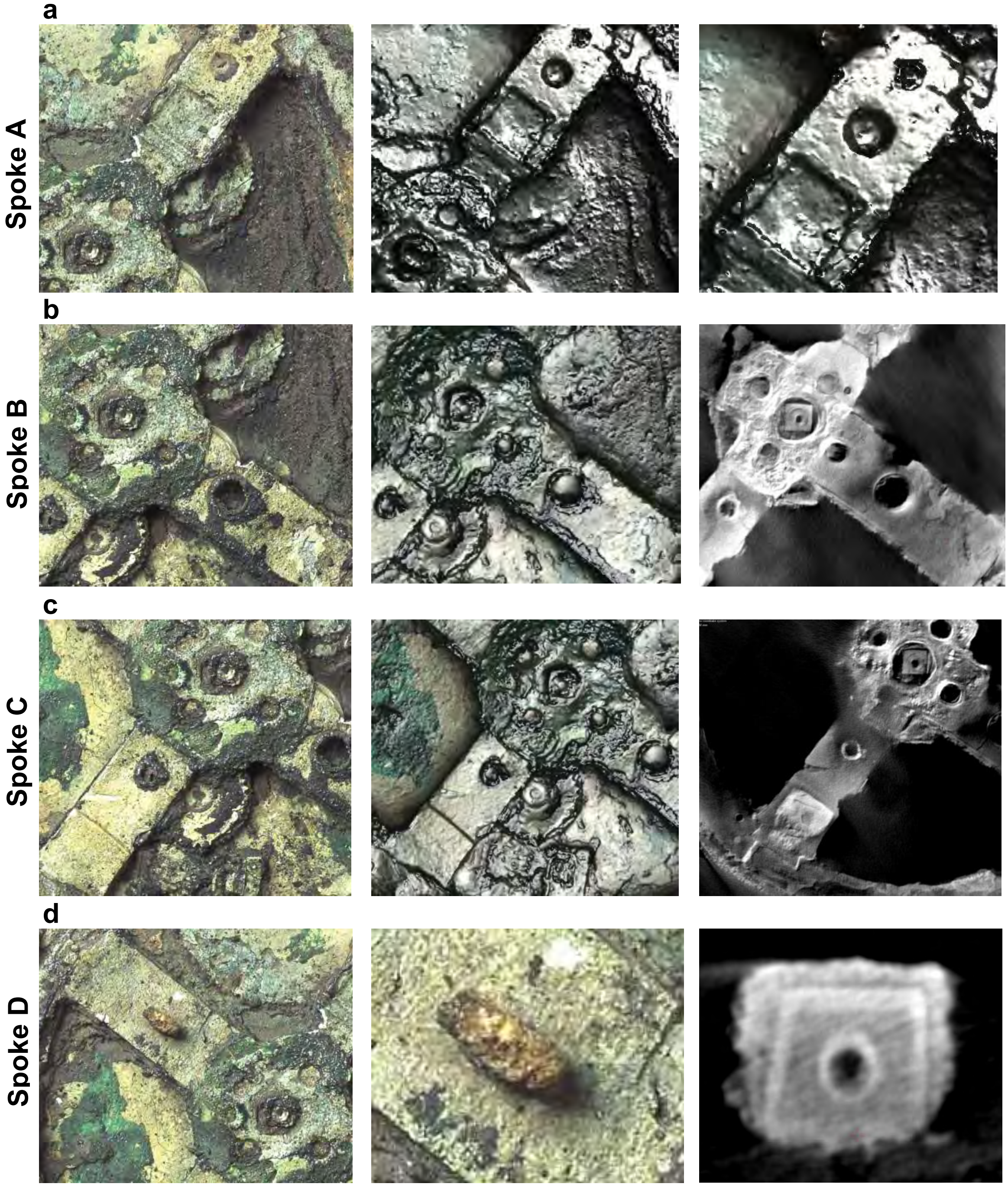

Supplementary Fig. S12. Features on the spokes of b1.

(**a**) **Spoke A**: Rivet that attaches the spoke to the central hub. Depressed flat area—width 10.5 mm, length 10.3 mm, depth 1.1 mm, centre 31.6 mm from central axis. Circular indentation—41.4 mm from the central axis.

(**b**) **Spoke B**: Rivet that attaches the spoke to the central hub. Circular hole—27.0 mm from the central axis.

(**c**) **Spoke C**: Rivet that attaches the spoke to the central hub. Circular hole—25.6 mm from the central axis. Raised flat area of higher X-ray density—width 14.2 mm, length 12.0 mm, height 1.1 mm, centre 46.2 mm from the central axis, including a rivet for possible attachment.

(**d**) **Spoke D**: Rivet that attaches the spoke to the central hub. Prominent block—centred 31.8 mm from the central axis; transverse X-ray CT slice shows pierced, tapered block—original dimensions height 5.0 mm, length at base 5.5 mm, tapering to 5.0 mm at top, depth 3.4 mm. Hole is 1.4 mm in diameter, centred 2.3 mm from bottom.

5.4 Fragment D

The components in Fragment D, shown in Supplementary Fig. S13, are crucial evidence.

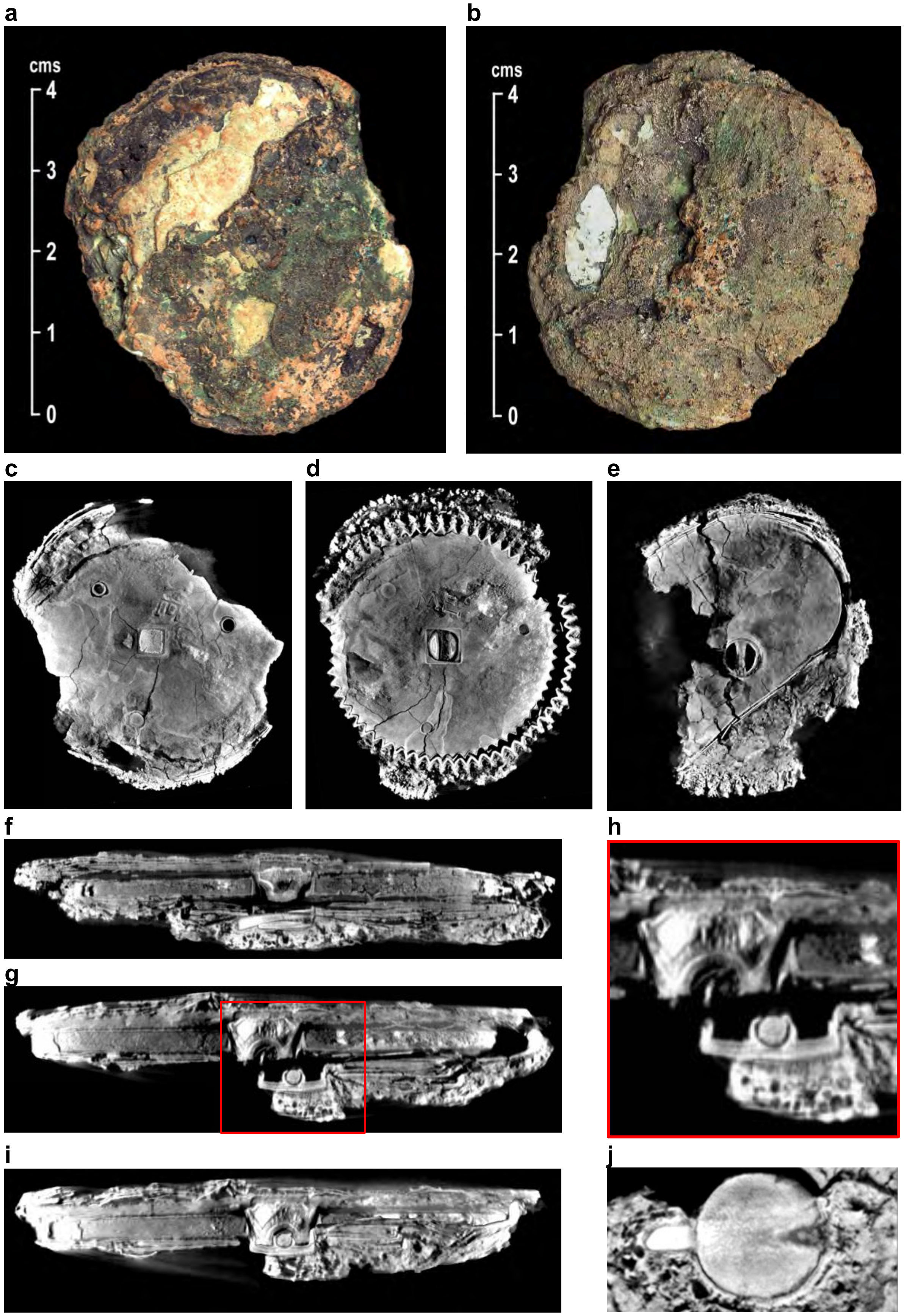

Supplementary Fig. S13. Fragment D: Photos & X-ray CT slices.
(**a**), (**b**) Photos of both sides of Fragment D. (**c**) X-ray slice of *disk*. (**d**) X-ray slice of *gear*. (**e**) X-ray slice of *D-plate*. (**f**), (**g**) Orthogonal slices through Fragment D. (**h**) Close-up of split hub. (**i**) X-ray slice in (**f**), restored using *Photoshop*. (**j**) Close-up of the end of the arbor.

There is evidently at least one gear in Fragment D (Supplementary Fig. S13d). Earlier studies[2,4,5] suggested that there are two gears in the fragment. As previously described[9], the illusion of two gears is caused by a strip of material that has parted from the periphery of the gear when the hub split. The split hub can be seen in Supplementary Fig. S13g, h. In Supplementary Fig. S13g it can be seen that the strip of detached gear teeth is connected to a thin section of plate that has separated from the body of the gear and in turn this is connected to the split hub. Using *Photoshop*, the gear teeth, thin plate and split hub can be moved to the left, upwards to a small extent, and slightly rotated clockwise—as in Supplementary Fig. S13i—then everything fits back together and the gear and arbor can be restored to their original configurations. Despite the appearance of two gears, there is definitely only one gear in Fragment D. The original tooth count can be reliably determined as 63 teeth, since all but three teeth survive[5,7,9].

The basic components of Fragment D are a *disk*, *gear* and *plate* (referred to here as the *D-plate*) and an *arbor* linking all three elements. These are reconstructed in Supplementary Fig. S14.

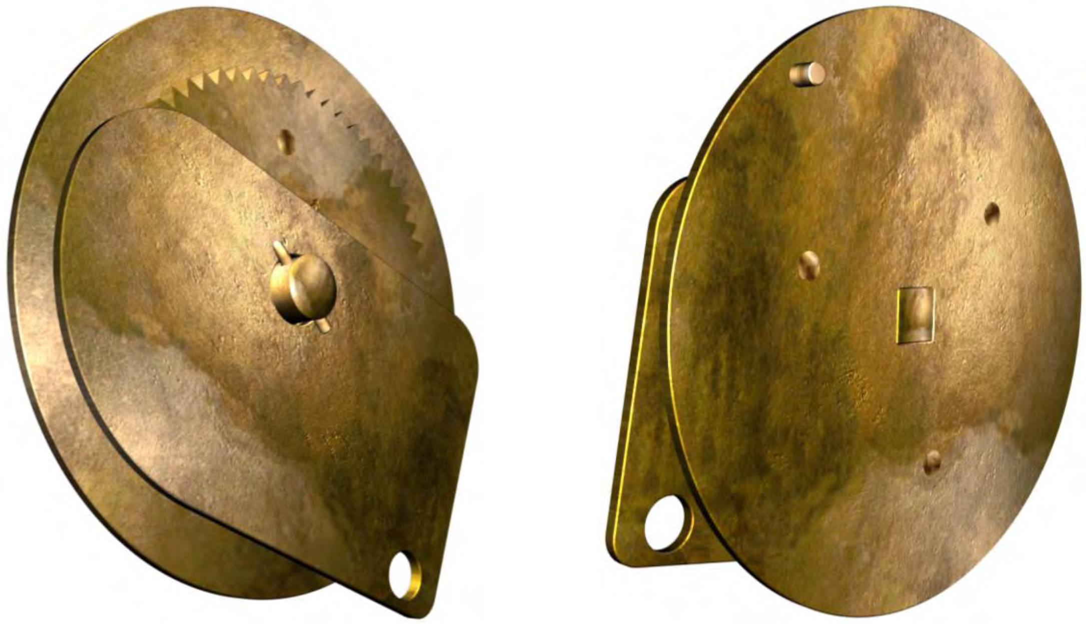

Supplementary Fig. S14. Reconstruction of the components in Fragment D. The components are reconstructed as part of the epicyclic mechanism that turns Venus. The disk is rivetted to **63** and has a conjectural *pin* attached. A *follower*, pivoting on the central axis, follows the pin to create the variable motion of Venus. The assembly turns in a bearing in the *D-plate*, which is reconstructed to include a bearing for an idler gear **26**.

The disk and gear are riveted together and have square holes at their centre matching squared sections on one end of the arbor. This end of the arbor is smaller than the round central section, so the disk and gear must have been mounted from this end. The arbor ends smoothly and flush with the disk so the likelihood is that the disk and gear were riveted together, slid onto their arbor, and then the end of the arbor peened to rivet these components in place permanently before being filed flush and tidied up. This was necessary so that the slotted follower of our Venus mechanism, which generates the variable motion, could run smoothly over the face of the disk. The arbor, disk and gear clearly all turned together. Inside the thickness of the gear, the arbor changes shape from square to round, so that it is purely round where it emerges on the other side of the gear. At this point there is the plate, whose hole is circular, and after that there is a cross hole through the arbor where a pin would have held the plate in place. The X-rays show that the arbor has split, but the tip is still present, just displaced sideways, and is nicely finished in a shallow dome (Supplementary Fig. S13g-j), so the arbor has not broken off. Careful measurement of the arbor from square end to pin hole, and the thicknesses of the disk, gear and plate, establish that there is no space for any other component or bearing on this arbor. Therefore, it is the arbor that pivoted in the plate, and not that this whole assembly pivoted in something else.

The parameters of the components are as follows:

D-Plate: Width 25.3 mm. Thickness: 1.0 mm

Gear: Teeth 63. Inner radius 15.9 mm. Outer radius 16.9 mm. Thickness 1.5 mm

Disk: Radius 21.5 mm. Thickness 1.0 mm.

Note that all dimensions are approximate because the components are highly corroded and covered in accretion layers but are probably accurate to a few tenths of a millimetre.

The D-plate is not symmetrical relative to the arbor, with the X-ray CT showing that the straight edge at the bottom extends about 3 mm further to the left than to the right (Supplementary Fig. S13e). The form of the missing part of the D-plate is not determined by the evidence. We reconstruct it as asymmetrical, so that the missing part can serve as a spacer to bring the epicyclic components to the correct level in the output hierarchy; to aid their smooth-running; and as a bearing for an idler gear **26** in the Venus train. Alternatively, the D-plate might have been nearly symmetrical, with a separate spacer and bearing for the idler gear.

No other surviving gear in the Mechanism has a disk attached. In an inferior planet mechanism, the pin-and slotted follower requires a pin that turns with the epicyclic gear but is beyond the edge of the gear[21]. The attached disk is the right size to carry the pin at the correct distance from the centre of the gear for a pin-and slotted follower for Venus. It is difficult to see what other role this could have played, except as the epicycle for Venus. Based on the X-ray CT data, it was previously proposed that the gear and disk in Fragment D might be the epicyclic elements of a Venus mechanism[9]. Even before the X-ray CT data were gathered, a similar solution with a disk carrying a pin was previously published[3]. The structure of the gear and disk in Fragment D, riveted together, strongly evokes this previous proposal. The width of the *D-Plate* is commensurate with the assumed width of the **Strap**, based on the separation of the short pillars.

Here we describe the chain of reasoning that leads to unique reconstructions of the Venus and Mercury gear trains. A general principle is that in the surviving gears the larger prime factors of the tooth counts *all* have astronomical meaning, derived from astronomical cycles that are the basis of the Mechanism; the smaller primes mostly have geometric functions.

Venus is an inferior planet and we mechanize it using our theoretical gear train: **g1** ~ **g2** + **g3** ~ **g4 ~ g5** ⊕ **follower** (Fig. 3c). The period relation will be multiplied by an integer multiplier to make viable gears. The period relation for Venus is (**289**, **462**) = ($\mathbf{17^2}$, **2** x **3** x **7** x **11**). **g2** and **g5** carry prime factors of the number of years in the period relation; **g1** and **g3** for the number of synodic cycles. For Venus, the number of synodic cycles is $\mathbf{17^2}$, so the fixed gear **g1** as well as **g3** must have a multiple of **17** teeth. This leaves realistic choices for **g1** of **17**, **34**, **51** or **68** teeth. Working through the options, it is easy to establish that, except for **51**, all the other choices lead to gears with over 100 teeth, which are too large. This means that the integer multiplier for Venus must be divisible by **3**.

g2 must carry the prime factors **2** and **11** and it must match a suitable bearing on one of the spokes of **b1**. This leads to a unique solution: it must be **44** and it must turn in the bearing on Spoke C, which is in exactly the right place. **g3** can only be **34** = **2** x **17** in order to calculate the period relation. The number of teeth on the idler gear **26** is chosen for geometric reasons.

Recall from Supplementary Discussion S4 that the *module* of a gear is the ratio, *pitch diameter / number of teeth*. In order for the arbor of **g2** + **g3** to exactly coincide with the bearing on Spoke C, this choice requires a small modification of the module of **g1** to around 0.53 and **g2** to around 0.54—well within the range of modules of the surviving gears (Supplementary Table S8). It also requires that the integer multiplier for Venus must be divisible by **2**. Because **3** is a prime factor of the multiplier, it must be divisible by **6**. It can only be **6**, since another prime factor would mean that it was at least **12**, making some gears far too large. The module of **63** is 0.521 (Supplementary Table S8) and we use the same module for the meshing gears **26** and **34**.

This means that **g5** must carry the prime factors **3** x **7** as well as the factor **3** from the multiplier: in other words, it must be **3** x **7** x **3** = **63**, which is the tooth count of the gear in Fragment D. This is the astronomical meaning of **63**. Now we have the whole gear train:

Venus: **51** ~ **44** + **34** ~ **26** ~ **63** ⊕ **follower**

The 3D disposition of the gears is determined by the fixed gear **51**, adjacent to **b1**, and the position of Venus in the cosmological ordering as the last of the outputs between **b1** and the **Strap**. To conform to this ordering, the arbor of **44** + **34** must bring the gear **34** forwards to the correct plane just behind the **Strap**. A bracket supporting this extended arbor exactly matches the flattened area on Spoke C (Fig. 4g, Supplementary Fig. S12). The idler gear **26** and the final epicyclic gear **63** turn in the D-plate, which is soldered to the **Strap** (Supplementary Fig. S15 shows evidence of soldering in the Antikythera Mechanism). The X-ray CT reveals that the *disk* is rivetted to **63** and is exactly the right diameter to carry a pin for Venus, tracked by a follower turning on the central axis, with a tube attached, carrying the anomalous rotation of Venus to the *Venus ring* on the front display (Fig. 5c).

There are no surviving gears in the Mercury gear train. For economy, our reconstruction shares the fixed gear **51** with Venus and is reconstructed with the same architecture—so, we have chosen a period relation for Mercury that includes the prime factor **17** in the number of synodic cycles. We mechanize Mercury with the same type of 5-gear train as for Venus: **g1** ~ **h2** + **h3** ~ **h4** ~ **h5** ⊕ **follower** (Fig. 3c). The tooth counts of the rest of the gears are determined by the need to accommodate the large prime factor **89** in the number of synodic cycles. **89** must fit between the output tube system and the edge of **b1**. The prime factors of the period relation and the available space define the possibilities for **g2**. The period relation for Mercury is (**1513**, **480**) = (**17** x **89**, $\mathbf{2^5}$ x **3** x **5**). We have chosen an integer multiplier of **3** for the fixed gear **51**. For Mercury, the fact that **h3** is divisible by **89** and cannot be a multiple of this, means that the integer multiplier for the Mercury train must be **3**. The period relation then implies that **g2** x **g4** = **3** x $\mathbf{2^5}$ x **3** x **5**. **51** with a module of 0.53 (as for Venus) has a pitch radius of 13.52 mm. We assume that the arbor of **g2** + **g3** coincides with the centre of the flattened area on Spoke A, which is 31.6 mm from the centre. If we allow a clearance of 0.1 mm between **51** and **g2**, this leaves 31.6 - 13.52 - 0.1 = 17.98 mm for the pitch radius of this gear, implying a gear with (2 x 17.98)/0.5 = 71.92 teeth, with a standard module of 0.5. Given the prime factors that must be included in its tooth count, the second gear of the Mercury train must have had **72** teeth (Supplementary Fig. S12a). The idler gear **40** is chosen to fit the geometry. The whole gear train is now determined as:

Mercury: **51** ~ **72** + **89** ~ **40** ~ **20** ⊕ **follower.**

The **Strap** is inclined to the spokes at just the right angle of 11° to accommodate the epicyclic gears for Mercury and Venus—explaining the angle of the short pillars relative to **b1**. For the first time, the features on **b1** and the components of Fragment D are explained (Figs. 4, 5, Supplementary Figs. S13, S14, S21, Supplementary Discussion S5, Supplementary Video S1).

5.5 Features of the surviving Mechanism

5.5.1 Evidence of soldering

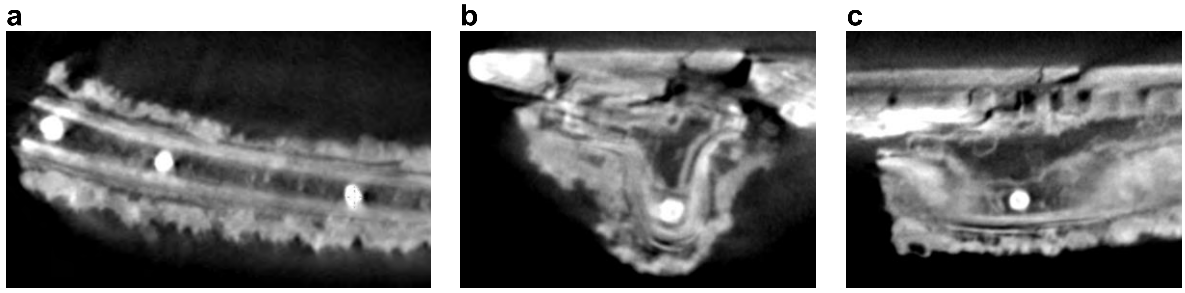

Supplementary Fig. S15. Evidence of soldering. Orthogonal X-ray CT slices of Fragment C, showing tiny beads of about 1 mm diameter with very high X-ray density. They are at the apex of a curved and profiled plate, which supports the Zodiac Scale.

There are no evident traces on the back of the D-plate, which suggest mechanical attachments: certainly, no rivets or lugs. We propose that the D-plate was soldered to the

Strap. There is evidence of soldering in the Mechanism: it has been persuasively argued that the tiny beads of very high X-ray density, seen in Supplementary Fig. S15d-f, are evidence of solder (Michael T. Wright: personal communication). They are at the apex of a curved and profiled plate that supports the Zodiac scale. This plate is attached to the back of the scale with no signs of mechanical attachments. We agree that these are solder beads, which are a frequent by-product of soldering, since there is no other obvious way that the supporting plate could have been attached to the scale and the beads are of very high X-ray density, suggesting lead content. In the X-ray CT, the clean appearance of the side of the D-plate, which is inscribed with the characters "ME", contrasts with the disordered appearance of the opposite side. This agrees with the idea that it was this side of the plate that was soldered to the Mechanism. In our model this side of the D-plate is soldered to the **Strap**.

5.5.2 Spacers and closely-packed gears

Spacers and closely-packed gears are conspicuous features of the surviving Mechanism, as illustrated in Supplementary Fig. S16. In many places in the Mechanism, spacers separate the gears and other mobile elements from direct contact with the plates. Sometimes, these spacers are riveted to the plates, as are three of the five spacers that separate **b1** from the Main Plate (Supplementary Fig. S16a, b). Two of the spacers do not appear to be fixed with rivets and were probably soldered. The four brackets, rivetted to the Main Plate, prevent **b1** from rocking on its arbor: the gear **b1** was held down by the brackets so that it would slide on the five spacers and maintain its orientation. Both spacers and brackets suggest an inability to make bearings and arbors accurately enough to prevent unwanted lateral gear movements, particularly for larger gears.

Similarly, there are other spacers with the same function. Supplementary Fig. S16c shows an X-ray CT slice through a horse-shoe-shaped spacer on axis **d**, which separates **d2** from the Main Plate. There is no evidence of mechanical fixings and it is most likely that it was attached to the Main Plate with solder. In Supplementary Fig. S16d there is a photograph of the back of the Moon phase device in Fragment C. It has been argued convincingly[10] that the part-circular feature around the central axis is evidence that there was a spacer attached here, which stabilized the Moon phase cap and separated it from the next output—the Dragon Hand in our model. The surviving part-circular feature is less than half a millimetre thick—far too thin to be the spacer itself. It is also higher in X-ray density than the surrounding plate. It is evidence of where the spacer (now lost) was originally attached: solder appears to be the only way that the spacer could have been fixed to the back of the Moon phase cylinder. Residual traces of solder would explain the high X-ray density. In Supplementary Fig. S16e there is a cross-section X-ray CT slice through Fragment A. The positions of the gears are indicated in Supplementary Fig. S16f. In the Antikythera Mechanism the gears and spacers throughout the Mechanism had their faces in contact with each other: they did not follow later universal practice, where gears would be separated by air spaces for friction reasons. In Supplementary Fig. S16e, f there are five layers of gears below the Main Plate, which are packed into a depth of about 8.0 mm. We will follow a similar close-packed design when we reconstruct the Cosmos gearing—satisfying *Reconstruction Principles 1: Utilise and conform to all the surviving evidence*; and *3. Match to contemporary astronomy and technology.*

5.5.3 Future research

For future study, we will be examining the engineering issues involved in the hand-manufacture of the gears and arbors. Another focus will be on metallurgical issues such as work hardening and the consequent metallic microstructures. We will also study the parameters of such closely-packed gearing systems, with obvious issues such as friction and lubrication. If we can show that the manufacturing methodology meant that such gearing systems could be made with comparative ease, this would have consequences as to whether the Antikythera Mechanism was as unique as sometimes believed.

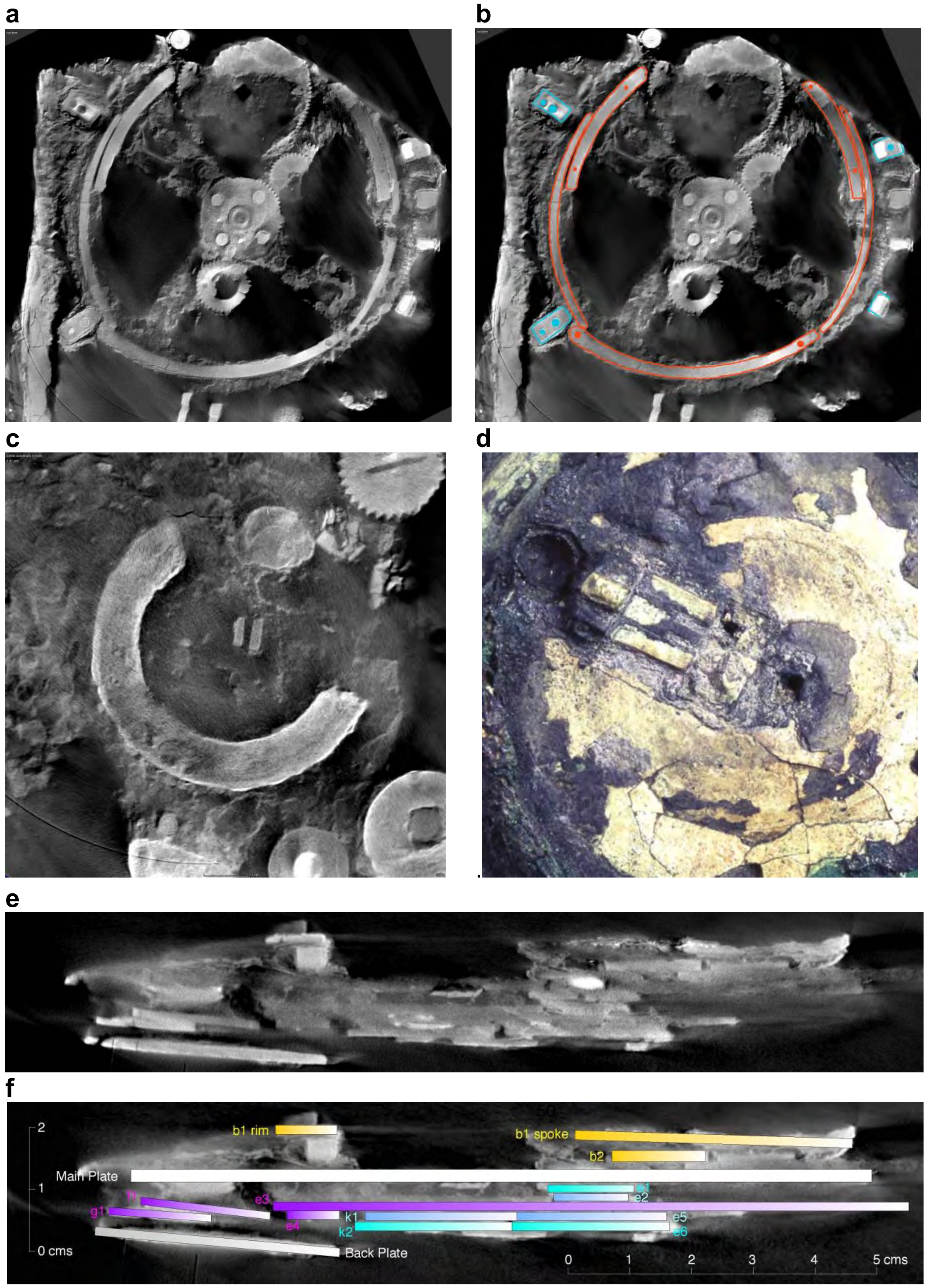

Supplementary Fig. S16. Spacers & Closely-Spaced Gears. (**a**) X-ray CT slice of Fragment A, showing four brackets and five bronze-strip spacers behind **b1**. (**b**) The brackets are traced in blue. They are rivetted to the Main Plate. The tops of the brackets hold down the periphery of **b1**. The spacers are traced in red. Three of them are rivetted to the Main Plate; the thinner spacers to the left and right do not appear to have rivets and were probably soldered. (**c**) X-ray CT slice of Fragment A, showing a part-circular spacer on axis **d**, attached to the back of the Main Plate. There is no evidence of rivets. (**d**) Photograph of the back of Fragment C, showing traces where a part-circular spacer was attached to the back of the Moon phase device. (**e**) Cross-section X-ray CT slice through Fragment A, showing tightly-packed gears. (**f**) Cross-section with gear positions superimposed. *(**E**)-(**F**) reproduced with permission*[9].

6. Supplementary Discussion S6: Reconstructing the Cosmos

6.1 Historical Research on the Cosmos in the Antikythera Mechanism

There has been a history of more than a hundred years of proposals for the architecture of a gearing system to mechanize the motions of the Cosmos at the front of the Antikythera Mechanism.

6.1.1 Earliest Planetarium Model

After its discovery in 1901, there was intense interest in the Antikythera Mechanism, followed by much controversy but little real understanding—though there were hints from the inscriptions that it was astronomical in nature and might be some sort of calculator[56]. More than a hundred years of research has led to great progress. The proposed mechanical Cosmos described here has drawn on many ideas from this previous research. The idea that it included a *Planetarium* goes back to the early days of research in the period 1905–06[1], when a German philologist, Albert Rehm, first gained real insights into the true nature of the Antikythera Mechanism. He identified a *parapegma*, an ancient Greek star almanac, on the front plate of the device and he started to try to understand how the Mechanism worked.

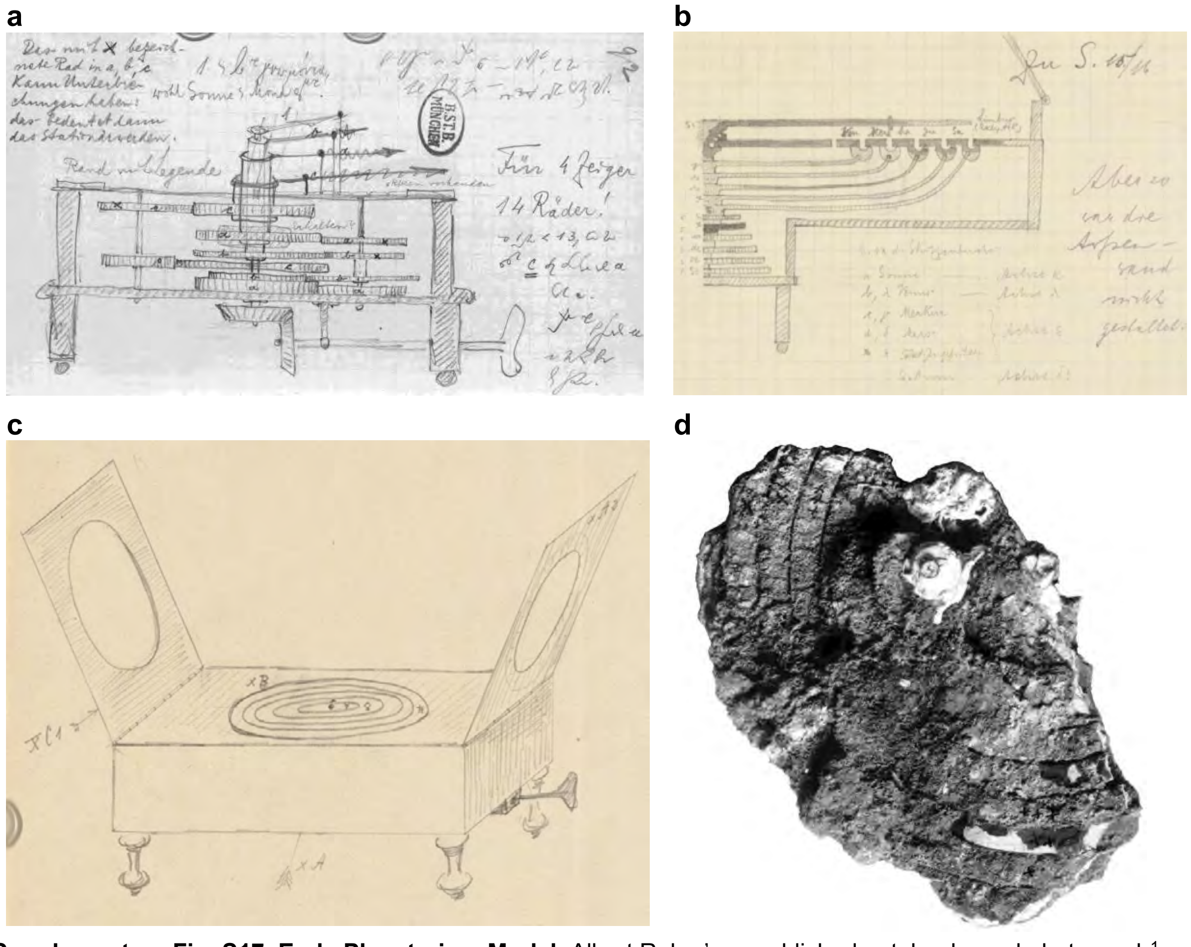

Supplementary Fig. S17. Early Planetarium Model. Albert Rehm's unpublished notebooks and photograph[1], 1905 – 06.

(**a**) Coaxial pointer display.

(**b**) Proposal for coaxial ring system.

(**c**) Ring display.

(**d**) The back of Fragment B. *For image credits, see information at the end of this document.*

In his unpublished notes, Rehm called his early model of a planetarium for the Antikythera Mechanism, *Mein Planetarium*[1]. He proposed a coaxial system of outputs and, in one version, a coaxial ring display. He also suggested that the device included epicyclic gearing—an extraordinary idea for ancient Greece. He believed wrongly that the five rings evident in Fragment B (Supplementary Fig. S17d) represented the five planets known in the ancient world: we now know that these rings (Supplementary Fig. S17d) are fixed and are

part of a five-turn 19-year Metonic Calendar[4,7]. Diagrams in these research notes (Supplementary Fig. S17b, c) show a coaxial system designed to turn a set of planetary rings and we will use this ring design in our output display for the Cosmos. He listed the astronomical bodies in an unusual order, *Sun*, *Venus*, *Mercury*, *Mars*, *Jupiter*, *Saturn*, which we know from the inscriptions is not correct for the *CCO* of the Antikythera Mechanism. Rehm did not have access to these inscriptions, which were only revealed by the X-ray CT[7,8,9]. Due to lack of good data, he got nearly everything mechanically wrong, but his ideas were highly prescient. We believe that his *Planetarium*, with rings for the planets, and his *epicyclic gearing* were correct and very much in advance of their time: our model realizes and enhances the vision of Albert Rehm in 1905–06.

6.1.2 Gears from the Greeks

The next relevant phase of research was from the early-1950s to the mid-1970s, culminating in Derek de Solla Price's seminal monograph, *Gears from the Greeks* (1974)[2]:

> *…there is a possibility that this space between the large wheels may have held a gearing system, now totally vanished, which served to exhibit the rotations of all of the planets other than the Sun and Moon.*

He made no attempt to reconstruct the proposed planetarium and we now know that the second of the *"large wheels"* did not exist. However, our research is concerned with taking up this proposal by recreating the lost gearing in front of the Main Drive Wheel, **b1**, which we are confident calculated the anomalous motions of the planets, probably also of the Sun and possibly the *Line of Nodes* of the Moon. Price's research wrongly suggested that inscriptions on the rings in Fragment B describe the synodic phases of the planets[57].

> *On the upper dial the inscriptions are much more crowded and might well present information on the risings and settings, stations and retrogradations of the planets known to the Greeks (Mercury, Venus, Mars, Jupiter and Saturn).*

We now know that these inscriptions are in fact month names and year numbers in a Metonic Calendar[8] and it is the *Front Cover Inscription* (FCI) that describes the synodic phases of the planets[9,12]—exactly the same idea but in a completely different place.

6.1.3 First Solid Planetarium Reconstruction

A model, proposed by Michael Wright in 2002, is shown in Supplementary Fig. S18.

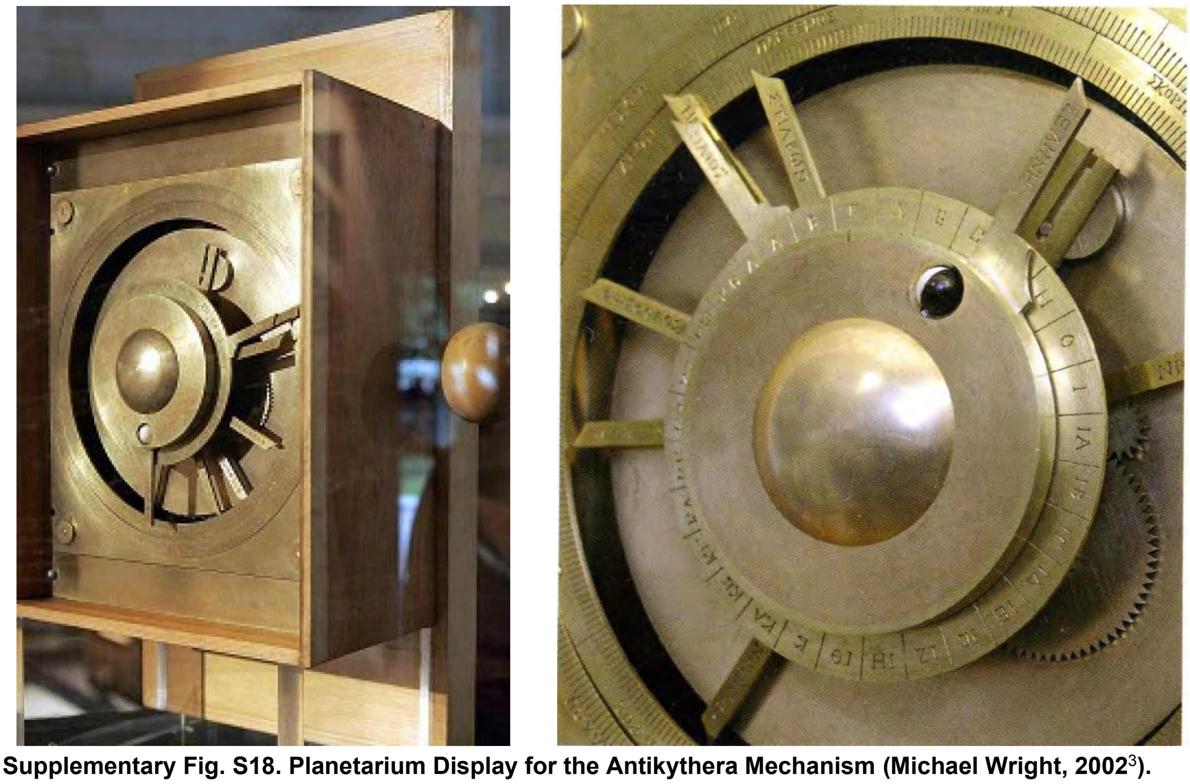

Supplementary Fig. S18. Planetarium Display for the Antikythera Mechanism (Michael Wright, 2002[3]).

From the 1980s onwards, there was a sustained critique of Price's classic research, which corrected a substantial portion of the gearing[4]. A very important advance came in 2002 with the publication of a paper by Michael Wright, *"A Planetarium Display for the Antikythera Mechanism"*[3]. For the first time, a workable model for the planets was created. This radical and surprising proposal conjectured that there was a co-axial system of eight pointers at the front, which indicated the Date, Sun, Moon and all five planets known in the ancient world. In an ingenious attempt to counteract the severe parallax inherent in such a system, some of the pointers lie behind the Zodiac Scale. A complete gearing system was constructed to turn these pointers—based on the ancient Greek epicyclic theories of the planets. It was mechanized using *pin-and-slotted followers* to display the variable motions of the planets and compound gearing to calculate accurate cycles for the planets. This basic architecture of coaxial outputs has now achieved a high degree of acceptance, though the structure of this proposal has been subject to extensive criticisms and revisions[9] and proposals for a very different gearing system and output display—as we describe here. Without this pioneering model, none of the subsequent proposals for planetary outputs would have been possible.

For each of the solar and planetary mechanisms, there is a fixed gear and a second input gear rotating at 1 revolution per year: this *mean solar rotation* is carried by the *Main Drive Wheel* **b1** and its attachments such as the **CP**[9]. These inputs largely define the structural options for the planetary mechanisms. There are several competing architectures to determine how the individual mechanisms slot together to create the Cosmos output. In Wright's model[3], the mechanisms for the Sun and inferior planets have their fixed gears attached to the central fixed arbor, with its square hub. The mean Sun input comes from **b1**. For the inferior planets, the compound gear trains proposed in this model include gears whose arbors are centred in the empty spaces between spokes—so support structures must be constructed, which do not match the remains of bearings and fittings on **b1** (Supplementary Discussion S5). If this were the case, we would expect **b1** to be made as a simple disk, rather than the elaborate dovetailed spoke structure that we find. For the superior planets in Wright's model, each planetary mechanism must have its own module, because the system uses direct mechanisms (Supplementary Discussion S4). Without being on separate plates, the output followers would interfere with each other. The fixed input comes from the attachment of the module to the side casing of the Mechanism; the mean Sun input comes from an ancillary arbor with a bearing on the Main Plate. The arbor is driven by **b1** and each module has a gear the same size as **b1** to input the mean Sun rotation to the module. In our view, this is an awkward arrangement, which does not agree with the evidence—in particular, this model does not respect the evidence of the pillars on **b1** and their attached plates. Since this model was published, the structure of the *Parapegma* has been solved[12], showing that there were two Parapegma plates, attached to the Front Plate of the Mechanism, each plate being divided into two columns of inscriptions. Each column corresponds to the adjacent quarter of the Zodiac Dial. In Wright's *Planetarium* model, there is not enough space for these plates, so the form of the external case cannot be correct. This could be solved by making the whole of the front of the device flat—extending the case so that the top and bottom parts of the front plate match the level of the dial plate—though this would result in a very deep case.

6.1.4 Nonuniform Zodiac Scale & Subsidiary Dials for Synodic Cycles of the Planets

A later architecture for planetary displays by James Evans, Christián Carman and Alan Thorndike[58] takes a very different approach. Their research judged that the scale divisions on the Zodiac dial were systematically uneven. This research[58], and a more recent publication[59], propose that the nonuniform divisions were set out to show the solar anomaly according to *Babylonian System A*[15]. The major problem with this theory is that the lunar output is also displayed on the same Zodiac scale: its broken-off arbor is visible in the centre of **b1**[7] and the lunar output must have been shown by a pointer attached to this central arbor. The complex and subtle 17-gear system (including **b2**) for the lunar anomaly is designed so that the period of the lunar anomaly is the *anomalistic month* of 27.55 days—*not* the sidereal month of 27.32 days. Nonuniform scale divisions would introduce an additional lunar anomaly with a period of the mean sidereal month of 27.32 days. No known

lunar anomaly, either in ancient or modern times, has this period. A nonuniform scale does not make sense for the Moon and even less sense for the planets, if displayed on this scale.

The authors of the latest paper[59] write:

> *The mechanism's zodiac was also used for reading the longitude of the Moon—and probably the longitudes of the planets as well. Because of the nonuniform division of the zodiac, these bodies would at times appear to be displaced a bit… Perhaps the maker of the Antikythera mechanism believed that such an effect was real—i.e., that the Moon and planets share in this inequality with the Sun. Or—which seems more likely to us—perhaps this was an imperfection in the display that was simply accepted for the sake of a convenient representation of the solar anomaly.*

This does not answer the problems. Why would the designer believe that *"such an effect was real"*? Having taken so much trouble to mechanize the anomalies of the Moon and planets using complex period relations and compound epicyclic gear trains, it seems inconceivable that the designer would then have included the solar anomaly on a nonuniform scale that makes no sense for the other astronomical bodies that share this scale. In our model, the mechanization of the solar anomaly only takes one additional gear (because of shared gears in the system) and entails none of the compromises of a nonuniform scale. An earlier proposal by the same authors[58], obviates this problem for the planets. Their synodic cycles are displayed schematically on five subsidiary dials at the front of the Mechanism within the main Zodiac and Calendar dials. In our view, this system for the planets is not supported by the evidence. It does not conform to the description of the planetary display in the Back Cover Inscription (Supplementary Discussion S2). Nor does it agree with the descriptions from Cicero of contemporary mechanisms (Supplementary Discussion S2), which explicitly refer to the *motions* of the planets. In addition, a Babylonian System A scheme is surely not in accord with the design of the rest of the Mechanism. Though we do not support these proposals, the idea of *marking* the synodic cycles of the planets is used in our model—though in a different way. In summary, we do not believe that nonuniform divisions were made deliberately to express the solar anomaly: they were probably introduced accidentally during the process of making scale divisions on the Zodiac ring.

6.1.5 Introduction to the Cosmos in the Antikythera Mechanism

The research that started with *"A Planetarium Display for the Antikythera Mechanism"*[3] continued with a proposal[9] by Tony Freeth and Alexander Jones, visualized in Supplementary Fig. S19.

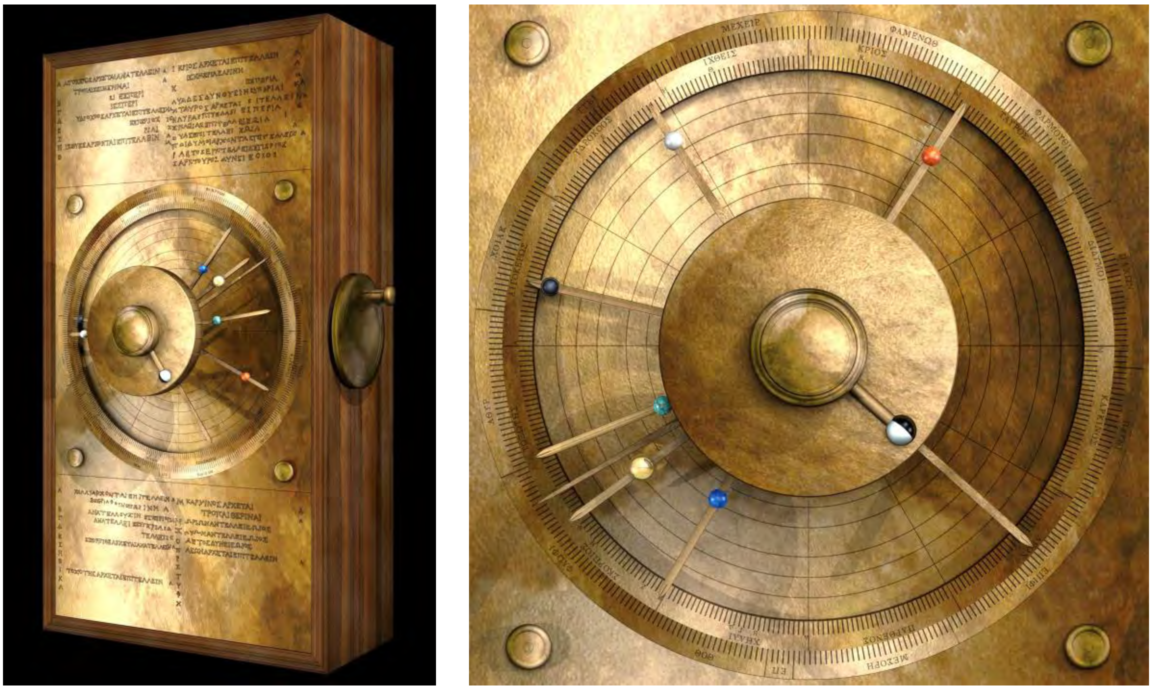

Supplementary Fig. S19. The Cosmos in the Antikythera Mechanism (Tony Freeth, 2012[9]). Computer model made in *Newtek Lightwave 3D*.

This scheme retained the co-axial system but simplified the gearing and questioned the structure of the planetary gearing. It used simple rather than compound gearing and, for the superior planets, was based on *indirect* gearing principles[11,9] (Supplementary Discussion S4), as exemplified in the known calculation by the Mechanism of the lunar anomaly[7]. This work also attempted to reconcile the proposed gearing with the evidence from Fragment A.

As discussed in the Main Text, the *Moon phase device*[10] at the front is a differential system that subtracts the solar rotation from the lunar rotation to produce the phase of the Moon—needing adjacent inputs from both the Moon and the Sun. These considerations caused this previous model[9] (Supplementary Fig. S19) to adopt a pointer system for displaying the ecliptic longitudes of the astronomical bodies, which carried *"little spheres"*, as described in the BCI, at appropriate distances from the centre to preserve the customary ancient Greek cosmology. This system was less than ideal from the parallax point of view and failed to fully match the description in the BCI.

This research also challenged the use of compound gearing for the planets on the grounds that the ancient Greek epicyclic theories were so inaccurate that more sophisticated planetary periods, realized with compound gearing, were not justified[21]—wrongly, as it turned out (Supplementary Discussion S3). In addition, very simple *indirect* superior planetary mechanisms were developed, which operated just like the known lunar anomaly gearing. These were discovered independently by two research groups[11,9].

6.1.6 The Planetarium of Archimedes

The descriptions by Cicero[2] of models made by Archimedes and Posidonios (Supplementary Discussion S2) are not specific enough to determine how the models looked or worked. They were referred to as *sphaerae*, which appears to imply a three-dimensional spherical form, though they have often been thought to refer to instruments like the Antikythera Mechanism, which have two-dimensional displays. Until recently, the form that three-dimensional models might take had only been imagined, but now a model has been built by Michael Wright, dubbed *The Planetarium of Archimedes*, which shows how such a model might have looked[60]. It was a highly original exercise and a very interesting contribution to the debate, though there is no direct evidence for such a device. We shall not discuss it here because such devices would come from a different mechanical tradition to that of the Antikythera Mechanism.

6.2 A New *Cosmos* for the Antikythera Mechanism

In Supplementary Fig. S20 a schematic gear diagram shows the gearing behind **b1** that was previously published[7,8,9].

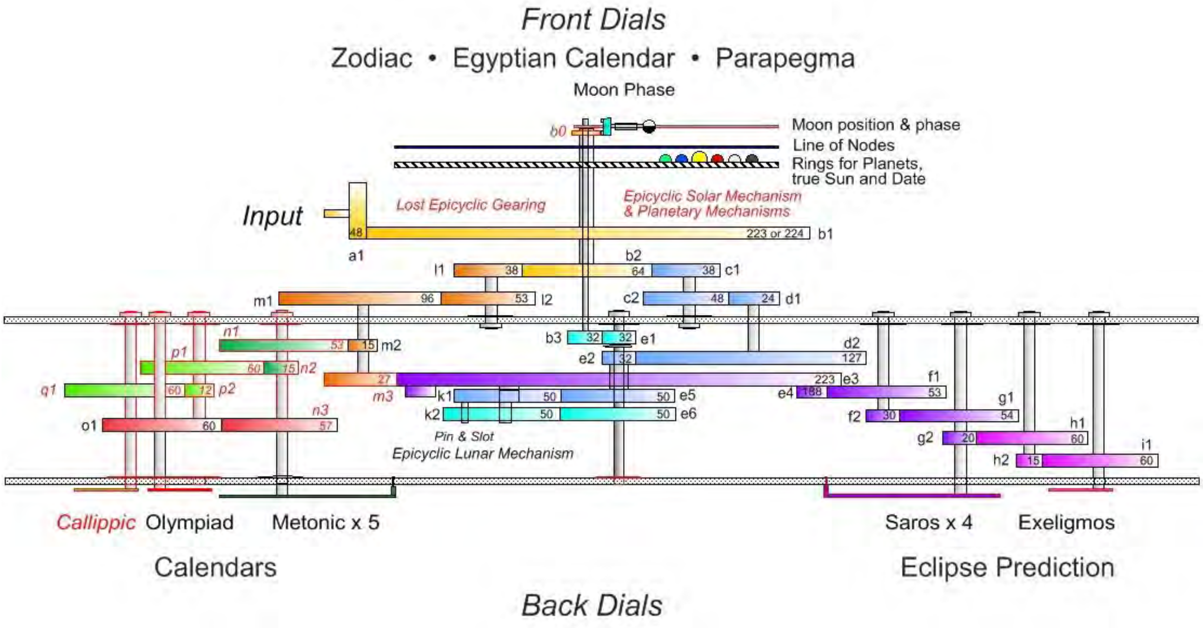

Supplementary Fig. S20. Gear Diagram (adapted from previous research[7]), showing the structure of the gearing behind b1. Gears in red are hypothetical and are necessary to make the gear systems work.

This diagram is now widely accepted, except for the gears that drive the lunar phase mechanism at the front, where there is still debate[61].

Gears in red are hypothetical gears that are essential to make the system work and to turn the lunar phase mechanism, the Metonic and Kallippic Dials and the Olympiad Dial. Our aim is to reconstruct the gearing in front of **b1**, where the diagram is marked *"Lost Epicyclic Gearing: Epicyclic Solar Mechanism & Planetary Mechanisms"* and to transform the Cosmos Display with a system of rings.

6.2.1 Sharing Gears

In Supplementary Fig. S20 it can be seen that the original mechanism extensively shared gears, where *astronomical primes* were shared between functions. For example, **l1** has 38 teeth, including the astronomical prime 19. This prime is needed in both the calculation of the rotation of the *Metonic pointer* and in the calculation of the rotation of the *Line of Apsides of the Moon*[7], which is realized on **e3**/**e4**. So, this one gear is shared between these functions. The large gear **e3** has 223 teeth, which is the key astronomical prime in the *Saros Cycle*. This prime factor is needed to turn the *Saros pointer* and it is also needed in calculating the rotation of the *Line of Apsides*. Rather than make two large gears with 223 teeth, the designer made just one gear, shared between two different functions. Using our *Reconstruction Principles 1* and *3*, we use shared gears extensively in our model to minimize the overall number of gears.

6.2.2 Framework for the Reconstruction

The framework for our model is defined by the pillars on **b1** and the attached plates (Fig. 4m-o). There are some uncertainties about the measurements of the pillars and hence the spaces available for gearing, though these uncertainties are small enough that they make no difference to the numbers of layers of gearing that can be accommodated. From earlier work[9], we estimate that the total length of the long pillars was 32.0 mm, with distance to the shoulder of 27.5 mm; the total length of the short pillars was 20.4 mm, with distance to the shoulder of 16.2 mm. We estimate that the thickness of the **Strap** would have been about 1.6 mm and of the **Circular Plate** about 2.0 mm. The thickness of the central disk (that links the spokes on **b1**) and its attachment rivets is 1.2 mm. From these estimated measurements, it is easy to calculate the available spaces for gearing: between **b1** and the **Strap** about 15.0 mm; and between the **Strap** and the **Circular Plate** about 9.7 mm. In our model, we have nine layers in the former space and five layers in the latter. The gearing systems need to be carefully interleaved, so that the outputs conform to the customary ancient Greek cosmological order. Some of the nine layers are thinner than others —1.0 mm for the planetary disks and the D-plate, as determined from the X-ray CT. The spaces defined by the pillars are commensurate with the number of layers in our model and the density of the layers matches the surviving Mechanism (Supplementary Fig. S16e, f). This explains the lengths of the short and long pillars and strongly supports the architecture of our design.

6.2.3 Output System

The outputs consist of a system of nested coaxial output tubes. There is a precedent for this sort of coaxial arrangement in the Mechanism itself in the *lunar anomaly mechanism*, which has a system of three coaxial mobiles on axis **e**[7]—so it conforms to our *Reconstruction Principles.* From an engineering perspective, the system of coaxial output tubes is the most problematic aspect of the present model. The sheer complexity of nine outputs on the same axis was surely challenging for ancient Greek technology. In the 2002 proposal, there were eight coaxial outputs (we have added a Dragon Hand). Although the elegance of this proposal is obvious, its manufacturability poses real challenges and therefore demanding questions. From a theoretical perspective, our initial scepticism about this radical proposal has now turned to confidence that it is essentially correct, since there does not appear to be any other viable option that is consistent with the evidence. Using modern techniques, this would normally be achieved by precision machining on a lathe, and centre drilling. In the future, we plan to do some experimental archaeology to establish its plausibility in the epoch of the Antikythera Mechanism. A particular focus of this study will be to make the nested output tubes, using tools and techniques that might have been available in ancient Greece.

There is a general principle that the closer the output tube is to the central axis, the further back its gearing must be in the Mechanism—though there is a certain amount of "interleaving" within the planetary mechanisms, so that they can share fixed gears and at the same time output at the right cosmological place in the co-axial tube system. With the lunar anomaly mechanism at the back of the Antikythera Mechanism, this output is the central arbor of the coaxial system. In the layering of the outputs from **b1** to the **Strap**, the *mean Sun* is furthest back (closest to **b1**), then come the *Nodes*, *Mercury* and *Venus*. Similarly, the layering of the outputs from the **Strap** to the **CP** are the *true Sun*, *Mars*, *Jupiter*, and *Saturn* (with some gears on the front of the **CP**)—see Fig. 4o for an image of the space available. The output tubes are layered in the CCO.

At first sight, it might appear that there are many options for our model, but in practice the number of options is strictly limited by the constraints defined by the available data, the period relations, the customary cosmology and the inscriptions. We cannot claim that there is a unique solution, since so much data are missing—though we have not found essentially different options for the system. We have attempted to optimize our solution by keeping the number of gears to a minimum, with a low overall tooth count that is consistent with mechanical requirements; a system that runs smoothly in mechanical terms; and a well-defined design layout—in other words, conforming to all our *Reconstruction Principles*.

6.2.4 Lunar Phase Mechanism

In our model, for the Moon phase we have used the simple differential with two gears, originally proposed[10]. This entails reversing the current orientation of the crown gear (Supplementary Fig. S16d) so that it points towards the central axis, rather than away from it. A close examination of the X-ray CT shows that the crown gear fits in the cut-out in the plate in this reverse orientation, though other authors disagree[61]. One justification is that it had mistakenly been placed in the wrong orientation, *"…having been taken apart and reassembled incorrectly"*[10]—possibly when the Mechanism was packed for transit on the Antikythera ship. This would mean that the gear **b0** that feeds the solar rotation into the Moon phase device was also misplaced, since it could not engage with the back of the crown gear. To get over this problem, an alternative with four gears was proposed[9], though it produced exactly the same output as the two-gear mechanism—unsatisfactory on the grounds of *Occam's Razor*. A highly ingenious proposal[61] maintains this four-gear system but suggests that two of the gears ran on eccentric axes and were linked by a pin-and-slot device, which operates in the period of the lunar month. The authors describe the idea: *"Aristarchus distinguished the dichotomy (when the boundary between the moon's dark and illuminated parts appears to be a straight line) from the quadrature (when the moon is exactly 90° elongation from the sun)."* These events are just a few degrees apart. We have not adopted this idea because it involves engineering the pin-and-slot at an improbably small scale; the effect of this system is barely visible on the 6 mm diameter Moon sphere; and our model inputs the less accurate *mean Sun* (not the *true Sun*) to the lunar phase mechanism—an inaccuracy barely perceptible on the 6 mm diameter Moon phase sphere.

6.2.5 Mean Sun, Nodes, Mercury & Venus

We can now bring together all the theory developed earlier about period relations and gear trains. In Supplementary Fig. S21 there is a layout for the *mean Sun*, the *Nodes of the Moon* and the *inferior planets* that fits in the space between **b1** and the **Strap** in closely-packed layers—as visualised in Fig. 5 and Supplementary Video S1. A previous model for the inferior planets[16], using similar principles for sharing gears and the same period relations for the inferior planets, does not conform to the physical evidence from the spokes on **b1** and the **Strap**. The *Nodes of the Moon* are introduced as an option on the grounds that they harmonize very well with the eclipse prediction scheme on the Back Dials. This would be realized by a double-ended *Dragon Hand* to indicate the positions of the *ascending* and *descending* nodes of the Moon. For each of the variable motions, such as those of Mercury and Venus, the mechanisms must be calibrated so that the phases of their epicycles are matched to observations on specified dates. To assist calibration, all our mechanisms will be designed so that their outputs are aligned with the cardinal directions, defined by the spokes on **b1**.

Supplementary Fig. S21 is a schematic diagram of the gearing with tooth counts to show how the period relations are calculated by the gears. The mechanisms in Fig. 6f are interleaved in three dimensions to ensure that the outputs are in the customary order.

a

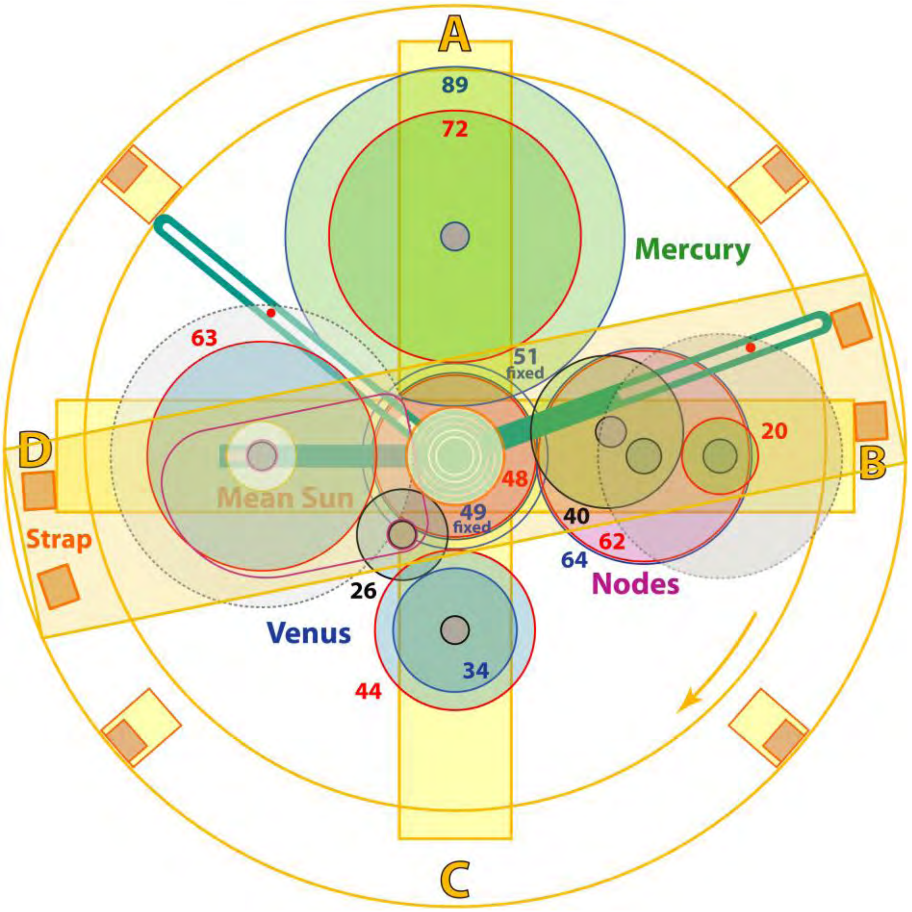

b

Spoke	*Function*	*Syn.*	*Years*	*Factors*	*Factors*	*Mult.*	*Epiyclic gear train*
**A**	**MERCURY**	**1513**	**480**	**17x89**	**2^5x3x5**	**x 3**	**51 ~ 72 + 89 ~ 40 ~ 20 ⊕ follower**
**B**	**NODES**	**98**	**93**	**2 x 7^2**	**3 x 31**	**x 32**	**49 ~ 62 + 64 ~ 48**
**C**	**VENUS**	**289**	**462**	**17^2**	**2x3x7x11**	**x 6**	**51 ~ 44 + 34 ~ 26 ~ 63 ⊕ follower**
**D**	**MEAN SUN**	**1**	**1**	**1**	**1**	**n/a**	**None**

Supplementary Fig. S21. Period relations and gear trains for mechanisms between b1 and the Strap.

(**a**) Schematic diagram of gearing between **b1** and the **Strap** for the *mean Sun*, the *Nodes of the Moon*, *Mercury* and *Venus*—see Supplementary Discussion S3. The mechanisms are interleaved, so that their outputs emerge in the CCO.

(**b**) The period relations and how they are realized in our proposed gear trains. **Blue** numbers relate to synodic cycles, **red** numbers to years and **turquoise** to variable motion.

Geometric parameters for these gear trains can be found in Supplementary Table 9. In Supplementary Fig. S22 it can be seen that there is a close match between the features in Fragments A and D and our proposed mechanisms between **b1** and the **Strap**. In Supplementary Video S1 there is an animated version of how this 3D system is constructed.

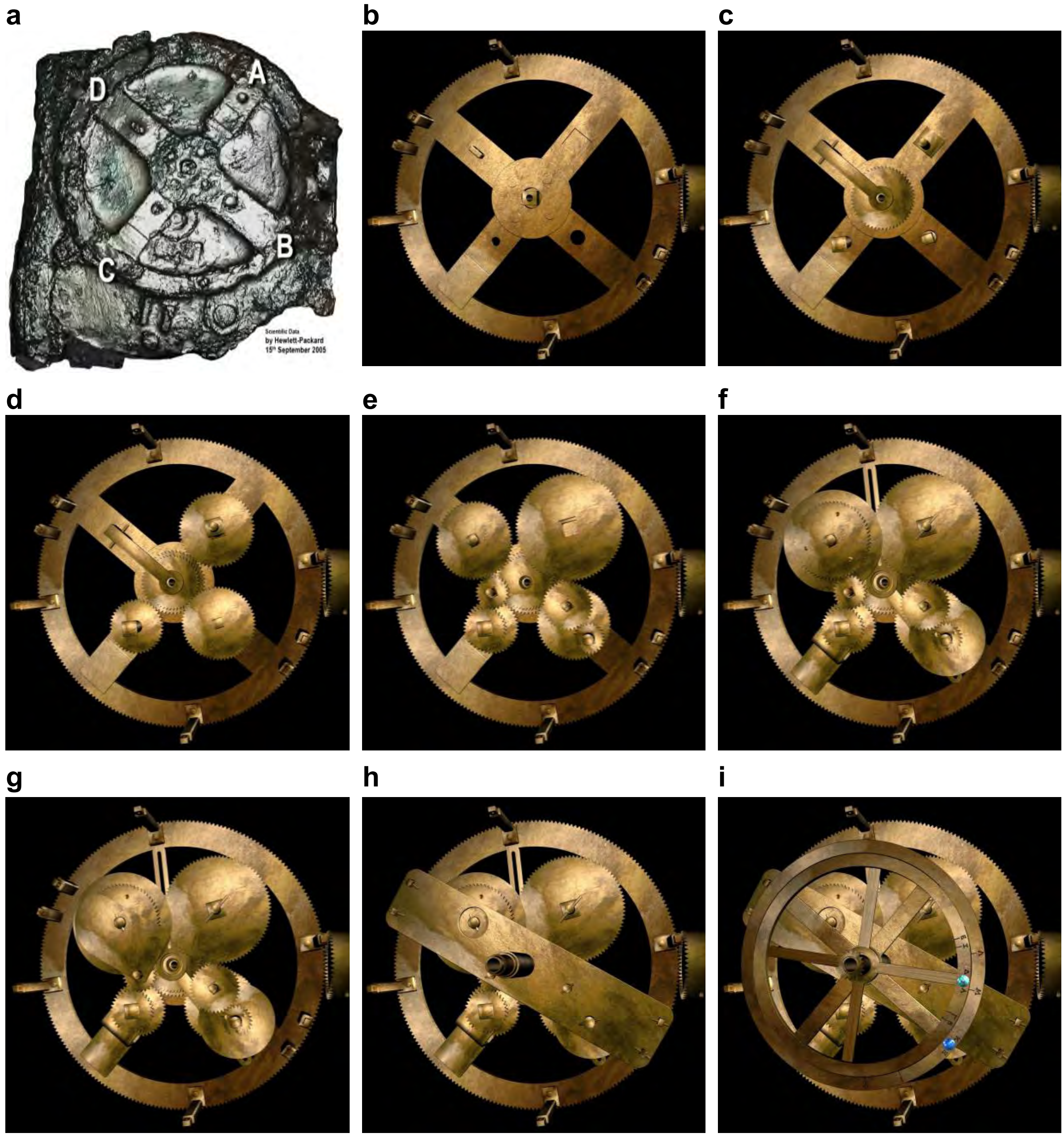

Supplementary Fig. S22. Mechanisms from b1 to the Strap: match between the evidence and the reconstruction.

(**a**) PTM of Fragment A with specular enhancement. *See large version in Supplementary Fig. S9.*

(**b**) Computer reconstruction of **b1**, with four short and four long pillars.

(**c**) **Centre**: fixed gear **51** for Mercury & Venus; Spoke A: arbor for **72** + **89** for Mercury; **Spoke B**: arbor for Nodes; Spoke C: arbor for **44** + **34** for Venus; Spoke D: Attachment and bar for mean Sun.

(**d**) **Centre**: fixed gear **49** for Nodes; Spoke A: **72** for Mercury; Spoke B: **62** for Nodes; Spoke C: **44** for Venus.

(**e**) Spoke A: **89** for Mercury; **Strap** (not shown): **40** ~ **24** for Mercury; Spoke C: **34** for Venus**; Strap** (not shown) **26** ~ **63** for Venus.

(**f**) Behind **Strap** (not shown): *disk*, *pin* and *follower* for Mercury (above Spoke B); Spoke C: bracket for **44** + **34** for Venus; *disk*, *pin* and *follower* for Venus (above Spoke D).

(**g**) *D-plate* for Venus behind **Strap** (not shown).

(**h**) **Strap** and *output tubes*.

(**i**) **Output rings** for Mercury & Venus display. Gear **b0** for input to *lunar phase mechanism*.

6.2.6 Mean Sun

For a *mean Sun* output, the obvious solution would be to have a tube come up from the centre of **b1**, nested on the lunar shaft. However, the fixed gears for the epicyclic systems for Venus and Mercury need to be held fixed somehow. They cannot be held fixed from above **b1** by any sort of strap from the sides of the mechanism since this would collide with

the pillars on **b1**. Hence the fixed gears need to be mounted on a tube through **b1**, fixed to the Main Plate and there is clear evidence for this in Fragment A. This then prevents fixing a tube for the mean Sun to the centre of **b1**. The solution is to attach the *mean Sun* via a simple bar and tube (Fig. 5a), fixed to the previously-unexplained block on Spoke D (Fig. 4e, Supplementary Fig. S12d). As can be seen in the X-ray CT, this block is pierced to accommodate a fixing pin. The bar bridges the fixed gears at the centre. Attached to the bar is an output tube that carries the mean Sun rotation to the lunar phase device, via a 20-tooth gear **b0** that matches the tooth count of the observed crown gear in the lunar phase device. This is the key idea that enables a ring system for the planetary outputs because it means that there can be a separate *true Sun* displayed in the ring system in the customary order.

6.2.7 Nodes

As described in the Main Text and Supplementary Discussion S4, the Nodes regress through the Zodiac at the approximate rate of - $5/93$ rotations per year. This is realized by the four-gear epicyclic gear train shown in Fig. 3a, Supplementary Fig. S21, with a fixed gear **49** at the centre. The epicyclic gears in this system, **62** + **64**, turn in a bearing at 27.0 mm from the centre. As described in Supplementary Discussion S4, a suitable choice of modules means that their arbor exactly matches the large bearing on Spoke B (Supplementary Figs. S12B, S22A-D). The output gear **48** has a tube attached that carries the rotation to the *Dragon Hand* on the front display.

6.2.8 Mercury & Venus

The two inferior planets have a similar design. Both planets share a fixed gear **51**. We will match our model to the evidence from Spoke C of **b1** in Fragment A (Supplementary Figs. S12C, S22D-H) and to the X-ray CT evidence of Fragment D (Supplementary Fig. S13), which we are confident includes the epicyclic components of Venus. These are reconstructed in Supplementary Fig. S14. The Venus gearing is illustrated in Fig. 5c. There is a presentation in Supplementary Discussion S5 of the arguments that lead to our proposed Venus gear train with the conclusion that it is *strongly indicated* by the evidence.

Our proposed gear train for the other inferior planet, Mercury, follows exactly the same 5-gear architecture (Fig. 3c) and is arranged on Spoke A and the **Strap** in an analogous way. A substantial difference is that the arbors and pins of the epicyclic gears do not need to be recessed below the level of the **Strap** because there is no mechanism directly in front of the **Strap** on the line of Spoke B. (The true Sun mechanism is mounted on the **CP** in line with Spoke D.) Another difference is that there is no supporting bracket for the arbor of **72** + **89** because **89** is too big, so that there is no space for a bracket on Spoke A. This means that the arbor is fixed and **72** + **89** are linked by a pipe that rotates on this fixed arbor. We assess the evidence for our proposed Mercury mechanism as very good.

There is a small circular feature in the form of a countersink on Spoke A, 41.4 mm from the centre and about 5.3 mm in diameter at the surface of the spoke, whose purpose is unclear. It could have carried a spacer to stabilize the large gear **89**.

6.3 True Sun, Superior Planets Mechanisms & Date

In this section, we discuss the detailed evidence and arguments that have led to our design for the *true Sun*, the *superior planets* and the *Date*. At first sight, it might appear that there are very many options for our model, but in practice the number of options is strictly limited by geometric considerations and the available prime factors in the period relations.

In Supplementary Fig. S23 the theoretical mechanisms developed earlier are mounted on the **CP**. One layer of gearing is in front of the **CP** and the other layers are in the space between the **Strap** and the **CP**. In Supplementary Video S1 the 3D construction of this system can be seen.

a

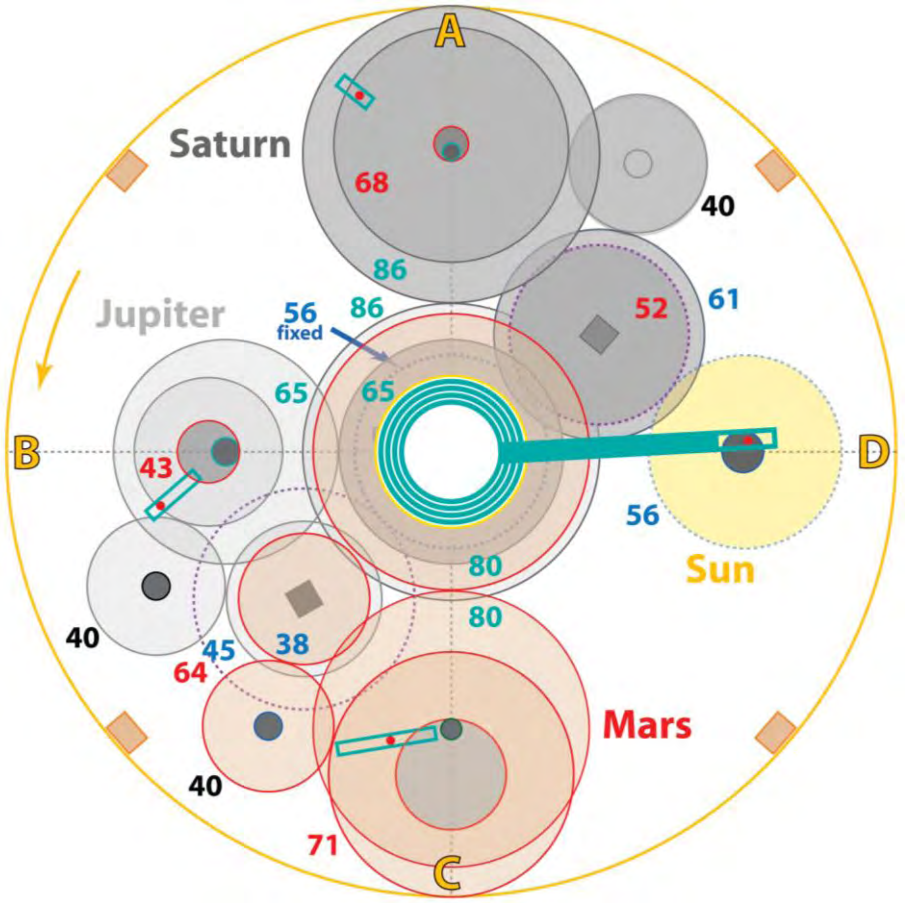

b

A	SATURN	427	442	7x61	2x13x17	x 8	56 ~ 52 + 61 ~ 40 ~ 68 ⊕ 86 ~ 86
B	JUPITER	315	344	3^2x5x7	2^3x43	x 8	56 ~ 64 + 45 ~ 40 ~ 43 ⊕ 65 ~ 65
C	MARS	133	284	7x19	2^2x71	x 16	56 ~ 64 + 38 ~ 40 ~ 71 ⊕ 80 ~ 80
D	TRUE SUN	1	1	1	1	x 56	56 ~ 52 ~ 56 ⊕ follower

Supplementary Fig. S23. Schematic Diagram, Period Relation and Gear Trains: true Sun, Superior Planets and Date.

(**a**) Gearing (seen from the back of the **CP**) between the **Strap** and the **CP**: true Sun and superior planets—see Supplementary Discussion S4. Gears with dotted outlines are in front of the **CP**. 7-gear indirect mechanisms are used for the superior planets. The eccentric pin-and-slot gears are arranged with their outputs aligned on the spokes of **b1** to aid calibration. With our choice of period relations, the whole system uses a single fixed gear 56, attached to a fixed sub-Plate behind the Front Plate. This also serves as a bearing for the whole output tube system. The second gears for each planet (shown with a dotted line) are mounted in front of the **CP**, their outputs being carried via arbors through the **CP** to the rest of the gear trains.

(**b**) Period relations and gear trains from Supplementary Discussion S3, 4.

The system is turned from a fixed gear 56 in the centre (Fig. 6a), which is rivetted to a fixed Sub-Plate (not shown). It should be noted that there are other possible solutions for the central gear, with tooth count divisible by 7, but we have adopted this one on the grounds that it is probably the smallest practical fixed gear that will allow a hole through its centre big enough to take all the nested output tubes, plus space to rivet it to the front plate. This scheme makes the system more robust mechanically and much simpler and easier to assemble than an earlier attempt with three fixed gears for the superior planets[9]. The initial gears in the systems are mounted in front of the **CP**. In Fig. 6a, the gear **52** to the north-west of 56 is shared between the *true Sun* and *Saturn*. It also meshes with the final epicyclic gear **56** of the true Sun mechanism. On the other side of the **CP** (Fig. 6b), there is a follower (Fig. 6b, c) that tracks an eccentric pin attached to the arbor for **56**, matching the ancient Greek theory of the true Sun. The output is carried by a tube to the front ring display for the true Sun. The other gear on the front of the **CP** is **64**, shared between *Mars* and *Jupiter*.

The remaining gears for the superior planets are laid out on the back of the **CP** (Fig. 6b). Clockwise from the *true Sun*, they are *Mars*, *Jupiter* and *Saturn* and they all follow the indirect 7-gear mechanisms, developed earlier (Fig. 3d). In Supplementary Fig. S23a there is a schematic diagram of the gearing with tooth counts to show how the period relations are calculated by the gears. Fig. 6d shows the mechanisms interleaved in three dimensions to ensure that the outputs are in the CCO. The period relations are from Supplementary Discussion S3 and we use the 7-gear *indirect mechanisms* for the superior planets from Figure 3, Supplementary Discussion S4. There is a choice of integer multipliers for the gear trains. It is easy to establish that a reasonable range is 7 – 10. Smaller than this and the gears are too small for making the pin-and-slot devices, particularly for Saturn with its very small offset; larger than 10 and the gears become too large to fit on the **CP** between the output tubes and the periphery of the **CP**. A previous model[9] required brackets to frame the mechanisms for the superior planets. Our model has no need for these, since the initial gears for the superior planets are at the front of the **CP** and the other gears at the back, stabilizing the arbors in the **CP**. The *Date* is represented by the *mean Sun* rotation. We do not continue the mean Sun output to turn a date pointer since it would then lie very close to the True Sun output and would confuse the readability of the planetary displays. Our *Date pointer* is a short detachable pointer, fixed to an outermost *Date* ring and extending to the Calendar Dial, whose presence proves that the Date pointer existed. This is attached to a tube, which is in turn secured to the front of the **CP**. This arrangement has the major mechanical advantage that the Date output tube, rigidly fixed to the **CP**, then supports all the nested tubes within and can turn in a substantial bearing in the Sub-Plate. With four outputs, to assist with calibration, the mechanisms have been orientated with outputs along the four cardinal axes, defined by the spokes on **b1**. Exploiting shared gears between trains creates a highly economical system, minimizing the number of gears: with an *economical* choice of period relations, the whole system is achieved with a single fixed gear **56** at the centre. This means that there are only 19 gears in the system. It would be very difficult to construct a system with fewer gears that calculates the more complex period relations.

This completes our model of the Cosmos gear system. There are 30 surviving gears of the Antikythera Mechanism: 27 in Fragment A and one in each of Fragments B, C, D. 29 of these gears include **a1**, **b1** and everything behind **b1**: previously accepted models have increased the number of gears to 35 for these systems. We add 34 gears for the system in front of **b1**. So, the total number of gears in our model is 35 + 34 = 69. In Fig. 6f, an exploded diagram illustrates how all these systems fit together and culminate in the ring system of outputs. This system is shown in Supplementary Videos S1, S2.

6.4 Cosmos Display

The *Cosmos Display* has far-reaching consequences for the astronomy shown, as described in the Main Text—Fig. 7, Supplementary Figs. S24, S25, Supplementary Video S3.

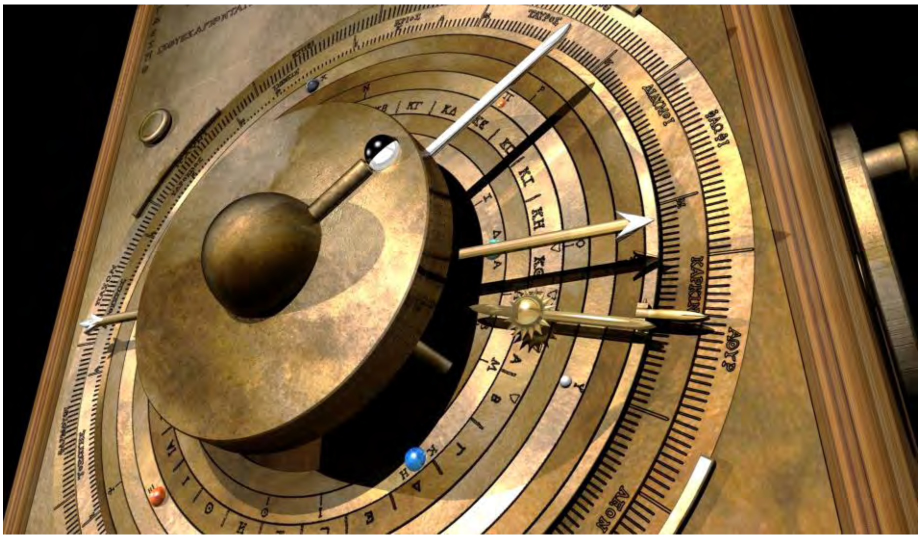

Supplementary Fig. S24. Front Face of the Antikythera Mechanism. Planets are marked by semi-precious stones. For superior planets, very small stones mark oppositions to distinguish between planetary rings.

6.4.1 Synodic Cycles of the Planets

The annular rings for the planets add another dimension to the display in their depiction of the synodic cycles of the planets—fully justifying the extra mechanical effort in devising the ring output system. The index letter system is described in the Main Text. The apparent mistakes in the FCI in the intervals between synodic events, which have been previously noted[12], may well result from uneven gearing, rather than mistakes in trigonometry or observations. There was no need for trigonometry either in the mechanical design or in the determination of the astronomical parameters (such as the sizes of the epicycles and the offsets) or in the inscriptions of the Antikythera Mechanism (Supplementary Discussion S3).

6.4.2 Pointer for the Date

Completing the display, a *Date pointer* is attached to a narrow date ring, which is turned by the outermost tube of the output system, fixed to the Circular Plate. It shows the date in the Egyptian calendar ring on the outside of the display. This movable ring enables additional days for leap years. A pin attached to the back of the scale would have engaged with one of 365 small holes drilled in the plate behind the scale and visible in the X-ray CT[7]. Every leap year, the pointer would stand still for a day, while the calendar scale would turn backwards to add a day every 4 years. So, each year elapsed on the Mechanism has 365¼ days, whereas each year on the Egyptian calendar scale has 365 days.

6.4.3 Lunar Cycles

The Antikythera Mechanism embraces all four of the basic notions of month: *sidereal*, *synodic*, *draconitic* and *anomalistic*[14]. The *sidereal month* is shown by the Moon pointer on the Zodiac scale; the *synodic month* by the lunar phase sphere, by the Age of the Moon scale and by the scale divisions of the Metonic and Saros Dials; the *draconitic month* by the cycle of the lunar pointer relative to the Dragon Hand The *anomalistic month* does not appear directly, but it is critically involved in the calculation of the lunar anomaly output[7].

6.4.4 The Kallippic Cycle

The 19-year Metonic calendar has 6,940 days, implying a year-length of $365\frac{5}{19}$ days. The improved 76-year Kallippic calendar has 4 x 6,940 - 1 = 27,759 days, implying a year-length of 365¼ days. The leap-year system on the Antikythera Mechanism ensures that it automatically followed the Kallippic Cycle, not the Metonic Cycle, because of the distribution of the 19 leap days of the 76-year Kallippic period over the four Metonic sub-periods: three of these must have had five leap days and one must have had only four. This accounts for the so-called *lost day* in the Kallippic period, compared with four Metonic periods. Previously, it was asserted that, *"The extant inscriptions give no hint of which day was to be omitted…"*[24]*;* or in another publication that there was need of *"an additional rule"* to determine the lost day[27]. None of this was necessary: the leap year system loses the day automatically by not adding a fifth leap day to one of the Metonic sub-periods, depending only on which year of the Kallippic period is designated as the first leap year. For example, if it is Year 4, then in the first 19-year Metonic sub-period leap days are added in Years 4, 8, 12 and 16—only four leap days are added—whereas in each of the subsequent three Metonic sub-periods five leap days are added. In any 76-year period, the number of days is 76 x 365 + 19 = 27,759, as required for the 76-year Kallippic calendar. There is no *lost day*: it is simply that a leap day is *not added*.

6.4.5 Justifications of Cosmos Display

Our scheme for the extensive display of astronomical information on the Cosmos display—such as the synodic cycles of the planets—is fully supported by our *Reconstruction Principles*. Further justification comes from highly ingenious aspects of other dials:

- The Metonic Dial is laid out as a five-turn spiral and this enables the *excluded days* for the *hollow* 29-day months of the Metonic Calendar to be automatically defined by the excluded day numbers inscribed on the inside of the Metonic spiral[8]. These excluded day numbers then apply to all five months on the same radius. Not only does this create an automatic scheme for the excluded days, but it also improves on the scheme, described by Geminos[31] in the 1st century BC, of excluding every 64th day, because it is more regular over the boundaries between Metonic periods.

- The Saros Dial is a four-turn spiral over the 223 lunar months of the Saros period, so each quarter-turn of the pointer is synchronized with the *New Moon Cycle*, which defines the angular diameter of the Moon at New Moon. The angle of the Saros pointer automatically indicates whether a central eclipse is *total*, *annular* or *hybrid*[8].
- The *Exeligmos Dial* automatically shifts the eclipse times by eight hours after each Saros period[7]: a neat solution to the eclipse times over successive Saros periods.
- The leap year system with a moveable *Calendar Dial*[1] is highly inventive and means that the Antikythera Mechanism automatically follows the Kallippic Cycle, with its year length of 365¼ days.

6.4.6 The Antikythera Mechanism as its own division engine

We proposed that the synodic divisions on the planetary rings were determined by the Antikythera Mechanism itself. Previously, it was suggested that the divisions of the Saros and Metonic Dials on the Back Plate could have been laid out mechanically by the Mechanism itself[7]. The scale divisions would be inscribed sequentially: the Mechanism turned to the next New Moon, as shown by coincidence of the Sun and Moon pointers, then wound forwards two days to allow for the interval between New Moon and First Crescent Moon. The scale division would be inscribed, and the process repeated for all the scale divisions of the Saros & Metonic Dials. Thus, the Mechanism could serve as its own division engine when setting out the divisions of the Back Dials—perhaps explaining why the Saros Dial divisions are so uneven because of unevenly divided gears. The eclipse limits on the Sun ring creates the intriguing idea that a mechanical process could have been used to determine whether a lunar or solar glyph should be inscribed on the Saros Dial. However, the arithmetic calculation, previously described[23], for determining glyphs is so simple that it might have been more certain than using the Mechanism itself with its uneven gears.

6.5 Conclusions

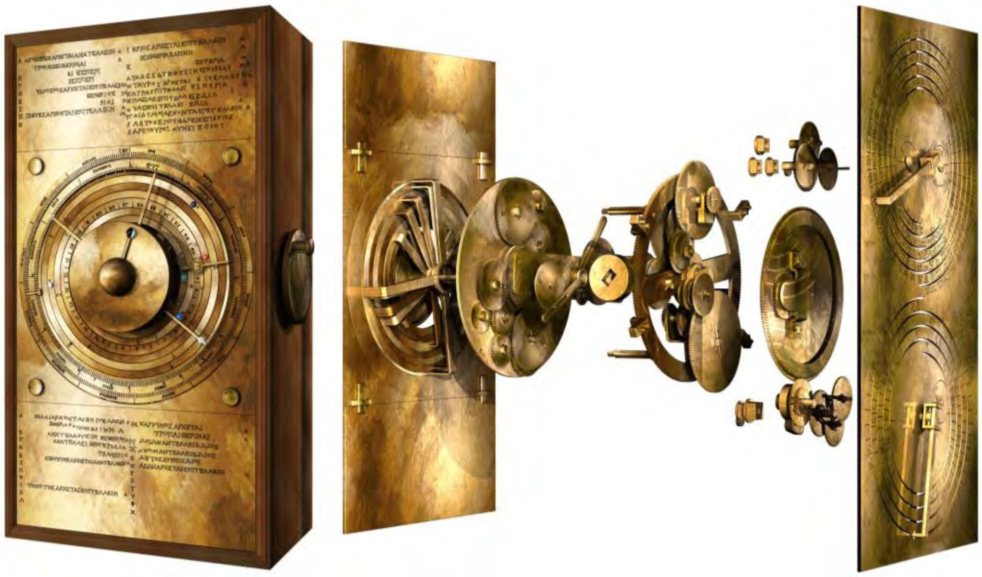

Supplementary Fig. S25. Front face and exploded diagram of all the components.

In Supplementary Videos S1, S2, S3, animations reveal the 3D structure of our Cosmos systems. We have predicted the period relations built into the planetary mechanisms; we have integrated everything into the very tight spaces defined by the evidence; we have interleaved the mechanisms and we have designed the output display to conform to the inscriptions and the customary ancient Greek cosmology. Based on more than a hundred years of research and with many original contributions, we have created a solution to this intricate 3D puzzle, with everything conforming to all the evidence.

7. Image Credits & Software

7.1 Image Credits

All necessary permissions have been obtained for use of the images in this publication.

Figure	*Pt*	*Component*	*Copyright/Credit*	*Date*
**Fig. 1**		X-ray CT	Nikon X-Tek Systems	2005
		Graphics	Tony Freeth	2019
**Fig. 2**	**a-e**	Graphics	Tony Freeth	2019
**Fig. 3**	**a-d**	Gear diagrams	Tony Freeth, David Higgon	2019
	**e-f**	Gear trains	Tony Freeth, David Higgon	2019
**Fig. 4**	**a, i**	Photos	Nat. Arch. Museum in Athens	2005
	**b-d, f, j-l**	X-ray CT	Nikon X-Tek Systems	2005
	**e, g**	PTM	Hewlett-Packard	2005
	**m-p**	Computer model	Tony Freeth, David Higgon	2019
**Fig. 5**	**a-e**	Computer model	Tony Freeth, David Higgon	2019
	**b, d**	X-ray CT	Nikon X-Tek Systems	2005
	**e, f, h, i, k, l**	Computer model	Tony Freeth, David Higgon	2020
	**g, j**	PTM	Hewlett-Packard	2005
**Fig. 6**		Computer model	Tony Freeth, David Higgon	2020
**Fig. 7**		Computer model	Tony Freeth, David Higgon	2020
**Fig. 8**		Graphics	Tony Freeth	2019
**Supp. Table S1**		Text & Translation	Bitsakis, Y., Jones, A[13,i]	2016
**Supp. Table S2**		Graphic	Tony Freeth	2019
**Supp. Table S3**		Graphic	Aris Dacanalis	2019
**Supp. Table S4**		Graphic	Aris Dacanalis	2019
**Supp. Table S5**		Graphic	Tony Freeth	2019
**Supp. Table S6**		Graphic	Tony Freeth	2019
**Supp. Table S7**		Graphic	Tony Freeth	2019
**Supp. Table S8**		Graphic	Tony Freeth	2019
**Supp. Fig. S1**		PTM	Hewlett-Packard[ii]	2005
**Supp. Fig. S2**	**a**	Reproduction	British Library Board[iii]	
	**b**	Reproduction	Bodleian Library[iv]	
	**c**	Reproduction	British Library Board[v]	
	**d**	Prague	Max Pixel[vi]	
	**e**	Lyon, France	Alex Quici	
	**f**	Cremona, Italy	Royalty Free[vii]	2011
**Supp. Fig. S3**	**a**	Bracelets	Walters Art Museum[viii]	
	**b**	Jewelled necklace	Anonymous owner[ix]	

i Fair use of short extract for research purposes.
ii Data gathered by Tom Malzbender & team in 2005. Published with permission, ©2005 Hewlett-Packard.
iii Email permission 24/04/19. Shelfmark: Cotton MS Domitian I. Item number: f.23v: http://www.bl.uk/manuscripts/Viewer.aspx?ref=cotton_ms_domitian_a_i_f002r
iv Non-commercial use: https://digital.bodleian.ox.ac.uk/inquire/p/d2fc67d5-b2a5-4851-9196-e84ef147ef15
v Email permission 24/04/19. Shelfmark: Egerton MS 2781: https://www.bl.uk/collection-items/the-neville-of-hornby-hours
vi CC0: https://www.maxpixel.net/Town-Hall-Storm-Clock-Prague-Czech-Republic-2011375
vii CC BY-SA 3.0: https://commons.wikimedia.org/wiki/Category:Clock_of_the_Torrazzo_di_Cremona#/media/File:Cremona-Orologio_astronomico_sul_Torrazzo_perspec.jpg
viii CC0: https://art.thewalters.org/detail/77272/bracelets-from-the-olbia-treasure/
ix Email permission 04/05/19.

	**c**	Necklace and earrings	Metropolitan Museum of Art, NY[x]	1994
**Supp. Fig. S4**	**a**	X-ray CT	Nikon X-Tek Systems	2005
	**b**	X-ray CT	Nikon X-Tek Systems	2005
	**c**	X-ray CT	Nikon X-Tek Systems	2005
**Supp. Fig. S5**	**a**	Photo	Nat. Arch. Museum in Athens	2005
	**b**	X-ray CT	Nikon X-Tek Systems	2005
	**c**	Computer model	Tony Freeth	2019
**Supp. Fig. S6**	**a-c**	Graphics	Tony Freeth	2019
**Supp. Fig. S7**		Graphic	Tony Freeth	2019
**Supp. Fig. S8**		Graphic	Tony Freeth	2019
**Supp. Fig. S9**		PTM	Hewlett-Packard (public domain)	2005
**Supp. Fig. S10**	**a, b**	Photos	Nat. Arch. Museum in Athens	2005
**Supp. Fig. S11**	**a-i**	X-ray CT	Nikon X-Tek Systems	2005
**Supp. Fig. S12**	**a-d**	Photos	Nat. Arch. Museum in Athens	2005
	**a-c**	PTM	Hewlett-Packard (public domain)	2005
	**b-d**	X-ray CT	Nikon X-Tek Systems	2005
**Supp. Fig. S13**	**a-b**	Photos	Nat. Arch. Museum in Athens	2005
	**c-j**	X-ray CT	Nikon X-Tek Systems	2005
**Supp. Fig. S14**		Computer model	UCL Antikythera Research Team	2019
**Supp. Fig. S15**	**a-f**	X-ray CT	Nikon X-Tek Systems	2005
**Supp. Fig. S16**	**a-f**	X-ray CT	Nikon X-Tek Systems	2005
**Supp. Fig. S17**	**a-d**	Notes, Photo	Bayerische Staatsbibliothek, Munich	1906
**Supp. Fig. S18**		Model, Photo	Model: M. Wright. Photo: T. Freeth	2002
**Supp. Fig. S19**		Computer model	Tony Freeth	2012
**Supp. Fig. S20**		Graphic	Tony Freeth	2019
**Supp. Fig. S21**	**a**	Graphic	Tony Freeth, David Higgon	2019
**Supp. Fig. S22**	**a**	PTM	Hewlett-Packard (public domain)	2005
	**b-i**	Computer model	UCL Antikythera Research Team	2019
**Supp. Fig. S23**	**a**	Graphic	Tony Freeth, David Higgon	2019
**Supp. Fig. S24**		Computer model	UCL Antikythera Research Team	2019
**Supp. Fig. S25**		Computer model	UCL Antikythera Research Team	2019
**Supp. Video S1**		Animation	Tony Freeth, CC BY-NC license	2019
**Supp. Video S2**		Animation	Tony Freeth, CC BY-NC license	2019
**Supp. Video S3**		Animation	Tony Freeth, CC BY-NC license	2019

7.2 Software

All X-ray CT images were analysed and output using *Volume Graphics VGStudio Max 2.1*.

All PTM images were output from *Hewlett-Packard PTM Viewer*.

All the computer models of the Antikythera Mechanism were produced in *Newtek Lightwave 3D 9.6.2*, as were the *Supplementary Videos*, which had captions added in *Final Cut Pro 7.0.3*. The videos were then compressed in *Handbrake 1.3.2* to meet maximum file sizes.

The figures and tables were made using *Adobe Illustrator CS5.1*, *Adobe Photoshop CS5.1*, *Microsoft Word and Excel 365*. No inappropriate image manipulations were used.

[x] The Bothmer Purchase Fund and Lilac Acheson Wallace Gift, 1994: https://www.metmuseum.org/toah/works-of-art/1994.230.4-.6/

8. References & Supplementary References

1 Rehm, A. Notizbuch (unpublished notebooks), research manuscripts and photographs from 1905-1906. Bayerische Staatsbibliothek, Munich, Germany. Rehmiana III/7 and III/9 (1905-1906).

2 Price, D. de S. *Gears from the Greeks: The Antikythera Mechanism — A Calendar Computer from ca. 80 BC.* American Philosophical Society. *Trans Am. Phil. Soc. New Ser.* **64,** 1–70. Philadelphia (1974); reprinted by *Science History Publications*, New York (1975).

3 Wright, M.T. A Planetarium Display for the Antikythera Mechanism, *Horological Journal*, Vol **144,** Issue **5,** pp. 169 – 173 (2002).

4 Wright, M.T. Counting Months and Years: The Upper Back Dial of the Antikythera Mechanism. *Bulletin of the Scientific Instrument Society*, No. **87** (2005).

5 Wright, M.T. The Antikythera Mechanism: a New Gearing Scheme. *Bulletin of the Scientific Instrument Society*, No. **85** (2005).

6 Wright, M.T. Epicyclic gearing and the Antikythera Mechanism, Part II. *Antiquarian Horology*, Vol. **29,** No. **2,** Dec. 2005 (2005).

7 Freeth, T. et al. Decoding the ancient Greek astronomical calculator known as the Antikythera Mechanism. *Nature* **444,** 587–591 (2006).

8 Freeth, T., Jones, A., Steele, J.M., Bitsakis, Y. Calendars with Olympiad display and eclipse prediction on the Antikythera Mechanism. *Nature* **454,** 614-617 (2008).

9 Freeth, T., Jones, A. The Cosmos in the Antikythera Mechanism. *ISAW Papers* **4**, February 2012. http://dlib.nyu.edu/awdl/isaw/isaw-papers/4/ (2012).

10 Wright, M.T. The Antikythera Mechanism and the early history of the moon phase display. *Antiquarian Horology* **29**, 319-329 (2006).

11 Carman, C, Thorndike, A., Evans, J. On the pin-and-slot device of the Antikythera Mechanism, with a new application to the superior planets. *JHA* **43,** 93-116 (2012).

12 Anastasiou, M. et al. The Inscriptions of the Antikythera Mechanism, Paper 6: The Front Cover Inscription. *Almagest*: Vol. **VII,** Issue **1** (2016).

13 Bitsakis, Y., Jones, A. The Inscriptions of the Antikythera Mechanism, Paper 5: The Back Cover Inscription. *Almagest*: Vol. **VII**, Issue **1** (2016).

14 Neugebauer, O. *A History of Ancient Mathematical Astronomy*. Springer, New York (1975).

15 Neugebauer, O. Astronomical Cuneiform Texts, Vols I – III. 1st edition, Lund Humphries London (1974). Reprinted by Springer-Verlag (1983).

16 Wright, M.T. The Antikythera Mechanism: Compound Gear-Trains for Planetary Indications. *Almagest*, Vol. **4,** Issue **2**. https://doi.org/10.1484/J.ALMAGEST.1.103717 (2013).

17 Fowler, D. H. *The Mathematics of Plato's Academy: a New Reconstruction.* Clarendon Press, Oxford (1987).

18 Fowler, D. H. Ratio in Early Greek Mathematics. *Bull Amer. Math. Soc.*, Vol **1,** No. **6,** November (1979).

19 Lawlor, R, Lawlor, D. *Theon of Smyrna: Mathematics Useful for Understanding Plato*, III, 15., translated from the 1892 Greek/French edition of J. Dupuis (1892).

20 King, H. C. & Millburn, J. R. *Geared to the Stars: The Evolution of Planetariums, Orreries and Astronomical Clocks.* University of Toronto Press (1978).

21 Freeth, T. The Antikythera Mechanism: 2. Is it Posidonius' Orrery? *Mediterranean Archaeology & Archaeometry*, Vol. **2,** No. **2** (2002).

22 Pakzad, A. et al. Improved X-ray computed tomography reconstruction of the largest fragment of the Antikythera Mechanism, an ancient Greek astronomical calculator. *PLOS ONE*, November 2018. https://doi.org/10.1371/journal.pone.0207430

23 Freeth, T. Eclipse prediction on the ancient Greek astronomical calculating machine known as the Antikythera Mechanism. *PLOS ONE* (2014). doi.org/10.1371/journal.pone.0103275

24 Anastasiou, M. et al. The Inscriptions of the Antikythera Mechanism, Paper 4: The Back Dial and Back Plate Inscriptions. *Almagest*: Vol. **VII,** Issue **1** (2016).

25 Bitsakis, Y., Jones, A. The Inscriptions of the Antikythera Mechanism, Paper 3: The Front Dial and Parapegma Inscriptions. *Almagest*: Vol. **VII**, Issue **1** (2016).

26 Van Brummelen, G. *The Mathematics of the Heavens and the Earth: The Early History of Trigonometry.* Princeton (2009).

---

27 Jones, A. *A Portable Cosmos: Revealing the Antikythera Mechanism, Scientific Wonder of the Ancient World*. New York: Oxford University Press (2017).

28 Hultsch, F.O. *Pappi Alexandrini Collectionis Quae Supersunt. Edition of Pappus in three Volumes*, Apud Weidmannos (1878).

29 Plato. *Republic, Volume II: Books 6-10*. Loeb Classical Library. Translators: Emlyn-Jones, C., Preddy, W. (2013).

30 Guthrie, W.K.C. *Aristotle On the Heavens.* Loeb Classical Library, Harvard University Press, Cambridge, Mass. USA (1939).

31 Evans, J., Berggren, J.L. *Geminos's Introduction to the Phenomena: A Translation and Study of a Hellenistic Survey of Astronomy.* Princeton University Press, Princeton and Oxford (2006).

32 Bubenik, V. Compound Tenses (Hellenistic Greek), in: Encyclopedia of Ancient Greek Language and Linguistics, General Editor: Georgios K. Giannakis. http://dx.doi.org/10.1163/2214-448X_eagll_COM_00000070

33 Lavidas, N. Null vs. cognate objects and language contact: Evidence from Hellenistic Greek. Acta Linguistica Hafniensia. 44, 142-168 (2014). https://www.tandfonline.com/doi/abs/10.1080/03740463.2013.779079

34 Wright, M.T. The Antikythera Mechanism reconsidered, *Interdisciplinary Science Reviews*, Vol. **32**, No. **1** (2007).

35 Toomer, G. J. *Ptolemy's Almagest* (transl. Toomer, G. J.). Princeton Univ. Press, Princeton, New Jersey (1998).

36 Baillie, G. H., Lloyd, H. A. and Ward, F.A.B. *The Planetarium of Giovanni de Dondi*. Wadhurst, Sussex, UK (1974).

37 Hartner, W. The Pseudoplanetary Nodes of the Moon's Orbit in Hindu and Islamic Iconographies, *Ars Islamica*, Vol. **5**, No. 2, pp. 112-154 (1938).

38 Evans, J. The Astrologer's Apparatus: A Picture of Professional Practice in Greco-Roman Egypt, *Journal for the History of Astronomy*, **35,** 1-44 (2004).

39 Crane, G. R. Ed. *Perseus Digital Library*. Tufts University. http://www.perseus.tufts.edu (accessed April 17, 2012).

40 Wright, M.T. In the Steps of the Master Mechanic, *Η Αρχαία Ελλάδα και ο Σύγχρονος Κόσμος (Ancient Greece and the Modern World)*. Patras: University of Patras, 86-97 (2003).

41 Evans, J. On the function and the probable origin of Ptolemy's equant. *American Journal of Physics* **52**, 1080-1089 (1984).

42 Evans, J. *The History & Practice of Ancient Astronomy*. Oxford University Press (1998).

43 Van der Waerden, B. L. *Science Awakening II: The Birth of Astronomy*. Springer, (1974).

44 Hunger, H., Pingree, D, Edwin, D. Astral Sciences in Mesopotamia. *Handbuch der Orientalistik*, Volume **44,** Koninklije Brill (1999).

45 Kugler, F.X. Sternkunde und Sterndienst in Babel: *Babylonische Planetenkunde Vol. 1*. Aschendorff (1907).

46 Brown, D. *Mesopotamian Planetary Astronomy-Astrology*. Styx (2000).

47 Neugebauer, O., Sachs, A. Some Atypical Astronomical Cuneiform Texts. *Journal of Cuneiform Studies* **21,** 183-218 (1967).

48 Ossendrijver, M. *Babylonian Mathematical Astronomy: Procedure Texts*. Springer (2012).

49 Rochberg, F. *Before Nature: Cuneiform Knowledge and the History of Science*. The University of Chicago Press (2016).

50 Swerdlow, N.M. *The Babylonian Theory of the Planets*. Princeton University Press (1998).

51 Neugebauer, O. Planetary Motion. P. Mich 149, *Bull American Society of Papyrologists* **ix,** 19 - 22 (1972).

52 Gray, J. M. K. A *Study of Babylonian Goal-Year Planetary Astronomy*, PhD Thesis, Durham University. http://etheses.dur.ac.uk/101/1/A_Study_of_Babylonian_Goal-Year_Astronomy.pdf?DDD25+ (2009).

53 Jones, A. *Astronomical Papyri from Oxyrhynchus*, Memoirs of the American Philosophical Society **233** (1999).

54 Ossendrijver, M. Ancient Babylonian astronomers calculated Jupiter's position from the area under a time-velocity graph. *Science* 29 Jan 2016: Vol. 351, Issue 6272, pp. 482-484 (2016). doi: 10.1126/science.aad8085

55 Duke, D. Mean Motions in Ptolemy's "Planetary Hypotheses". *Archive for History of Exact Sciences*, Vol. **63,** No. **6** (2009).

56 Svoronos, I.N. *Ὁ Θησαυρὸς τῶν Ἀντικυθήρων* (The Antikythera Hoard). Athens. Republished in Svoronos, I.N. (1908), *Τὸ ἐν Ἀθήναις Ἐθνικὸν Μουσεῖον* (The National Museum of Athens). Athens (1903).

57 Price, D. de S. An Ancient Greek Computer. *Scientific American*, **200,** 66 (1959).

58 Evans, J., Carman, C., Thorndike, A. Solar Anomaly and Planetary Displays in the Antikythera Mechanism. *JHA*, Vol. 41, Issue 1, p. 1-40 (2010).

59 Carman, C., Evans, J. Babylonian Solar Theory on the Antikythera Mechanism. *Archive for History of Exact Sciences* 73 (6):619-659 (2019).

60 Wright, M.T. Archimedes, Astronomy, and the Planetarium. Archimedes in the 21st Century, *Proceedings of a World Conference at the Courant Institute of Mathematical Sciences*. doi.org/10.1007/978-3-319-58059-3_7 (2017).

61 Carman, C., Di Cocco, M. The Moon Phase Anomaly in the Antikythera Mechanism. *ISAW Papers* **11**. http://dlib.nyu.edu/awdl/isaw/isaw-papers/11/ (2016).

© The Authors 2021
